# Contemporary Remotely Sensed Data Products Refine Invasive Plants Risk Mapping in Data Poor Regions

**DOI:** 10.3389/fpls.2017.00770

**Published:** 2017-05-15

**Authors:** Tuyet T. A. Truong, Giles E. St. J. Hardy, Margaret E. Andrew

**Affiliations:** ^1^Environmental and Conservation Sciences, School of Veterinary and Life Sciences, Murdoch University, PerthWA, Australia; ^2^Faculty of Environment, Thai Nguyen University of Agriculture and ForestryThai Nguyen, Vietnam

**Keywords:** non-native invasive species, invasibility, MaxEnt, MODIS, native invasive species, species distribution modeling, Southeast Asia

## Abstract

Invasive weeds are a serious problem worldwide, threatening biodiversity and damaging economies. Modeling potential distributions of invasive weeds can prioritize locations for monitoring and control efforts, increasing management efficiency. Forecasts of invasion risk at regional to continental scales are enabled by readily available downscaled climate surfaces together with an increasing number of digitized and georeferenced species occurrence records and species distribution modeling techniques. However, predictions at a finer scale and in landscapes with less topographic variation may require predictors that capture biotic processes and local abiotic conditions. Contemporary remote sensing (RS) data can enhance predictions by providing a range of spatial environmental data products at fine scale beyond climatic variables only. In this study, we used the Global Biodiversity Information Facility (GBIF) and empirical maximum entropy (MaxEnt) models to model the potential distributions of 14 invasive plant species across Southeast Asia (SEA), selected from regional and Vietnam’s lists of priority weeds. Spatial environmental variables used to map invasion risk included bioclimatic layers and recent representations of global land cover, vegetation productivity (GPP), and soil properties developed from Earth observation data. Results showed that combining climate and RS data reduced predicted areas of suitable habitat compared with models using climate or RS data only, with no loss in model accuracy. However, contributions of RS variables were relatively limited, in part due to uncertainties in the land cover data. We strongly encourage greater adoption of quantitative remotely sensed estimates of ecosystem structure and function for habitat suitability modeling. Through comprehensive maps of overall predicted area and diversity of invasive species, we found that among lifeforms (herb, shrub, and vine), shrub species have higher potential invasion risk in SEA. Native invasive species, which are often overlooked in weed risk assessment, may be as serious a problem as non-native invasive species. Awareness of invasive weeds and their environmental impacts is still nascent in SEA and information is scarce. Freely available global spatial datasets, not least those provided by Earth observation programs, and the results of studies such as this one provide critical information that enables strategic management of environmental threats such as invasive species.

## Introduction

Invasive plants have emerged as a serious problem for global biodiversity. Their infestations can lead to the extinction (Groves et al., 2003) and endangerment (Wilcove et al., 1998; Pimentel et al., 2005) of native species and the alteration of ecosystem processes (Vitousek and Walker, 1989; Simberloff, 2000). Although invasive species that are introduced to a region receive the greatest attention, it is not necessary for a species to be non-native to be invasive. Invasive species are defined as those that are expanding their range (Valéry et al., 2008). Under global climate change and human disturbance, some native species have also become aggressive invasive weeds (Avril and Kelty, 1999; Wang et al., 2005; Hooftman et al., 2006; Valéry et al., 2009; Le et al., 2012). Given the large impacts that invasive species can have and the limited possibilities for eradication, early detection and prevention of the establishment of invasive species should be a priority in conservation policies (Genovesi, 2005). Identification of areas that are at potential invasion risk, to either non-native or native invasive species, can be an effective way to guide efficient management and prevent further incursion (Kulhanek et al., 2011).

Species distribution models (SDMs) are currently a popular method for predicting the geographic distribution of species (Peterson, 2006). They are developed statistically from the known occurrences of the species and characteristics of the environment to identify similar suitable habitats and, thereby, predict the geographic distribution in unknown regions (Guisan and Zimmermann, 2000; Peterson and Vieglais, 2001; Peterson, 2006; Pearson, 2010). Given these modest data requirements, they are especially useful in cases of poorly studied taxa (Kearney and Porter, 2009). Therefore, SDMs have become an important tool to investigations of invasibility that aim to predict the potential distributions of invasive species (Peterson, 2003; Thuiller, 2005). Since the early study of Peterson et al. (2003) in predicting the potential distribution of four invasive plants in North America, SDMs have been increasingly and widely applied all over the world to predict biological invasions (Guisan and Thuiller, 2005; Underwood et al., 2013), especially exotic plants (Zhu et al., 2007; Andrew and Ustin, 2009; Barik and Adhikari, 2011; Fernández et al., 2012; Rameshprabu and Swamy, 2015). In SDMs, the environmental variables used vary at different scales (Bradley et al., 2012). At regional to continental scales, forecasts of invasion risk are often mainly driven by climatic factors (Pearson and Dawson, 2003). Predictions at a finer scale and in landscapes with less topographic variation may require predictors that capture biotic processes (e.g., vegetation productivity) and local abiotic conditions (e.g., topography, soil type) (Pearson and Dawson, 2003). However, continuous spatial measurements of these finer-scaled environmental variables are difficult to acquire at large spatial extent (Bradley et al., 2012).

Contemporary remote sensing (RS) now provides widely available data products at multiple spatial and temporal resolutions that characterize a range of ecologically relevant patterns and processes (Andrew et al., 2014). These data can be used to measure habitat properties over a larger area than can easily be covered by field surveys (Estes et al., 2008) and augment the array of spatial environmental variables available to SDMs to characterize abiotic and biotic niche axes beyond simply climatic factors. **Table 1** provides an overview of the remotely sensed information that has been incorporated into SDMs as environmental predictor variables, to date, giving an indication of the evenness of research efforts and the capabilities of RS that are still relatively under-utilized. The most commonly used variable extracted from RS data is topography/elevation (42% of 39 reviewed studies that have developed SDMs of plant species using RS predictors). Besides, other abiotic predictors have been developed such as remotely sensed estimates of climate and weather, including surface temperature from sensors such as MODIS and rainfall estimates from TRMM and, more recently, the Global Precipitation Measurement mission, although studies applying these predictors are limited (**Table 1**). Soil properties, one of the most important factors for plant distributions and species invasion (Radosevich et al., 2007), is rarely studied (He et al., 2015), although several recent studies have explored the use of remotely sensed indicators of soil characteristics in SDMs (**Table 1**).

**Table 1:** Applications of remote sensing data as environmental variables in plant distribution models.

Predictor variables	RS data source	Reference
**Abiotic predictors**		
Topographic data/elevation	ASTER, Quickbird-2 and WorldView-2, LiDAR, SRTM	Rew, 2005; Bradley and Mustard, 2006; Buermann et al., 2008; Hoffman et al., 2008; Parviainen et al., 2008, 2013; Prates-Clark et al., 2008; Saatchi et al., 2008; Andrew and Ustin, 2009; Zellweger et al., 2013; Pottier et al., 2014; Pradervand et al., 2014; Questad et al., 2014; van Ewijk et al., 2014; Pouteau et al., 2015; Campos et al., 2016
Climate observations	MODIS, TRMM, NASA	Saatchi et al., 2008; Waltari et al., 2014; Deblauwe et al., 2016
Soil properties	Landsat, MODIS	Parviainen et al., 2013; Wang et al., 2016
Other physical variables (water, fire)	MODIS, NASA	Stohlgren et al., 2010; Cord and Rödder, 2011; Pau et al., 2013; Cord et al., 2014a
**Land cover/land use**	MODIS, Landsat	Pearson et al., 2004; Thuiller et al., 2004; Stohlgren et al., 2010; Morán-Ordóñez et al., 2012; Wilson et al., 2013; Cord et al., 2014b; Sousa-Silva et al., 2014; Tuanmu and Jetz, 2014; Gonçalves et al., 2016
**Vegetation productivity**		
Normalized difference vegetation index (NDVI)	Landsat, SPOT, MODIS	Morisette et al., 2006; Zimmermann et al., 2007; Prates-Clark et al., 2008; Evangelista et al., 2009; Feilhauer et al., 2012; Engler et al., 2013; Parviainen et al., 2013; Schmidt et al., 2013; Zellweger et al., 2013; van Ewijk et al., 2014
Leaf area index (LAI)	MODIS	Buermann et al., 2008; Prates-Clark et al., 2008; Saatchi et al., 2008; Cord and Rödder, 2011; Engler et al., 2013
Enhanced Vegetation Index (EVI)	MODIS	Morisette et al., 2006; Stohlgren et al., 2010; Cord and Rödder, 2011; Schmidt et al., 2013; Cord et al., 2014a,b
**Phenology**	MODIS, Landsat	Bradley and Mustard, 2006; Morisette et al., 2006; Tuanmu et al., 2010; Gonçalves et al., 2016
**Vegetation structure**		
Tree height	LiDAR	van Ewijk et al., 2014
Canopy roughness	QSCAT	Saatchi et al., 2008
**Other vegetation properties**		
Canopy moisture	Hyperspectral sensor, QSCAT	Buermann et al., 2008; Prates-Clark et al., 2008
Spectral heterogeneity/functional types	Hyperspectral sensor, Landsat	Morán-Ordóñez et al., 2012; Schmidt et al., 2013; Henderson et al., 2014; Pottier et al., 2014


In addition to abiotic properties of the environment, biotic characteristics also play an important role in shaping species’ spatial patterns (Wisz et al., 2013). RS can estimate many properties of the vegetated environment, and applications of products such as land-cover data or vegetation proxies to SDMs are on the rise (**Table 1**). Land cover has been considered as the primary determinant of species occurrences at a finer spatial resolution than climate (Pearson et al., 2004). Various studies (20% of 39 reviewed studies; **Table 1**) have applied land cover products derived from a variety of sensors (especially MODIS and Landsat) to SDMs. However, most of the current land cover information is in categorical format, which can lead to the propagation of classification errors (Cord and Rödder, 2011; Tuanmu and Jetz, 2014) and may not effectively represent the classes most relevant to the species of interest. In contrast, remotely sensed estimates of continuously varying ecosystem properties related to land cover and novel continuous land cover products can be used in SDMs and may avoid these limitations. Recent studies have found better performance from continuous estimates of vegetation properties and land cover rather than categorical representations (Wilson et al., 2013; Cord et al., 2014b; Tuanmu and Jetz, 2014). A range of remotely sensed measures of vegetation has been explored in SDMs, such as vegetation indices (Normalized difference vegetation index (NDVI), Enhanced Vegetation Index), phenology, and canopy moisture in order to evaluate variation in habitat quality at fine scales and in climatically homogenous regions (**Table 1**). Of vegetation metrics, NDVI, a useful measure of vegetation properties, has been extensively used as a predictor in SDMs (25.6%; **Table 1**). It represents photosynthetic activity and biomass in plants and is indirectly related to net primary production (Bradley and Fleishman, 2008). However, a study of Phillips et al. (2008) noted that while NDVI had high correlation with MODIS GPP (Gross primary production) and NPP (Net primary production), it was a less effective surrogate of productivity in areas of either sparse or dense vegetation. They found GPP to be better able to predict biogeographic patterns of species richness (Phillips et al., 2008), but we know of no studies that have used GPP in SDMs. Value-added science products, such as the MODIS primary productivity products, may provide more meaningful depictions of vegetation processes and improved environmental predictor variables for spatial models of biodiversity (Phillips et al., 2008).

In addition to the typical niche axes used to inform variable selection for SDMs of plant species, there is a large body of literature determining the ecosystem properties that influence invasibility of a system, and these can be used to guide applications of SDMs to evaluating invasion risk. Resource availability (e.g., light, CO_2,_ water, nutrients) often facilitates successful invasion. Invasibility is predicted to be greater in sites with more unused resources (Davis et al., 2000). By damaging the resident vegetation, disturbance reduces resource uptake and competition, increasing resource availability (Hobbs, 1989; D’Antonio, 1993). Therefore, invasions by invasive plant species are often associated with disturbance (e.g., Fox and Fox, 1986). However, distributions of invasive species are typically modeled using static environmental datasets that may poorly proxy these dynamic processes (Franklin, 2010b; Dormann et al., 2012). Temporal summaries of GPP may provide useful indicators. GPP estimates total ecosystem photosynthesis, the cumulative response of the vegetation to its environment, and may be used as a spatial proxy of resource ability. As well, the variability of GPP over time can reflect disturbance processes (Goetz et al., 2012). Hence, quantitative spatial measurements of GPP are expected to be relevant predictor variables for modeling invasibility. Also, including soil properties in SDMs may be useful as numerous studies have shown that soil properties, including nutrient availability, relate to invasibility (Huenneke et al., 1990; Burke, 1996; Harrison, 1999; Suding et al., 2004).

In this study, we hypothesize that the inclusion of recently developed global remotely sensed data products providing quantitative estimates of vegetation productivity and its dynamics, land cover, and soil properties, in addition to climatic layers, will enable a more complete representation of species’ ecological niches by SDMs. To test the hypothesis, bioclimatic data and RS data were used in isolated and combined models predicting the distribution of selected invasive plants across Southeast Asia (SEA).

Southeast Asia is an important region to global biodiversity; it has four of the world’s 25 biodiversity hotspots (Sodhi et al., 2004). However, much biodiversity is being lost (Peh, 2010) due to threatening processes such as habitat loss, degradation, climate change, and pollution (Pallewatta et al., 2003). In addition, and operating in synergy with these anthropogenic changes, invasive species damage the biodiversity and economy of the region (Peh, 2010; Gower et al., 2012; Nghiem et al., 2013). Although impacts of invasive species in SEA are apparent, research on the level and types of impacts caused by invasive species is still limited (Nghiem et al., 2013). There are also few applications of SDM methods, either for invasive species or in general, in the region. Among studies about species distributions worldwide, Porfirio et al. (2014) found only a small fraction were conducted in Asia (∼3%). The absence of research in this field is hindering SEA in providing a comprehensive assessment of invasive species (Peh, 2010; Gower et al., 2012), and in effectively managing this aspect of global environmental change.

The goal of this study is to provide an overview of potential invasibility to 14 priority invasive plants in SEA. To generalize estimates of invasion risk across species traits that may require different management approaches, we divided studied species into different life forms (herb, vine, and shrub). Such groupings based on life-history attributes have been widely used to understand the invasion process and propose tailored management strategies (McIntyre et al., 1995; Bear et al., 2006; Garrard et al., 2009). In addition, species were grouped by their origin status (native and non-native invasive species). Through evaluating SDMs by life forms and origin status, and using different environmental predictor variable sets, our study addresses the following questions:

(i) Which life forms of invasive plant species pose the greatest risk to SEA?
(ii) Are native weeds as great of a potential threat as non-native invasive species?
(iii) Do remotely sensed environmental predictor variables improve predictions of invasion risk over models constructed with climate variables alone?
(iv) Do the benefits of incorporating remotely sensed predictors in invasion risk models differ by species life form or by origin status?

## Materials and Methods

In order to evaluate the potential distributions of selected invasive plant species in SEA and to assess the contributions of remotely sensed environmental predictors to SDMs, we developed three model sets: models constructed along climate data only (CLIM), models with RS only (RS) and models with both climate and RS data (COMB). CLIM models used well-established bioclimatic datasets. The compiled RS predictor set covered a diverse range of surface parameters, namely topography, soil properties, global land cover, and vegetation productivity (GPP). Models used the Maximum Entropy (MaxEnt) algorithm. Model comparisons were based on the AUC score of model performance, average predicted areas, the level of spatial agreement in predicted distributions between model results, and the usage of RS and CLIM variables. The evaluation of invasion risk across life forms and origin status used predictions of suitable habitat area for individual species and predicted maps of invader richness. These datasets and methods are described in more detail below.

### Study Species and Occurrence Data

In this study, we modeled the potential distributions of 14 invasive species (**Table 2**) identified from the lists of native and non-native invasive species known in SEA (Matthews and Brand, 2004) and Vietnam (Ministry of Natural Resources and Environment and Ministry of Agriculture and Rural development, 2013).

**Table 2:** Description of the study species.

Family name	Common name	Scientific name	Life form	Origin	Median year of observations	Habitat
Asteraceae	Siam weed	*Chromolaena odorata*	Shrub	Non-native	2002	• Humid part of the inter-tropical zone, elevations below 2000 m
						• Open secondary habitats
	Whitetop Weed	*Parthenium hysterophorus*	Herb	Non-native	2005	• Humid and sub-humid tropics
						• Wide variety of soil types, more preferably in heavier fertile soils
						• Disturbed habitats (e.g., roadsides, railway tracks, river, and creek banks, buildings)
	Mile-a-Minute	*Mikania micrantha*	Vine	Non-native	2003	• Damp, lowland clearings, or open areas
						• Streams and roadsides, in or near forests, forest plantations, pastures, fence lines, tree crops
	Goat weed	*Ageratum conyzoides*	Herb	Non-native	1981	• Disturbed habitats, roadsides, degraded pasture and cultivated areas
Convolvulaceae	Bois	*Merremia boisiana*	Vine	Native	1956	• Forests; elevations of 100–1300 m^1^
Fabaceae	Giant sensitive plant	*Mimosa diplotricha*	Shrub	Non-native	2000	• Fertile areas; humid areas with available soil moisture
						• Open and disturbed habitats
	Catclaw mimosa	*Mimosa pigra*	Shrub	Non-native	1999	• Riparian areas and anthropogenic habitats (agricultural areas)
						• Disturbed and construction sites
	White leadtree	*Leucaena leucocephala*	Shrub/ Tree	Non-native	1990	• Open, often coastal habitats
						• Semi-natural and disturbed habitats
Poaceae	Buffel grass	*Cenchrus echinatus*	Grass	Non-native	1970	• Tropical regions
						• Dry and moist regions in rainfed areas and irrigated crops
						• Moderate moisture and light, sandy, well-drained soils at low elevations
	Bamboo grass	*Microstegium ciliatum*	Grass	Native	2000	• Along mesic roadsides, railroad right-of-way ditches, utility right-of-way, etc. Wetland, successional forest, planted forest, forest edges and margins, woodland borders
						• Not in areas with periodic standing water, nor in full, direct sunlight
Polygonaceae	Water hyacinth	*Eichhornia crassipes*	Herb	Non-native	1963	• Tropical and sub-tropical freshwater lakes and rivers, especially those enriched with plant nutrients, flooded rice
Tamaricaceae	Lantana	*Lantana camara*	Shrub	Non-native	1982	• Disturbed areas, pastures, roadsides and sometimes in native forests.
Leguminosae	Bauhinia	*Bauhinia touranensis*	Vine	Native	1957	• Open forests and thickets in valleys and on slopes; 500–1200 m^2^
	Kudzu	*Pueraria montana*	Vine	Native	1983	• Woods, plantation forests, open areas, abandoned fields
						• Wide variety of soil types but does not favor very wet soils
						• Wide geographic and climatic range


*^1^http://www.inaturalist.org/taxa/363279-Merremia-boisiana^2^http://www.efloras.org/florataxon.aspx?flora_id=3andtaxon_id=200011961 Origin and habitat preferred are classified according to Invasive Species Compendium developed by CABI (http://www.cabi.org/isc).*

Species occurrences were collected from the Global Biodiversity Information Facility^1^. Records were cleaned for obvious spatial errors (e.g., points that occurred in the ocean for terrestrial species) in ArcMap and duplicate records in the dataset were discarded (following Barik and Adhikari, 2011). All species modeled had more than ten occurrence records within the study area. The species occurrence records span lengthy collection periods. For each of the 14 species studied, the median years of the observations occurred in the period 1956–2005.

### Climate Data

Bioclimatic variables were obtained from the WorldClim database (Version 1.4), interpolated from measurements recorded during the period 1960 to 1990 from ∼46,000 climate stations worldwide (Hijmans et al., 2005). Eleven temperature and eight precipitation metrics, at 1 km resolution, were used, including annual means, seasonality, and extreme or limiting climatic conditions (**Table 3**). This dataset has been widely used for studies of plant species distributions (Pearson et al., 2007; Hernandez et al., 2008; Cord and Rödder, 2011; Zhu et al., 2017).

**Table 3:** Environmental variables.

Variables	Type of data	Source
Bedrock	Soil	Hengl et al., 2014
Bulk density	Soil	Hengl et al., 2014


Cation exchange capacity	Soil	Hengl et al., 2014


Soil texture fraction clay	Soil	Hengl et al., 2014


Coarse fragments volumetric	Soil	Hengl et al., 2014


Soil organic carbon stock	Soil	Hengl et al., 2014


Soil organic carbon content	Soil	Hengl et al., 2014


**Soil pH**	**Soil**	**Hengl et al., 2014**


Soil texture fraction silt	Soil	Hengl et al., 2014


Soil texture fraction sand	Soil	Hengl et al., 2014


**Evergreen/deciduous needle leaf trees**	**Land cover**	**Tuanmu and Jetz, 2014**


**Evergreen broadleaf trees**	**Land cover**	**Tuanmu and Jetz, 2014**


**Deciduous broadleaf trees**	**Land cover**	**Tuanmu and Jetz, 2014**


**Mixed/other trees**	**Land cover**	**Tuanmu and Jetz, 2014**


**Shrubs**	**Land cover**	**Tuanmu and Jetz, 2014**


**Herbaceous vegetation**	**Land cover**	**Tuanmu and Jetz, 2014**


**Cultivated and managed vegetation**	**Land cover**	**Tuanmu and Jetz, 2014**


**Regularly flooded vegetation**	**Land cover**	**Tuanmu and Jetz, 2014**


**Urban/built-up**	**Land cover**	**Tuanmu and Jetz, 2014**


Snow/ice	Land cover	Tuanmu and Jetz, 2014


**Barren**	**Land cover**	**Tuanmu and Jetz, 2014**


**Open water**	**Land cover**	**Tuanmu and Jetz, 2014**


**Gross primary productivity coefficient of variation (GPP_CV)**	**Vegetation productivity**	**Heinsch et al., 2003**


**Gross primary productivity (GPP_Mean)**	**Vegetation productivity**	**Heinsch et al., 2003**


Digital elevation model	Elevation	USGS, 1996


**Annual mean temperature**	**Climate**	**Hijmans et al., 2005**


**Mean diurnal temperature range**	**Climate**	**Hijmans et al., 2005**


**Isothermality**	**Climate**	**Hijmans et al., 2005**


Temperature seasonality	Climate	Hijmans et al., 2005


Max temperature of warmest month	Climate	Hijmans et al., 2005


Min temperature of coldest month	Climate	Hijmans et al., 2005


Temperature annual range	Climate	Hijmans et al., 2005


Mean temperature of wettest quarter	Climate	Hijmans et al., 2005


Mean temperature of driest quarter	Climate	Hijmans et al., 2005


Mean temperature of warmest quarter	Climate	Hijmans et al., 2005


Mean temperature of coldest quarter	Climate	Hijmans et al., 2005


**Annual precipitation**	**Climate**	**Hijmans et al., 2005**


**Precipitation of wettest month**	**Climate**	**Hijmans et al., 2005**


Precipitation of driest month	Climate	Hijmans et al., 2005


**Precipitation seasonality**	**Climate**	**Hijmans et al., 2005**


Precipitation of wettest quarter	Climate	Hijmans et al., 2005


Precipitation of driest quarter	Climate	Hijmans et al., 2005


**Precipitation of warmest quarter**	**Climate**	**Hijmans et al., 2005**


Precipitation of coldest quarter	Climate	Hijmans et al., 2005




*Bold text indicates the variables used as input for MaxEnt modeling.*

### Remote Sensing Data

A Digital Elevation Model (DEM) was derived from GTOPO30^2^ at 30 arc second resolution (approximately 1 km) (USGS, 1996). Ten soil layers representing soil physical and chemical properties (Hengl et al., 2014) (**Table 3**) at 1 km resolution were extracted from ftp://ftp.soilgrids.org/data/archive/12.Apr.2014/. This dataset was empirically developed from global compilations of publicly available soil profile data (ca. 110,000 soil profiles) and a selection of ∼75 global environmental covariates representing soil forming factors (mainly MODIS images, climate surfaces, Global Lithological Map, Harmonized World Soil Database and elevation) (Hengl et al., 2014).

We also included the consensus land cover layers developed by Tuanmu and Jetz (2014). They provide a continuous estimate of the probability of the occurrence of each of 12 land cover classes in each pixel, calculated from the agreements between four global land cover products. These estimates have been shown to have a greater ability to predict species distributions than the original categorical land cover products (Tuanmu and Jetz, 2014). These land cover data have a 1 km spatial resolution and are available online at http://www.earthenv.org/landcover. They represent consensus conditions incorporating estimates from the time period 1992–2006, but with greater weight to the later dates (Tuanmu and Jetz, 2014).

To quantify spatial and temporal variation in vegetation productivity, we used global annual MODIS17A3 (version 005) Gross primary productivity (GPP) data for 14 years (2001–2014) at 1 km resolution (Running et al., 2004). The Primary Production products are designed to provide an accurate regular measure of the yearly growth of the terrestrial vegetation (Heinsch et al., 2003). Data were downloaded from the Numerical Terradynamic Simulation Group (NTSG) at the University of Montana^3^. The mean and coefficient of variation of GPP (inter-annual variability) were calculated over the time series at each pixel and supplied to the SDMs.

All predictor variable layers were aligned to a common 1 km grid and projected in the Asia South Albers Equal Area Conic system using nearest neighbor resampling. Spatial environmental layers were pre-processed in the TerrSet software (Eastman, 2015).

### Selection of Environmental Predictions

To minimize predictor multicollinearity and its impact on subsequent analyses, we evaluated the inter-correlations among the 44 variables for all terrestrial pixels and retained a subset of uncorrelated (|*r*| < 0.75) predictor variables for species distribution modeling. Including too much flexibility may make it difficult for the model to distinguish noise from the true species response in real data sets (Baldwin, 2009; Merow et al., 2013). Minimizing correlation among variables, therefore, is assumed to increase the performance of species modeling (Austin, 2002). In this way, we reduced the number of predictors used per species to 7 climatic (out of 19) and 14 RS (out of 24) variables. All soil estimates were highly correlated across the study area, so only one was retained. See **Table 3** for the full list of initial variables, and those that were retained for modeling.

### Modeling Habitat Suitability of Species

To model habitat suitability, we used MaxEnt (version 3.3.3), a general-purpose machine learning method (Phillips et al., 2006). Among species distribution modeling techniques, MaxEnt is one of the most popular algorithms due to its predictive accuracy and ease of use (Elith et al., 2006; Phillips and Dudík, 2008). There are some characteristics that make MaxEnt highly suitable to modeling species distributions such as use of presence-only species data, flexibility in the handling of environmental data – including both continuous and categorical variables, and an ability to fit complex responses to the environmental variables (Phillips et al., 2006). Notably, MaxEnt is less sensitive to sample size, which makes MaxEnt a preferred predictive model across all sample sizes (Wisz et al., 2008).

In this study, we developed SDMs based only on the less-correlated climate and/or remotely sensed predictors with MaxEnt. To reduce overfitting, the regularization multiplier was set at 4. This parameter determines how strongly increases in model complexity are penalized during model optimization; higher values produce simpler models that are less overfit to the training data. Radosavljevic and Anderson (2014) found that regularization multiplier values from 2.00 to 4.00 were generally appropriate to minimize overfitting. For all 14 species, we created 10 random data partitions with 70% of the point localities assigned for training and 30% for testing and ran the three scenarios (see below) with each of these replicate partitions. Random samples of 10,000 background points were also used to develop each model.

MaxEnt model performance was evaluated using the area under the receiver operating characteristic curve (AUC) assessed on the withheld set of test points. AUC values range from 0 to 1. Values of 0.5 indicate that the model performs no better than expected by chance, while an AUC of 1 suggests perfect discriminatory abilities. Models with AUC > 0.7 are considered to achieve acceptable performance (Swets, 1988). Mean values, averaging across the 10 replicate runs and across species, of the resulting AUC distributions were used to compare the model scenarios run with different predictor sets. Continous MaxEnt outputs were converted to binary maps of habitat suitability using the tenth percentile training presence threshold (Escalante et al., 2013) in order to estimate the area of suitable habitat for each species predicted by each model. Variable usage by the models was determined with (1) a variable importance measure estimated as the decrease in model performance when a given variable was randomized, and (2) marginal variable response curves, which plot the predicted suitability for a species across the range of values for a given variable while all other variables are held at their mean values.

To test the contribution of RS data to modeling invasive species distributions, we ran MaxEnt with climate and satellite layers in separation and combination. Three scenarios were evaluated: MaxEnt runs with (1) climate data only (CLIM), these include the three temperature and four precipitation layers from the final reduced subset; (2) remote sensing data only (RS), with two GPP layers, one soil layer (pH) and eleven land cover classes from the reduced subset; and (3) climate and RS data combined (COMB) using all 21 layers of the reduced subset (see **Table 3**). The evaluation was based on (i) the AUC score; (ii) average predicted areas; (iii) % agreement in predicted distributions between model results; and (iv) differences in variable importance for the RS and CLIM variables. These comparisons were performed for all species overall, and when grouping by life forms and origin status. For the assessment of invasion risk, binary maps of habitat suitability for each species from the COMB model runs were used to determine the predicted habitat area and combined into maps of invader richness to compare the relative level of invasion risk among plant life forms and native/non-native invasive species.

## Results

### Model Performance

Overall, species distributions were generally predicted successfully. All species were successfully modeled (AUC > 0.7) by at least one predictor set (**Table 4**). Species with few occurrence records (less than 20), such as *Bauhinia touranensis*, *Mimosa pigra*, and *Merremia boisiana*, tended to be less successfully modeled in some of the model scenarios (AUC < 0.7). The remaining species with greater data availability achieved “good” (AUC > 0.8) to “excellent” (AUC > 0.9) performance (**Table 4**), according to the classification of Swets (1988).

**Table 4:** Variability (mean and standard devation) of species-specific AUC (area under the curve) scores, evaluated against the withheld test set of 30% of the presence records, for fourteen invasive weeds in 10 partition runs.

Species	Number of occurrences	CLIM	RS	COMB
*Ageratum conyzoides*	360	0.81 ± 0.01	0.74 ± 0.02	**0.84 ± 0.02**
*Bauhinia touranensis*	19	**0.85 ± 0.03**	0.51 ± 0.16	0.76 ± 0.07
*Cenchrus echinatus*	110	0.85 ± 0.04	**0.88 ± 0.03**	0.86 ± 0.04
*Chromolaena odorata*	167	0.88 ± 0.03	0.77 ± 0.03	**0.89 ± 0.03**
*Eichhornia crassipes*	81	0.65 ± 0.05	**0.84 ± 0.04**	0.84 ± 0.06
*Lantana camara*	162	**0.90 ± 0.02**	0.77 ± 0.04	0.88 ± 0.02
*Leucaena leucocephala*	192	0.85 ± 0.03	0.82 ± 0.02	**0.87 ± 0.03**
*Merremia boisiana*	13	**0.74 ± 0.08**	0.50 ± 0.10	0.72 ± 0.07
*Microstegium ciliatum*	96	**0.86 ± 0.03**	0.72 ± 0.06	0.86 ± 0.03
*Mikania micrantha*	171	0.92 ± 0.02	0.81 ± 0.04	**0.93 ± 0.02**
*Mimosa diplotricha*	54	**0.86 ± 0.06**	0.78 ± 0.07	0.85 ± 0.05
*Mimosa pigra*	19	**0.73 ± 0.09**	0.66 ± 0.06	0.64 ± 0.09
*Parthenium hysterophorus*	76	**0.97 ± 0.02**	0.85 ± 0.04	0.97 ± 0.01
*Pueraria montana*	417	**0.89 ± 0.02**	0.83 ± 0.02	0.84 ± 0.03
**Mean**		**0.84 ± 0.08**	0.75 ± 0.12	0.84 ± 0.08


*Three variable sets were used for each species. CLIM includes only bioclimatic predictors; RS includes only remote sensing predictors; COMB includes variables in CLIM and RS. AUC values for the best-performing model for each species are indicated in bold.*

**FIGURE 1:**
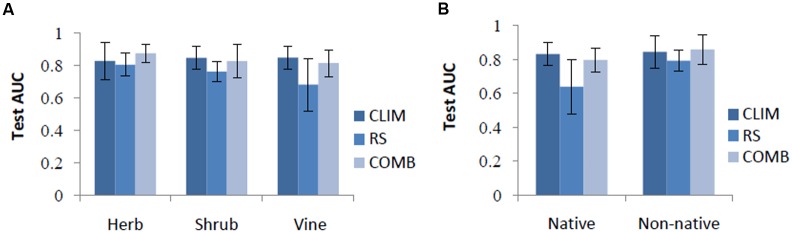
**Test AUC by life forms** **(A)** and by origin **(B)** among models. CLIM includes only bioclimatic predictors; RS includes only remote-sensing predictors; COMB includes variables in CLIM and RS. The error bars are standard deviations.

Across all species, the performance of the CLIM and COMB models was roughly equivalent (test AUC = 0.84 ± 0.08). Thus, along this metric alone, CLIM models may be preferable, as they are more parsimonious. On average, the RS models were the least successful (test AUC = 0.75 ± 0.12) (**Table 4**). However, the rankings differed somewhat for individual species and between species categories. CLIM models were preferred for 8 species, RS for 2, and COMB for the remaining 4 (**Table 4**). RS models were found to perform worst in predicting vine species (**Figure 1**) and native invasive species (**Figure 1**).

**FIGURE 2:**
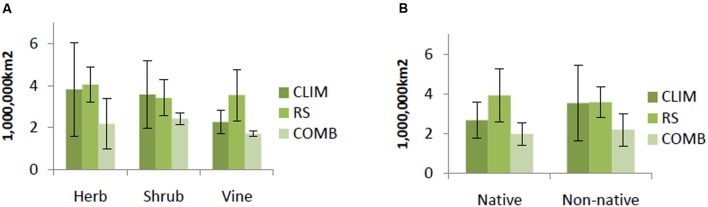
**Average predicted area by life forms** **(A)** and by origin **(B)** among models. Predicted value is identified based on 10% logistic threshold. CLIM includes only bioclimatic predictors; RS includes only remote-sensing predictors; COMB includes variables in CLIM and RS. The error bars are standard deviations.

COMB models generally predicted smaller areas of suitable habitat than either CLIM or RS models. This pattern was consistent across life forms and origin status, but strongest for herbs, shrubs, and non-native invasive species (**Figure 2**). CLIM and RS models tended to predict similar areas of suitable habitat, except for the case of vines and native invasive species. The RS models for these groups predicted larger areas of suitable habitat than did CLIM models (**Figure 2**).

**FIGURE 3:**
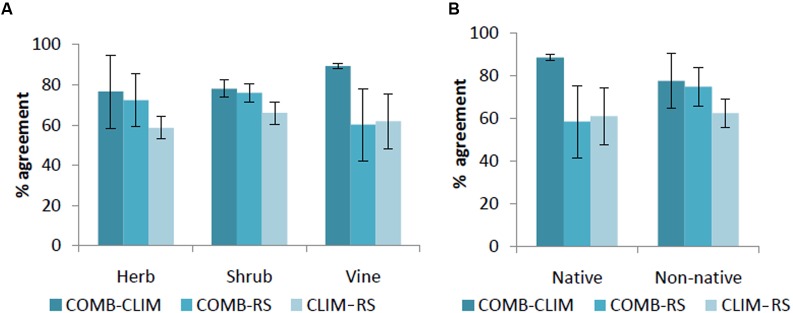
**Percentage of agreement in predicted area by life forms** **(A)** and by origin **(B)** among models. CLIM includes only bioclimatic predictors; RS includes only remote-sensing predictors; COMB includes variables in CLIM and RS. The error bars are standard deviations.

In general, spatial agreement in predicted habitat was greatest for pairwise comparisons with the COMB models (**Figure 3**). As an exception to this pattern, the agreement between COMB and RS was as low as between CLIM and RS for vines and native invasive species. At the individual species level (Supporting Information S2), COMB tended to be most similar to the individual model set (CLIM or RS) that performed better in the AUC evaluations (**Table 4**) – typically CLIM.

The average relative variable importance varied considerably among the predictors within the variable sets. In the CLIM set, mean diurnal temperature range (importance = 32.5% ± 22.0 and precipitation of warmest quarter (importance = 23.8% ± 17.4) were most important (**Table 5**). On average, other temperature variables (isothermality and annual mean temperature) have an importance around 12–13% and other variables contributed less than 10%. Of the variables in the RS predictor set, herbaceous vegetation land cover (importance = 16.7% ± 8.8) was the most important. Evergreen broadleaf tree, cultivated vegetation and GPP_CV were also important variables, with permutation importance ranging from 10 to 12% on average. In the COMB predictor set, the contribution of variables was similar to the CLIM and RS scenarios (**Table 5**). All variables had reduced importance in COMB than in either CLIM or RS, due to the inclusion of a larger number of variables in these models, but the rankings of variables within each predictor were generally consistent.

**Table 5:** Summary of the mean permutation importance (PI) of fourteen invasive plant species.

	COMB	CLIM	RS
	Mean ± *SD*	Mean ± *SD*	Mean ± *SD*
GPP_CV	2.1 ± 2.49		**10.76 ± 10.24**
GPP_Mean	2.83 ± 3.21		**8.41 ± 8.2**
Soil pH	1.32 ± 0.95		2.51 ± 5.34
Barren	1.21 ± 1.2		2.63 ± 2.09
Cultivated vegetation	3.83 ± 5.64		**11.22 ± 7.51**
Deciduous broad leaf trees	**5.17 ± 4.81**		**8.86 ± 8.25**
Evergreen broad leaf trees	**7.1 ± 9.11**		**12.37 ± 9.93**
Evergreen needle leaf trees	4.42 ± 9.24		6.19 ± 9.4
Herbaceous vegetation	**7.05 ± 7.38**		**16.71 ± 8.62**
Mixed trees	3.7 ± 4.99		**8.46 ± 6.18**
Open water	0.79 ±0.8		1.2 ± 0.77
Regular flooded vegetation	0.98 ± 1.6		2.53 ± 4.86
Shrubs	1.77 ± 1.46		6.56 ± 9.19
Urban	1.07 ± 1.19		1.6 ± 1.49
Annual mean temperature	4.32 ± 6.57	13.27 ± 14.57	
Mean diurnal temperature range	**17.65±16.04**	**32.48 ± 22.02**	
Isothermality	**7.72 ±6.84**	12.46 ± 10.98	
Annual precipitation	**7.53 ± 14.12**	9.06 ± 13.86	
Precipitation of wettest month	1.54 ± 1.94	3.26 ± 2.52	
Precipitation seasonality	3.67 ± 4.9	5.66 ± 6.56	
Precipitation of warmest quarter	**14.23 ± 9.93**	**23.81 ± 17.41**	


*SD is standard deviation. Mean values were calculated from the average of 14 species. Values in bold indicate variables with above-average importance in COMB (4.8%), CLIM (14.3%), and RS (7.1%).*

### Habitat Suitability

**FIGURE 4:**
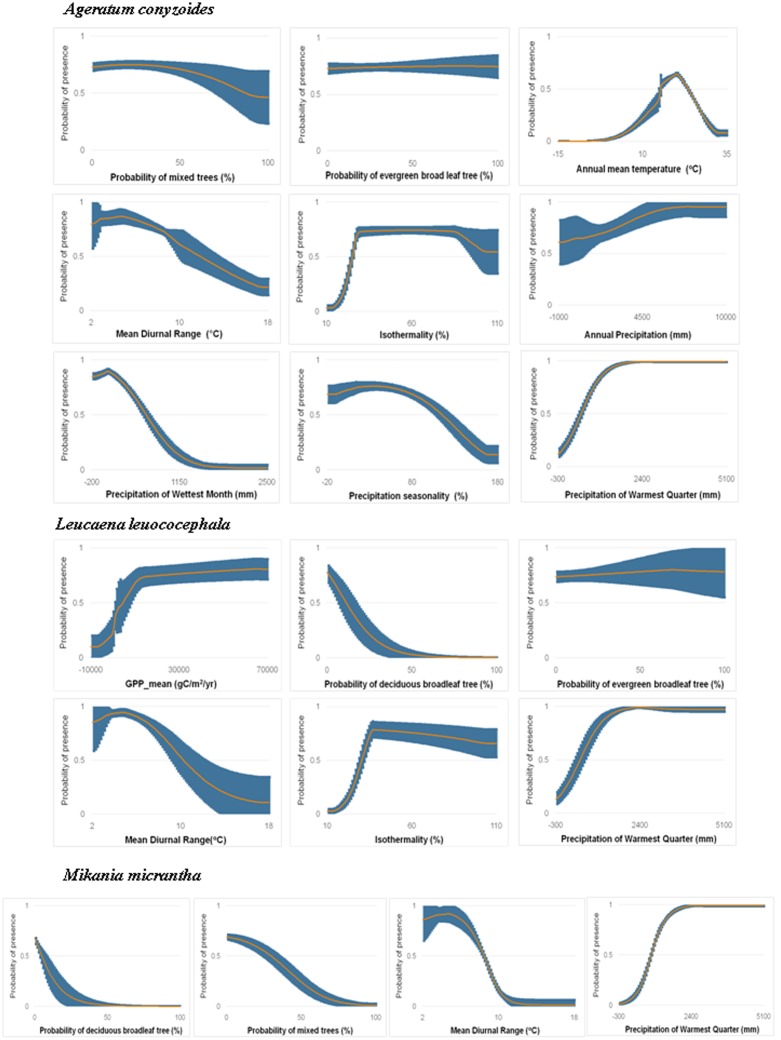
**Marginal response curves of *Ageratum conyzoides* (a non-native herb best modeled by COMB), *Leucaena leucocephala* (a non-native shrub best modeled by COMB) and *Mikania micrantha* (a non-native vine best modeled by COMB) for variables with importance >5% for each species in COMB models.** The orange curve in each plot is average response curve and the blue is standard deviation across all 10 partition runs. See other species in Supporting Information S1.

To assess the habitat suitability of species, we used results from COMB models. Response curves of each species (response curves are provided in **Figure 4** for a selected species of each life form that was best modeled by the COMB variable set, and for all species in Supporting Information S1) in COMB models reveal that, across species, sites were generally predicted to have high suitability (>0.6) in areas with low mean diurnal temperature range and moderate to high isothermality. The highest suitability (0.9–1) was also generally found in areas with high precipitation in the warmest season. Many modeled species (*Chromolaena odorata, Cenchrus echinatus*, *Eichhornia crassipes, Lantana camara, Mimosa diplotricha*) were not predicted to invade closed areas such as forests (negative responses to high canopy land-cover classes), although the aggressive vine *Pueraria montana* is a notable exception. In addition, for species models with important contributions from the productivity variables, suitability was generally found to be highest in environments with high GPP and low variability of GPP (Supporting Information S1).

**FIGURE 5:**
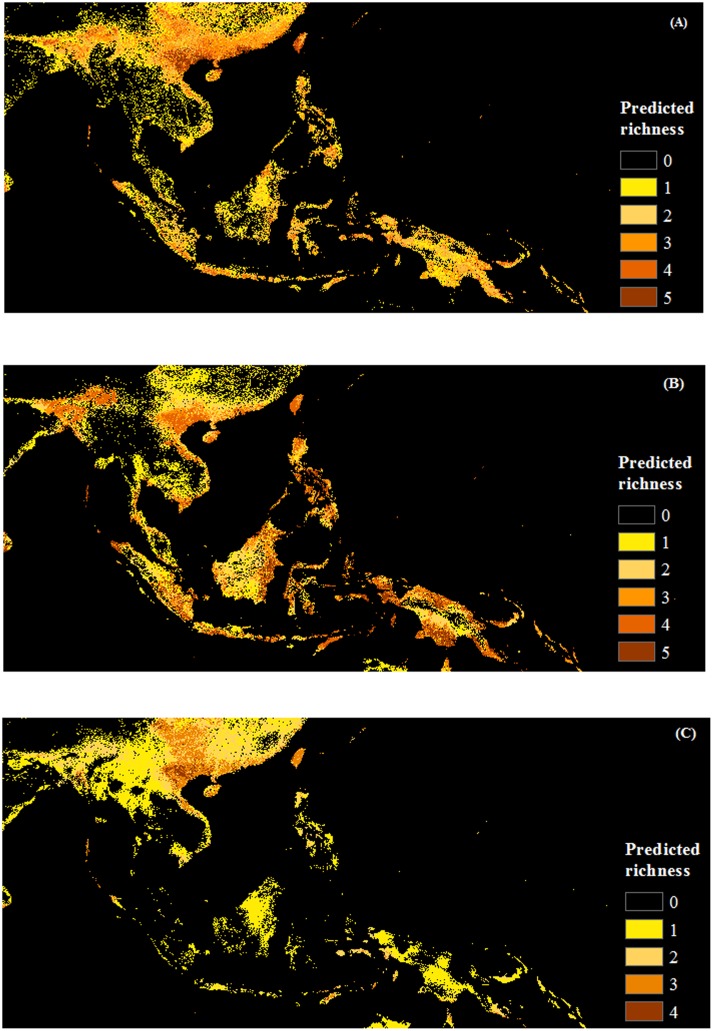
**Maps of predicted richness of invasive species by life form produced with COMB set (combing climate and remote sensing data).** **(A)** Herb, **(B)** Shrub and **(C)** Vine. The browner the color, the higher the predicted richness of invasive species.

Herb species receive the greatest area predicted to be at risk of invasion by one or more species (5.3 million km^2^, versus 4.9 million km^2^ and 4.3 million km^2^ for shrubs and vines, respectively), however, the area vulnerable to the greatest invader richness is fairly concentrated around the north and north center of Vietnam (**Figure 5**). Response curves of herb species (*Ageratum conyzoides, Cenchrus echinatus, Microstegium ciliatum*, and *Parthenium hysterophorus*) indicate they prefer high rainfall in the warmest quarter (more than >1500 mm), however, this variable was generally less important for herbs than it was for other life forms (Supporting Information S1). Additionally, herb species prefer habitat with diurnal temperature ranges less than 10°C and isothermality from 20 to 70%. Of the land cover variables, invasibility to herbs was more strongly related to the evergreen broadleaf and mixed forest classes, and to the cultivated class than were the other life forms. Response curves indicated that relationships with these cover classes were generally negative (Supporting Information S1).

Shrub species were predicted to have the greatest area at risk from multiple invaders: 1.3 million km^2^ were predicted to be suitable for four or more shrub species, as opposed to only 0.6 million km^2^ for herbs and 86 thousand km^2^ for vines (although note that only four vine species were modeled). Unlike the other life forms, regions suitable for multiple shrub invaders extended into countries in the south of the region such as Indonesia, Malaysia, and Philippines, as well as west to Bangladesh (**Figure 5**). Diurnal temperature range and precipitation of the warmest quarter were the most important factors for the distribution of these shrub species (e.g., *Chromolaena odorata, Lantana camara, Leucaena leucocephala*). Overall, models were more influenced by RS variables, especially land cover, for shrub species than for the other life forms. Shrubs exhibited generally negative associations with forested habitat (for all classes except the mixed forests) as well as with herbaceous land cover (Supporting Information S1).

In contrast to the other groups, large areas were predicted to be invasible to a single vine species. Areas vulnerable to greater richness of invasive vines were much more restricted, tending to occur in north and north-central Vietnam and Taiwan (**Figure 5**). While *Mikania micrantha* and *Pueraria montana* have less predicted area in SEA, *Bauhinia touranensis* and *Merremia boisiana* were predicted to invade much of the region (Supporting Information S2), especially in south China and north Vietnam. Unlike herbs and shrubs, distributions of vine species were generally unrelated to land cover (except for moderate influences of herbaceous land cover). Vine species received greater importance of climate factors, especially variables related to precipitation, than did the other life forms (Supporting Information S1).

**FIGURE 6:**
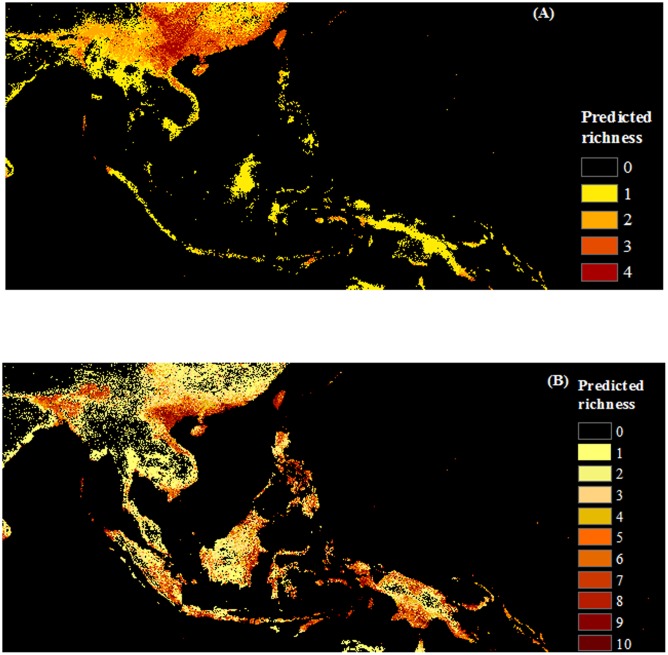
**Maps of predicted richness of invasive species by origin produced with COMB set (combing climate and remote sensing data).** **(A)** Native, **(B)** Non-native species. The browner color, the higher predicted richness of invasive species.

Results of average predicted area at the species level showed that as large areas are vulnerable to invasion by native as non-native invasive species (ca. 2 million km^2^) over the whole region (**Figure 2**). Cumulative levels of invasion risk are difficult to compare, since over twice as many non-native than native species were modeled, but substantial areas are at risk of invasion by one or more species of each origin status (6 million km^2^ and 4.3 million km^2^, for non-native and native invasive species, respectively). Native invasive species richness was mainly concentrated in the north and north center of Vietnam; non-native species had wider range of distribution and may potentially invade the whole region (**Figure 6**).

Comparing the total area predicted by the COMB models to be susceptible to the invasion of the 14 invasive species suggests which of the modeled species may be the greatest threats to the region. *Ageratum conyzoides, Eichhornia crassipes*, *Leucaeana leucocephala* and *Microstegium ciliatum* had the highest predicted area. *Lantana camara* and *Mimosa diplotricha* followed next. *Parthenium hysterophorus* had the lowest predicted area (Supporting Information S2).

## Discussion

### Model Performance

Quantitative comparisons of models with various predictor sets showed that models built with incorporation of RS and climatic data layers substantially reduced predicted areas across all life forms and origin status compared to models with climate and RS data alone (**Figure 2**). The mapped predictions for individual species reflect this pattern spatially (Supporting Information S2). Suitable habitat modeled with climate variables alone are quite smooth and generalized, while the inclusion of remotely sensed predictor variables adds more nuanced spatial detail to this overall pattern. The most widely used bioclimatic predictors, including those evaluated in this study, are derived from station data; interpolation introduces smoothing, producing generalized portrayals of environmental variability. As well, climate generally varies continuously over broad spatial scales. Thus, exclusively climate-based distribution models are unable to capture variations of species diversity at the landscape level (Saatchi et al., 2008). As a consequence, large areas of predicted suitability are often seen (Thuiller et al., 2004). In contrast, while the biotic niche axes estimated by RS can further inform distribution models and enable dynamic models, they are unable to replace climatic factors in identifying suitable habitat as bioclimatic conditions are still essential driving factors for species distributions (Thuiller et al., 2004; Cord and Rödder, 2011). The high percentage agreement of spatial predictions between models based on climatic predictors only and climatic and RS predictors found in this study, as well as the high variable importance scores given to climatic predictors in the combined models, also supports the indispensability of climate in shaping the distribution of invasive plant species. Similar studies have also found that using either climatic-derived or RS-derived predictors alone often leads to the overprediction of species distributions (Buermann et al., 2008; Saatchi et al., 2008; Cord and Rödder, 2011; Cord et al., 2014a). By incorporating complementary limiting environmental conditions, combined models of climatic and remotely sensed predictor variables reduce predicted areas, thereby refining modeled species distributions.

Although clearly refining the spatial patterns of predicted species distributions, in general, COMB models did not achieve higher accuracy than models with climate variables alone; RS models were often relatively poor. These results are in line with other studies (Zimmermann et al., 2007; Cord and Rödder, 2011; Cord et al., 2014a) that found that models based on RS data had the lowest AUC, compared to models with climate-derived predictors and climatic and RS predictors. Some explanations can be proposed for this. First, there may be temporal mismatch between occurrence data and environmental data. This is likely to be a more severe problem for remotely sensed predictors, which generally capture snapshots in time, rather than climatological averages, and which often describe environmental conditions, such as vegetation patterns, that vary over shorter time frames than does climate. Many of the occurrence records within museum or herbarium collections, comprising GBIF, are older; the land cover and vegetation productivity present at those sites at the time of the species’ presence may not be represented by remotely sensed current conditions. To test for this problem, we repeated our models with recent records only (collected after 1992). Removing older species records reduced model performance overall, likely due to the much smaller samples available to train the models. Remotely sensed predictors received slightly higher importance values in the COMB models than previously, but were still secondary to climatic variables (Supporting Information S3). Although temporal correspondence among species occurrences and environmental variables is a concern and should be considered in further studies, it does not seem to contribute to our conclusions.

Alternatively, the quality and information content of the RS products may influence model performance. The consensus land cover product was used in this study because it was expected to be more reliable than traditional global land cover datasets. Additionally, its continuous estimates of the probability of class presence may avoid errors associated with categorical data and provide some level of subpixel land cover information. However, it still has limitations related to the input datasets. Global land cover products are constrained to a relatively simple legend, with broad classes. The consensus product is further constrained to a simplified legend that harmonizes each of the input products. The generality of these classes may not capture regionally relevant differences and limit their usefulness to SDMs. The consensus land cover product is also limited by quality of the individual products it integrates (Tuanmu and Jetz, 2014). In land cover products, classification errors are not evenly distributed across space and classes (Strahler et al., 2006). For instance, lower accuracy for land cover classes of GlobCover products was found in some areas with limited data coverage (e.g., some areas in Amazonia) or in rugged terrain such as Laos (Bicheron et al., 2008). Also, cloud cover reduces the quality of the RS data, especially in tropical regions (Bradley and Fleishman, 2008).

**FIGURE 7:**
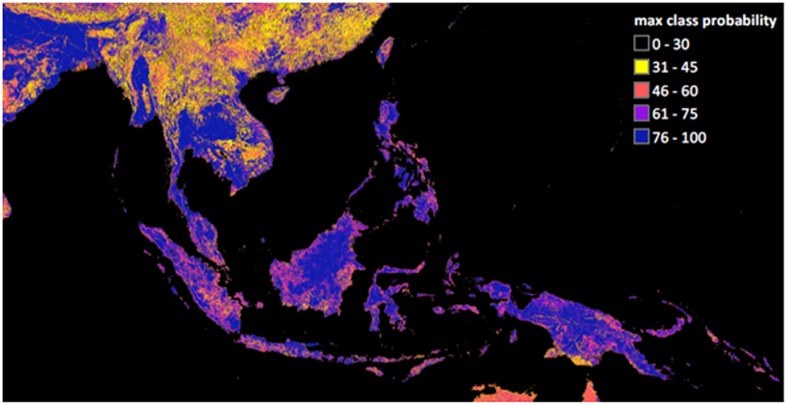
**Uncertainty in global land cover products revealed by the maximum class probability value, excluding the open water class, received in a pixel in the Consensus Land Cover dataset (Tuanmu and Jetz, 2014).** Low maximum probability values indicate a great deal of disagreement between individual land cover products.

Classification errors do seem to be contributing to the performance of RS variables in our study. Unexpectedly, species associations with land cover classes, when they were found to be important to models, were overwhelmingly negative. There is no ecological or logical reason for this. Instead, because the consensus land cover product estimates the certainty that a class is present, given the individual land cover datasets, this suggests that habitat suitability tends to be greatest for the modeled species in areas with high land cover uncertainty. Such uncertainty may be due to inadequacies in the class definitions in this region, fine-scaled mosaics of land cover classes within a 1 km pixel, or simply poor classification performance. Indeed, using the maximum estimated probability of class membership as an indicator of certainty supports this interpretation. Large areas of SEA, including many of the same locations with high-predicted invasibility, exhibit low certainty of the land cover information (**Figure 7**). Further work is necessary to validate the consensus land cover products in SEA and, especially, to determine the meaning of areas with great class uncertainty. This is troubling and argues against the use of global land cover products in SDMs. Quantitative remotely sensed estimates of ecosystem structure and function may overcome some of the problems of categorical datasets, and we strongly advocate for their expanded use and continued evaluation in SDM contexts. Interestingly, the quantitative measures of vegetation productivity used in this study, while making important contributions to the RS model set, generally dropped out of the COMB models. This may be because of interdependencies between climate variables and the photosynthetic efficiency term used in the MODIS GPP product, which relies on both temperature and moisture (Running et al., 2004), and thus would not be detected by the simple univariate correlation analysis used to screen input variables.

Another limitation to model performance in this study is the sample size of the species occurrence records. Performance of SDMs in the study varied among species. Species with few occurrence records occurring in a wide range of habitats, such as *Mimosa pigra*, have lower performance than others. This is because SDMs perform better with larger sample sizes and for species occupying a narrow environmental niche than for generalist species (Hernandez et al., 2006). Although *Mimosa pigra* has been recorded as one of the most invasive plants in many countries in SEA (Thi, 2000; MacKinnon, 2002; Vanna and Nang, 2005; Nghiem et al., 2013), the number of occurrence records of this species in SEA is still limited. This reflects lack of research and awareness of the public and government for invasive species detection in the region, which should be more encouraged. Also, using hyperspectral RS to detect invasive species occurrences (Andrew and Ustin, 2008; Hestir et al., 2008) can be a solution for developing high-quality, unbiased occurrence data inputs (He et al., 2015), and also may reduce temporal mismatch between species occurrences and environmental variables. In addition to model development, sample size influences model evaluation. Performance measures such as the AUC provide a single spatial summary value. AUC has been criticized for its inability to convey information about the spatial pattern of predictions or uncertainty (Franklin, 2010a). Yet spatial variation can be considerable. Because AUC is often calculated from a tiny proportion of the pixels modeled, wildly different spatial predictions can receive similar, and indeed very high, AUC estimates (Synes and Osborne, 2011). For this reason, we prefer to present a suite of evaluation tools, including total predicted area and estimates of spatial agreement, in addition to the AUC.

### Habitat Suitability

Both non-native and native invasive species were predicted to occur across large areas of SEA, and thus may pose similar risk to the region. Among life forms, shrub species potentially pose greater risk because of the predictions of high shrub invader richness over large areas, based on the set of species assessed. Most countries in the region have suitable habitat for these species. In general, shrubs exhibited weaker environmental associations than the other life forms (as seen in the lower variable importance scores), suggesting they may be tolerant of a broader range of conditions. Relative to shrub and herb species, vine species’ distributions were most strongly driven by climatic factors. This may facilitate their spread under climate change. Invasive species may disproportionately benefit from global climate change (Dukes and Mooney, 1999), and vines may be a good example of these concerns. Climate projections for the region include increases in annual temperature and in summertime precipitation (Christensen et al., 2007), the latter variable was important to nearly all vine species distributions, all of which showed positive associations. Without strong controls by biotic factors such as land cover, vines may invade valuable evergreen broadleaf trees forests in SEA. A native vine, *Merremia boisiana* is an example. In the past decade, the vine has spread dramatically over South China (Wang et al., 2005; Wu et al., 2007) and the north and center of Vietnam (Le et al., 2012) and our results reveal that more than 1.6 million km^2^ are invasible to this species, largely concentrated in China and Vietnam. These findings suggest that awareness of invasive species and prevention and eradication efforts should not overlook the life form or origin status of the species of concern.

Interestingly, in contrast to our expectations, we found that for some species (*Microstegium ciliatum* and *Mimosa diplotricha*) suitability was negatively related to the variability of GPP (GPP_CV), which was used to proxy disturbance processes. This suggests that invasion is possible even with low disturbance, contradicting knowledge summarized by Lozon and MacIsaac (1997) that the establishment and spread of invasive plants are associated with disturbance. Although disturbance is certainly a factor in many invasions, an over-generalization that invasion requires disturbance can lead to low awareness of invasion in intact areas. Further field-based studies about invasibility of these species under difference disturbance levels should be conducted. The effectiveness of GPP variability as an indicator of diverse disturbance processes and diverse ecosystems should also be evaluated. The relatively short duration of the satellite archive from which it was computed is certainly a limitation.

Given that many of the study species were identified from Vietnam’s invasive weed list, it is not surprising that we found, within the region, north and north central Vietnam were most susceptible to the invasion of weeds (**Figures 5**, **6**). However, it is worth emphasizing that many of the invasive weeds predicted in this region also have high invasibility in China, where outbreaks have been recorded (Yan et al., 2001). Biological invasions are a trans-border issue. Similarly, provinces (Guangxi, Quangdong, and Yunnan) sharing borders with Vietnam, Lao, and Myanmar are listed as areas with a high number of invasive species in China (Xu et al., 2012). Effective management requires that invasions be considered in the context of the region (SEA), rather than a country (Paini et al., 2010). Studies such as ours can help the Vietnamese and other governments to prioritize management actions for invasive species within the country and also to inform biosecurity policy across borders.

## Conclusion

This study demonstrated that although the environmental attributes derived from RS data did not strongly improve the accuracy of SDM predictions, they did provide more landscape-level detail that refined species distribution predictions in space. Therefore, the inclusion of remotely sensed variables in SDMs likely is worthwhile. Furthermore, our results highlight shortcomings of land cover products, which are widely used in SDMs. There are widespread uncertainties in global land cover products and, disconcertingly, those sites with the greatest uncertainty also seem to be consistently ecologically important to the modeled species. We caution against continued use of land cover information in SDMs, which may propagate errors and confound interpretation. Greater adoption of quantitative remotely sensed datasets estimating ecosystem structure and function may mitigate the weaknesses and limited utility of RS observed in this study. From the standpoint of biodiversity management, our findings have implications in targeting management to susceptible areas, providing initial data for invasive species risk assessments, and proposing biosecurity policy in the region.

## Author Contributions

TT prepared input data, performed models and interpreted results, wrote manuscript and acted as corresponding author. MA supervised development of work, provided guidance throughout the project, and edited manuscript. GH contributed to editing manuscript.

## Conflict of Interest Statement

The authors declare that the research was conducted in the absence of any commercial or financial relationships that could be construed as a potential conflict of interest.

## Footnotes

1 http://www.gbif.org/

2 http://earthexplorer.usgs.gov/

3 http://www.ntsg.umt.edu/project/mod17

## References

[B1] Andrew M. E. Ustin S. L. (2008). The role of environmental context in mapping invasive plants with hyperspectral image data. *Remote Sens. Environ.* 112 4301–4317. 10.1016/j.rse.2008.07.016

[B2] Andrew M. E. Ustin S. L. (2009). Habitat suitability modelling of an invasive plant with advanced remote sensing data. *Divers. Distrib.* 15 627–640. 10.1111/j.1472-4642.2009.00568.x

[B3] Andrew M. E. Wulder M. A. Nelson T. A. (2014). Potential contributions of remote sensing to ecosystem service assessments. *Progr. Phys. Geogr.* 38 328–353. 10.1177/0309133314528942

[B4] Austin M. P. (2002). Spatial prediction of species distribution: an interface between ecological theory and statistical modelling. *Ecol. Model.* 157 101–118. 10.1016/S0304-3800(02)00205-3

[B5] Avril L. Kelty M. J. (1999). Establishment and control of hay-scented fern: a native invasive species. *Biol. Invasions* 1 223–236. 10.1023/A:1010098316832

[B6] Baldwin R. A. (2009). Use of maximum entropy modeling in wildlife research. *Entropy* 11 854–866. 10.3390/e11040854

[B7] Barik S. Adhikari D. (2011). “Predicting geographic distribution of an invasive species (*Chromolaena odorata* L. (King) & H. E. Robins) in the Indian subcontinent under climate change scenarios,” in *Invasive Alien Plants—An Ecological Appraisal for the Indian Sub-continent*, eds Bhatt J. R. Singh J. S. Singh S. P. Tripathi R. S. Kohli R. K. (Wallingford: CABI), 77–88. 10.1079/9781845939076.0077

[B8] Bear R. Hill W. Pickering C. M. (2006). Distribution and diversity of exotic plant species in montane to alpine areas of Kosciuszko National Park. *Cunninghamia* 9 559–570.

[B9] Bicheron P. Defourny P. Brockmann C. Schouten L. I. Vancutsem C. Huc M. (2008). *GLOBCOVER: Products Description and Validation Report.* Toulouse: MEDIAS France.

[B10] Bradley B. A. Fleishman E. (2008). Can remote sensing of land cover improve species distribution modelling? *J. Biogeogr.* 35 1158–1159. 10.1111/j.1365-2699.2008.01928.x

[B11] Bradley B. A. Mustard J. F. (2006). Characterizing the landscape dynamics of an invasive plant and risk of invasion using remote sensing. *Ecol. Appl.* 16 1132–1147. 10.1890/1051-0761(2006)016[1132:CTLDOA]2.0.CO;2 16827008

[B12] Bradley B. A. Olsson A. D. Wang O. Dickson B. G. Pelech L. Sesnie S. E. (2012). Species detection vs. habitat suitability: are we biasing habitat suitability models with remotely sensed data? *Ecol. Model.* 244 57–64. 10.1016/j.ecolmodel.2012.06.019

[B13] Buermann W. Saatchi S. Smith T. B. Zutta B. R. Chaves J. A. Milá B. (2008). Predicting species distributions across the Amazonian and Andean regions using remote sensing data. *J. Biogeogr.* 35 1160–1176. 10.1111/j.1365-2699.2007.01858.x

[B14] Burke M. J. W. (1996). An experimental study of plant community invasibility. *Ecology* 77 776–790. 10.2307/2265501

[B15] Campos V. E. Cappa F. M. Viviana F. M. Giannoni S. M. (2016). Using remotely sensed data to model suitable habitats for tree species in a desert environment. *J. Veg. Sci.* 27 200–210. 10.1111/jvs.12328

[B16] Christensen J. H. Hewitson B. Busuioc A. Chen A. Gao X. Held I. (2007). “Regional climate projections,” in *Climate Change 2007: The Physical Science Basis. Contribution of Working Group I to the Fourth Assessment Report of the Intergovernmental Panel on Climate Change*, eds Solomon S. Qin D. Manning M. Chen Z. Marquis M. Averyt K. B. (Cambridge: Cambridge University Press).

[B17] Cord A. Rödder D. (2011). Inclusion of habitat availability in species distribution models through multi-temporal remote-sensing data? *Ecol. Appl.* 21 3285–3298. 10.1890/11-0114.1

[B18] Cord A. F. Klein D. Gernandt D. S. la Rosa J. A. P. Dech S. (2014a). Remote sensing data can improve predictions of species richness by stacked species distribution models: a case study for Mexican pines. *J. Biogeogr.* 41 736–748. 10.1111/jbi.12225

[B19] Cord A. F. Klein D. Mora F. Dech S. (2014b). Comparing the suitability of classified land cover data and remote sensing variables for modeling distribution patterns of plants. *Ecol. Model.* 272 129–140. 10.1016/j.ecolmodel.2013.09.011

[B20] D’Antonio C. M. (1993). Mechanisms controlling invasion of coastal plant communities by the alien succulent *Carpobrotus edulis*. *Ecology* 74 83–95. 10.2307/1939503

[B21] Davis M. A. Grime J. P. Thompson K. (2000). Fluctuating resources in plant communities: a general theory of invasibility. *J. Ecol.* 88 528–534. 10.1046/j.1365-2745.2000.00473.x

[B22] Deblauwe V. Droissart V. Bose R. Sonké B. Blach-Overgaard A. Svenning J. C. (2016). Remotely sensed temperature and precipitation data improve species distribution modelling in the tropics. *Glob. Ecol. Biogeogr.* 25 443–454. 10.1111/geb.12426

[B23] Dormann C. F. Schymanski S. J. Cabral J. Chuine I. Graham C. Hartig F. (2012). Correlation and process in species distribution models: bridging a dichotomy. *J. Biogeogr.* 39 2119–2131. 10.1111/j.1365-2699.2011.02659.x

[B24] Dukes J. S. Mooney H. A. (1999). Does global change increase the success of biological invaders? *Trends Ecol. Evol.* 14 135–139. 10322518 10.1016/s0169-5347(98)01554-7

[B25] Eastman J. (2015). *TerrSet: Geospatial Monitoring and Modeling Software.* Worcester, MA: Clark University.

[B26] Elith J. Graham C. H. Anderson R. P. Dudík M. Ferrier S. Guisan A. (2006). Novel methods improve prediction of species’ distributions from occurrence data. *Ecography* 29 129–151. 10.1111/j.2006.0906-7590.04596.x

[B27] Engler R. Waser L. T. Zimmermann N. E. Schaub M. Berdos S. Ginzler C. (2013). Combining ensemble modeling and remote sensing for mapping individual tree species at high spatial resolution. *For. Ecol. Manag.* 310 64–73. 10.1016/j.foreco.2013.07.059

[B28] Escalante T. Rodríguez-Tapia G. Linaje M. Illoldi-Rangel P. González-López R. (2013). Identification of areas of endemism from species distribution models: threshold selection and Nearctic mammals. *TIP* 16 5–17. 10.1016/S1405-888X(13)72073-4

[B29] Estes L. Okin G. Mwangi A. Shugart H. (2008). Habitat selection by a rare forest antelope: a multi-scale approach combining field data and imagery from three sensors. *Remote Sens. Environ.* 112 2033–2050. 10.1016/j.rse.2008.01.004

[B30] Evangelista P. Stohlgren T. Morisette J. Kumar S. (2009). Mapping invasive tamarisk (Tamarix): a comparison of single-scene and time-series analyses of remotely sensed data. *Remote Sens.* 1 519–533. 10.3390/rs1030519

[B31] Feilhauer H. He K. S. Rocchini D. (2012). Modeling species distribution using niche-based proxies derived from composite bioclimatic variables and MODIS NDVI. *Remote Sens.* 4:2057 10.3390/rs4072057

[B32] Fernández M. Hamilton H. Alvarez O. Guo Q. (2012). Does adding multi-scale climatic variability improve our capacity to explain niche transferability in invasive species? *Ecol. Model.* 246 60–67. 10.1016/j.ecolmodel.2012.07.025

[B33] Fox M. D. Fox B. J. (1986). “The susceptibility of natural communities to invasion,” in *Ecology of Biological Invasions*, eds Groves R. H. Burdon J. J. (Cambridge: Cambridge University Press), 57–66.

[B34] Franklin J. (2010a). *Mapping Species Distributions: Spatial Inference and Prediction.* Cambridge: Cambridge University Press 10.1017/CBO9780511810602

[B35] Franklin J. (2010b). Moving beyond static species distribution models in support of conservation biogeography. *Divers. Distrib.* 16 321–330. 10.1111/j.1472-4642.2010.00641.x

[B36] Garrard G. Bekessy S. Wintle B. (2009). *Determining Necessary Survey Effort to Detect Invasive Weeds in Native Vegetation Communities.* Final Report ACERA Project No. 0906 Parkville, VIC: University of Melbourne.

[B37] Genovesi P. (2005). Eradications of invasive alien species in Europe: a review. *Biol. Invasions* 7 127–133. 10.1007/s10530-004-9642-9

[B38] Goetz S. J. Bond-Lamberty B. Law B. E. Hicke J. A. Huang C. Houghton R. A. (2012). Observations and assessment of forest carbon dynamics following disturbance in North America. *J. Geophys. Res. Biogeosci.* 117 G02022 10.1029/2011jg001733

[B39] Gonçalves J. Alves P. Pôças I. Marcos B. Sousa-Silva R. Lomba Â. (2016). Exploring the spatiotemporal dynamics of habitat suitability to improve conservation management of a vulnerable plant species. *Biodiver. Conserv.* 25 2867–2888. 10.1007/s10531-016-1206-7

[B40] Gower D. Johnson K. Richardson J. Rosen B. Rüber L. Williams S. (2012). *Biotic Evolution and Environmental Change in Southeast Asia.* Cambridge: Cambridge University Press 10.1017/CBO9780511735882

[B41] Groves R. Hosking J. Batianoff G. Cooke D. Cowie I. Johnson R. (2003). *Weed Categories for Natural and Agricultural Ecosystem Management.* Canberra, ACT: Bureau of Rural Sciences.

[B42] Guisan A. Thuiller W. (2005). Predicting species distribution: offering more than simple habitat models. *Ecol. Lett.* 8 993–1009. 10.1111/j.1461-0248.2005.00792.x 34517687

[B43] Guisan A. Zimmermann N. E. (2000). Predictive habitat distribution models in ecology. *Ecol. Model.* 135 147–186. 10.1016/S0304-3800(00)00354-9

[B44] Harrison S. (1999). Native and alien species diversity at the local and regional scales in a grazed California grassland. *Oecologia* 121 99–106. 10.1007/s004420050910 28307892

[B45] He K. S. Bradley B. A. Cord A. F. Rocchini D. Tuanmu M. N. Schmidtlein S. (2015). Will remote sensing shape the next generation of species distribution models? *Remote Sens. Ecol. Conserv.* 1 4–18. 10.1002/rse2.7

[B46] Heinsch F. A. Reeves M. Votava P. Kang S. Milesi C. Zhao M. (2003). *GPP and NPP (MOD17A2/A3) Products NASA MODIS Land Algorithm.* Missoula, MT: University of Montana, 1–57.

[B47] Henderson E. B. Ohmann J. L. Gregory M. J. Roberts H. M. Zald H. (2014). Species distribution modelling for plant communities: stacked single species or multivariate modelling approaches? *Appl. Veget. Sci.* 17 516–527. 10.1111/avsc.12085

[B48] Hengl T. de Jesus J. M. MacMillan R. A. Batjes N. H. Heuvelink G. B. M. Ribeiro E. (2014). SoilGrids1km — Global soil information based on automated mapping. *PLoS ONE* 9:e105992 10.1371/journal.pone.0105992 PMC4149475 25171179

[B49] Hernandez P. A. Franke I. Herzog S. K. Pacheco V. Paniagua L. Quintana H. L. (2008). Predicting species distributions in poorly-studied landscapes. *Biodivers. Conserv.* 17 1353–1366. 10.1007/s10531-007-9314-z

[B50] Hernandez P. A. Graham C. H. Master L. L. Albert D. L. (2006). The effect of sample size and species characteristics on performance of different species distribution modeling methods. *Ecography* 29 773–785. 10.1111/j.0906-7590.2006.04700.x

[B51] Hestir E. L. Khanna S. Andrew M. E. Santos M. J. Viers J. H. Greenberg J. A. (2008). Identification of invasive vegetation using hyperspectral remote sensing in the California Delta ecosystem. *Remote Sens. Environ.* 112 4034–4047. 10.1016/j.rse.2008.01.022

[B52] Hijmans R. J. Cameron S. E. Parra J. L. Jones P. G. Jarvis A. (2005). Very high resolution interpolated climate surfaces for global land areas. *Int. J. Climatol.* 25 1965–1978. 10.1002/joc.1276

[B53] Hobbs R. J. (1989). “The nature and effects of disturbance relative to invasions,” in *Biological Invasions: A Global Perspective*, eds Drake J. A. Mooney H. A. (New York, NY: John Wiley and Sons Ltd), 389–405.

[B54] Hoffman J. D. Narumalani S. Mishra D. R. Merani P. Wilson R. G. (2008). Predicting potential occurrence and spread of invasive plant species along the North Platte River, Nebraska. *Invasive Plant Sci. Manag.* 1 359–367. 10.1614/IPSM-07-048.1

[B55] Hooftman D. A. P. Oostermeijer J. G. B. den Nijs J. C. M. (2006). Invasive behaviour of *Lactuca serriola* (Asteraceae) in the Netherlands: spatial distribution and ecological amplitude. *Basic Appl. Ecol.* 7 507–519. 10.1016/j.baae.2005.12.006

[B56] Huenneke L. F. Hamburg S. P. Koide R. Mooney H. A. Vitousek P. M. (1990). Effects of soil resources on plant invasion and community structure in Californian serpentine grassland. *Ecology* 71 478–491. 10.2307/1940302

[B57] Kearney M. Porter W. (2009). Mechanistic niche modelling: combining physiological and spatial data to predict species’ ranges. *Ecol. Lett.* 12 334–350. 10.1111/j.1461-0248.2008.01277.x 19292794

[B58] Kulhanek S. A. Leung B. Ricciardi A. (2011). Using ecological niche models to predict the abundance and impact of invasive species: application to the common carp. *Ecol. Appl.* 21 203–213. 10.1890/09-1639.1 21516898

[B59] Le B. Nguyen T. Adkins S. (2012). Damage caused by *Merremia eberhardtii* and *Merremia boisiana* to biodiversity of Da Nang City, Vietnam. *Pak. J. Weed Sci. Res.* 18 895–905.

[B60] Lozon J. D. MacIsaac H. J. (1997). Biological invasions: are they dependent on disturbance? *Environ. Rev.* 5 131–144. 10.1139/a97-007

[B61] MacKinnon J. R. (2002). Invasive alien species in Southeast Asia. *Asean Biodivers.* 2 9–11.

[B62] Matthews S. Brand K. (2004). *Tropical Asia Invaded: The Growing Danger of Invasive Alien Species.* Nairobi: GISP Secretariat.

[B63] McIntyre S. Lavorel S. Tremont R. (1995). Plant life-history attributes: their relationship to disturbance response in herbaceous vegetation. *J. Ecol.* 83 31–44. 10.2307/2261148

[B64] Merow C. Smith M. J. Silander J. A. (2013). A practical guide to MaxEnt for modeling species’ distributions: what it does, and why inputs and settings matter. *Ecography* 36 1058–1069. 10.1111/j.1600-0587.2013.07872.x

[B65] Ministry of Natural Resources and Environment and Ministry of Agriculture and Rural development (2013). *Inter-Ministerial Circulation No. 27/TTLT-BTNMT-BNNPTNT*. Vientiane: Ministry of Natural Resources and Environment.

[B66] Morán-Ordóñez A. Suárez-Seoane S. Elith J. Calvo L. de Luis E. (2012). Satellite surface reflectance improves habitat distribution mapping: a case study on heath and shrub formations in the Cantabrian Mountains (NW Spain). *Divers. Distrib.* 18 588–602. 10.1111/j.1472-4642.2011.00855.x

[B67] Morisette J. T. Jarnevich C. S. Ullah A. Cai W. Pedelty J. A. Gentle J. E. (2006). A tamarisk habitat suitability map for the continental United States. *Front. Ecol. Environ.* 4 11–17. 10.1890/1540-9295(2006)004[0012:athsmf]2.0.co;2

[B68] Nghiem L. T. P. Soliman T. Yeo D. C. J. Tan H. T. W. Evans T. A. Mumford J. D. (2013). Economic and environmental impacts of harmful non-indigenous species in Southeast Asia. *PLoS ONE* 8:e71255 10.1371/journal.pone.0071255 PMC3739798 23951120

[B69] Paini D. R. Worner S. P. Cook D. C. De Barro P. J. Thomas M. B. (2010). Threat of invasive pests from within national borders. *Nat. Commun.* 1:115 10.1038/ncomms1118 21081913

[B70] Pallewatta N. Reaser J. Gutierrez A. (2003). “Prevention and management of invasive alien species,” in *Proceedings of a Workshop on Forging Cooperation throughout South and Southeast Asia* (Cape Town: Global Invasive Species Programme).

[B71] Parviainen M. Luoto M. Ryttari T. Heikkinen R. K. (2008). Modelling the occurrence of threatened plant species in taiga landscapes: methodological and ecological perspectives. *J. Biogeogr.* 35 1888–1905. 10.1111/j.1365-2699.2008.01922.x

[B72] Parviainen M. Zimmermann N. E. Heikkinen R. K. Luoto M. (2013). Using unclassified continuous remote sensing data to improve distribution models of red-listed plant species. *Biodivers. Conserv.* 22 1731–1754. 10.1007/s10531-013-0509-1

[B73] Pau S. Edwards E. J. Still C. J. (2013). Improving our understanding of environmental controls on the distribution of C3 and C4 grasses. *Glob. Change Biol.* 19 184–196. 10.1111/gcb.12037 23504730

[B74] Pearson R. G. (2010). Species’ distribution modeling for conservation educators and practitioners. *Lessons Conserv.* 3 54–89.

[B75] Pearson R. G. Dawson T. P. (2003). Predicting the impacts of climate change on the distribution of species: are bioclimate envelope models useful? *Glob. Ecol. Biogeogr.* 12 361–371. 10.1046/j.1466-822X.2003.00042.x

[B76] Pearson R. G. Dawson T. P. Liu C. (2004). Modelling species distributions in Britain: a hierarchical integration of climate and land-cover data. *Ecography* 27 285–298. 10.1111/j.0906-7590.2004.03740.x

[B77] Pearson R. G. Raxworthy C. J. Nakamura M. Peterson A. T. (2007). Predicting species distributions from small numbers of occurrence records: a test case using cryptic geckos in Madagascar. *J. Biogeogr.* 34 102–117. 10.1111/j.1365-2699.2006.01594.x

[B78] Peh K. S. H. (2010). Invasive species in Southeast Asia: the knowledge so far. *Biodivers. Conserv.* 19 1083–1099. 10.1007/s10531-009-9755-7

[B79] Peterson A. T. (2003). Predicting the geography of species’ invasions via ecological niche modeling. *Q. Rev. Biol.* 78 419–433. 10.1086/378926 14737826

[B80] Peterson A. T. (2006). Uses and requirements of ecological niche models and related distributional models. *Biodivers. Inform.* 3 59–72. 10.17161/bi.v3i0.29

[B81] Peterson A. T. Papes M. Kluza D. A. (2003). Predicting the potential invasive distributions of four alien plant species in North America. *Weed Sci.* 51 863–868. 10.1614/P2002-081

[B82] Peterson A. T. Vieglais D. A. (2001). Predicting species invasions using ecological niche modeling: new approaches from bioinformatics attack a pressing problem. *Bioscience* 51 363–371. 10.1641/0006-3568(2001)051[0363:PSIUEN]2.0.CO;2

[B83] Phillips L. B. Hansen A. J. Flather C. H. (2008). Evaluating the species energy relationship with the newest measures of ecosystem energy: NDVI versus MODIS primary production. *Remote Sens. Environ.* 112 3538–3549. 10.1016/j.rse.2008.04.012

[B84] Phillips S. J. Anderson R. P. Schapire R. E. (2006). Maximum entropy modeling of species geographic distributions. *Ecol. Model.* 190 231–259. 10.1016/j.ecolmodel.2005.03.026

[B85] Phillips S. J. Dudík M. (2008). Modeling of species distributions with Maxent: new extensions and a comprehensive evaluation. *Ecography* 31 161–175. 10.1111/j.0906-7590.2008.5203.x

[B86] Pimentel D. Zuniga R. Morrison D. (2005). Update on the environmental and economic costs associated with alien-invasive species in the United States. *Ecol. Econ.* 52 273–288. 10.1016/j.ecolecon.2004.10.002

[B87] Porfirio L. L. Harris R. M. B. Lefroy E. C. Hugh S. Gould S. F. Lee G. (2014). Improving the use of species distribution models in conservation planning and management under climate change. *PLoS ONE* 9:e113749 10.1371/journal.pone.0113749 PMC4242662 25420020

[B88] Pottier J. Malenovský Z. Psomas A. Homolová L. Schaepman M. E. Choler P. (2014). Modelling plant species distribution in alpine grasslands using airborne imaging spectroscopy. *Biol. Lett.* 10 1–4. 10.1098/rsbl.2014.0347 PMC4126626 25079495

[B89] Pouteau R. Meyer J.-Y. Larrue S. (2015). Using range filling rather than prevalence of invasive plant species for management prioritisation: the case of *Spathodea campanulata* in the Society Islands (South Pacific). *Ecol. Indicat.* 54 87–95. 10.1016/j.ecolind.2015.02.017

[B90] Pradervand J. N. Dubuis A. Pellissier L. Guisan A. Randin C. (2014). Very high resolution environmental predictors in species distribution models: moving beyond topography? *Progr. Phys. Geogr.* 38 79–96. 10.1177/0309133313512667

[B91] Prates-Clark C. Saatchi S. S. Agosti D. (2008). Predicting geographical distribution models of high-value timber trees in the Amazon Basin using remotely sensed data. *Ecol. Model.* 211 309–323. 10.1016/j.ecolmodel.2007.09.024

[B92] Questad E. J. Kellner J. R. Kinney K. Cordell S. Asner G. P. Thaxton J. (2014). Mapping habitat suitability for at-risk plant species and its implications for restoration and reintroduction. *Ecol. Appl.* 24 385–395. 10.1890/13-0775.1 24689149

[B93] Radosavljevic A. Anderson R. P. (2014). Making better MaxEnt models of species distributions: complexity, overfitting and evaluation. *J. Biogeogr.* 41 629–643. 10.1111/jbi.12227

[B94] Radosevich S. R. Holt J. S. Ghersa C. M. (2007). *Ecology of Weeds Invasive Plants: Relationship to Agriculture and Natural Resource Management.* Hoboken, NJ: John Wiley and Sons 10.1002/9780470168943

[B95] Rameshprabu N. Swamy P. (2015). Prediction of environmental suitability for invasion of *Mikania micrantha* in India by species distribution modelling. *J. Environ. Biol.* 36 565–570.

[B96] Rew L. (2005). Predicting the occurrence of nonindigenous species using environmental and remotely sensed data. *Weed Sci.* 53 236–241. 10.1614/WS-04-097R

[B97] Running S. W. Nemani R. R. Heinsch F. A. Zhao M. Reeves M. Hashimoto H. (2004). A continuous satellite-derived measure of global terrestrial primary production. *BioScience* 54 547–560. 10.1641/0006-3568(2004)054[0547:ACSMOG]2.0.CO;2

[B98] Saatchi S. Buermann W. Ter Steege H. Mori S. Smith T. B. (2008). Modeling distribution of Amazonian tree species and diversity using remote sensing measurements. *Remote Sens. Environ.* 112 2000–2017. 10.1016/j.rse.2008.01.008

[B99] Schmidt M. Traore S. Ouedraogo A. Mbayngone E. Ouedraogo O. Zizka A. (2013). Geographical patterns of woody plants’ functional traits in Burkina Faso. *Candollea* 68 197–207. 10.15553/c2012v682a3

[B100] Simberloff D. (2000). Global climate change and introduced species in United States forests. *Sci. Total Environ.* 262 253–261. 10.1016/S0048-9697(00)00527-1 11087031

[B101] Sodhi N. S. Koh L. P. Brook B. W. Ng P. K. L. (2004). Southeast Asian biodiversity: an impending disaster. *Trends Ecol. Evolut.* 19 654–660. 10.1016/j.tree.2004.09.006 16701328

[B102] Sousa-Silva R. Alves P. Honrado J. Lomba A. (2014). Improving the assessment and reporting on rare and endangered species through species distribution models. *Glob. Ecol. Conserv.* 2 226–237. 10.1016/j.gecco.2014.09.011

[B103] Stohlgren T. J. Ma P. Kumar S. Rocca M. Morisette J. T. Jarnevich C. S. (2010). Ensemble habitat mapping of invasive plant species. *Risk Anal. Int. J.* 30 224–235. 10.1111/j.1539-6924.2009.01343.x 20136746

[B104] Strahler A. H. Boschetti L. Foody G. M. Friedl M. A. Hansen M. C. Herold M. (2006). *Global Land Cover Validation: Recommendations for Evaluation and Accuracy Assessment of Global Land Cover Maps*, Vol. 51 Luxembourg: European Communities.

[B105] Suding K. N. LeJeune K. D. Seastedt T. R. (2004). Competitive impacts and responses of an invasive weed: dependencies on nitrogen and phosphorus availability. *Oecologia* 141 526–535. 10.1007/s00442-004-1678-0 15375692

[B106] Swets J. A. (1988). Measuring the accuracy of diagnostic systems. *Science* 240 1285–1293. 10.1126/science.3287615 3287615

[B107] Synes N. W. Osborne P. E. (2011). Choice of predictor variables as a source of uncertainty in continental-scale species distribution modelling under climate change. *Divers. Distrib.* 20 904–914. 10.1111/j.1466-8238.2010.00635.x

[B108] Thi N. T. L. (2000). The Invasion of *Mimosa pigra* in Tram Chim National Park, Dong Thap Province. Ph.D. thesis, Ho Chi Minh City University of Science, Ho Chí Minh.

[B109] Thuiller W. (2005). Niche-based modelling as a tool for predicting the risk of alien plant invasions at a global scale. *Glob. Change Biol.* 11 2234–2250. 10.1111/j.1365-2486.2005.001018.x 34991288

[B110] Thuiller W. Araújo M. B. Lavorel S. (2004). Do we need land-cover data to model species distributions in Europe? *J. Biogeogr.* 31 353–361. 10.1046/j.0305-0270.2003.00991.x

[B111] Tuanmu M.-N. Jetz W. (2014). A global 1-km consensus land-cover product for biodiversity and ecosystem modelling. *Glob. Ecol. Biogeogr.* 23 1031–1045. 10.1111/geb.12182

[B112] Tuanmu M.-N. Viña A. Bearer S. Xu W. Ouyang Z. Zhang H. (2010). Mapping understory vegetation using phenological characteristics derived from remotely sensed data. *Remote Sens. Environ.* 114 1833–1844. 10.1016/j.rse.2010.03.008

[B113] Underwood E. Hollander A. Quinn J. (2013). *Geospatial Tools for Identifying and Managing Invasive Plants. Invasive Plant Ecology.* Boca Raton, FL: CRC Press.

[B114] USGS (1996). *GTOPO30 – Global Topographic Data.* Reston, VA: United States Geological Survey.

[B115] Valéry L. Fritz H. Lefeuvre J.-C. Simberloff D. (2008). In search of a real definition of the biological invasion phenomenon itself. *Biol. Invasions* 10 1345–1351. 10.1007/s10530-007-9209-7

[B116] Valéry L. Fritz H. Lefeuvre J.-C. Simberloff D. (2009). Ecosystem-level consequences of invasions by native species as a way to investigate relationships between evenness and ecosystem function. *Biol. Invasions* 11 609–617. 10.1007/s10530-008-9275-5

[B117] van Ewijk K. Y. Randin C. F. Treitz P. M. Scott N. A. (2014). Predicting fine-scale tree species abundance patterns using biotic variables derived from LiDAR and high spatial resolution imagery. *Remote Sens. Environ.* 150 120–131. 10.1016/j.rse.2014.04.026

[B118] Vanna S. Nang K. (2005). “Cambodia–The *Mimosa Pigra* Report,” in *Proceedings of the Asia-Pacific forest invasive species conference: The unwelcome guests*, Kuming.

[B119] Vitousek P. M. Walker L. R. (1989). Biological Invasion by *Myrica faya* in Hawai’i: plant demography, nitrogen fixation, ecosystem effects. *Ecol. Monogr.* 59 247–265. 10.2307/1942601

[B120] Waltari E. Schroeder R. McDonald K. Anderson R. P. Carnaval A. (2014). Bioclimatic variables derived from remote sensing: assessment and application for species distribution modelling. *Methods Ecol. Evolut.* 5 1033–1042. 10.1111/2041-210X.12264

[B121] Wang B. Li M. Liao W. Su J. Qiu H. Ding M. (2005). Geographical distribution of *Merremia boisiana*. *Ecol. Environ.* 14 451–454.

[B122] Wang C. Liu C. Wan J. Zhang Z. (2016). Climate change may threaten habitat suitability of threatened plant species within Chinese nature reserves. *PeerJ* 4:e2091 10.7717/peerj.2091 PMC4911960 27326373

[B123] Wilcove D. S. Rothstein D. Dubow J. Phillips A. Losos E. (1998). Quantifying threats to imperiled species in the United States. *BioScience* 48 607–615. 10.1007/s10661-016-5228-0

[B124] Wilson J. W. Sexton J. O. Jobe R. T. Haddad N. M. (2013). The relative contribution of terrain, land cover, and vegetation structure indices to species distribution models. *Biol. Conserv.* 164 170–176. 10.1016/j.biocon.2013.04.021

[B125] Wisz M. S. Hijmans R. J. Li J. Peterson A. T. Graham C. H. Guisan A. (2008). Effects of sample size on the performance of species distribution models. *Divers. Distrib.* 14 763–773. 10.1111/j.1472-4642.2008.00482.x

[B126] Wisz M. S. Pottier J. Kissling W. D. Pellissier L. Lenoir J. Damgaard C. F. (2013). The role of biotic interactions in shaping distributions and realised assemblages of species: implications for species distribution modelling. *Biol. Rev. Camb. Philos. Soc.* 88 15–30. 10.1111/j.1469-185X.2012.00235.x 22686347 PMC3561684

[B127] Wu L. Liang Y. Chen K. Li Z. Cao H. (2007). Damage and prevention of *Merremia boisiana* in Hainan Province, China. *Guangdong For. Sci.Technol.* 1 17.

[B128] Xu H. Qiang S. Genovesi P. Ding H. Wu J. Meng L. (2012). An inventory of invasive alien species in China. *NeoBiota* 15 1–26. 10.3897/neobiota.15.3575

[B129] Yan X. Zhenyu L. Gregg W. Dianmo L. (2001). Invasive species in China — an overview. *Biodivers. Conserv.* 10 1317–1341. 10.1023/A:1016695609745

[B130] Zellweger F. Braunisch V. Baltensweiler A. Bollmann K. (2013). Remotely sensed forest structural complexity predicts multi species occurrence at the landscape scale. *For. Ecol. Manag.* 307 303–312. 10.1016/j.foreco.2013.07.023

[B131] Zhu G. Li H. Zhao L. (2017). Incorporating anthropogenic variables into ecological niche modeling to predict areas of invasion of *Popillia japonica*. *J. Pest Sci.* 90 151–160., 10.1007/s10340-016-0780-5

[B132] Zhu L. Sun O. Sang W. Li Z. Ma K. (2007). Predicting the spatial distribution of an invasive plant species (*Eupatorium adenophorum*) in China. *Landsc. Ecol.* 22 1143–1154. 10.1007/s10980-007-9096-4

[B133] Zimmermann N. E. Edwards T. C. Moisen G. G. Frescino T. S. Blackard J. A. (2007). Remote sensing-based predictors improve distribution models of rare, early successional and broadleaf tree species in Utah. *J. Appl. Ecol.* 44 1057–1067. 10.1111/j.1365-2664.2007.01348.x 18642470 PMC2368764

