# Supplementary material for: Contemporary Remotely Sensed Data Products Refine Invasive Plants Risk Mapping in Data Poor Regions
Source: Front Plant Sci. 2017 May 15;8:770. doi: 10.3389/fpls.2017.00770 (PMC5430062; doi:10.3389/fpls.2017.00770)

SUPPORTING INFORMATION

Tuyet .T.A Truong, Giles E. St. J. Hardy, Margaret E. Andrew: Contemporary remotely sensed data products refine invasive plants risk mapping in data poor regions

S1 – Species specific variable importance & response curves

Variable importance of each variable among model sets was shown in Table 1 and mean variable importance of lifeforms and origin for COMB models in Table 2. Variable importance was estimated by permutation and quantified as the % reduction in model performance that occurred when a given variable was randomly permuted. CLIM includes only bioclimatic predictors; RS includes only remote-sensing predictors; COMB includes variables in CLIM and RS.

Marginal response curves are plotted for all variables with the importance above 5.0 for each species in COMB models. The orange curve in each plot is average response curve and the blue is standard deviation across all 10 partition runs.

Table 1: Variable importance of each variable among model sets

	Ac	*Ce*	*Ec*	*Mc*	*Ph*	*Co*	*Lc*	*Ll*	*Md*	*Mp*	*Bt*	*Mb*	*Mm*	*Pm*	Mean	SD
**CLIM**																
Annual Mean Temperature	10.02	20.61	9.18	28.25	12.43	2.66	8.18	1.65	27.27	52.09	11.74	0	0.83	0.86	13.27	14.57
Mean Diurnal Range	12.97	29.46	26.31	2.87	53.54	27.21	26.34	49.29	13.44	47.91	0	81.46	55.75	28.18	32.48	22.02
Isothermality	15.88	1.21	36.39	25.81	13.54	10.88	14.66	19.32	0.86	0	0.06	7.77	4.99	23.07	12.46	10.98
Annual precipitation	5.67	21.09	12.02	0.32	0.99	4.81	13.25	6.03	0	0	52.17	8.76	0.56	1.19	9.06	13.86
Precipitation of wettest month	13.83	2.99	0.54	1.56	3.86	5.14	5.05	0.63	1.45	0	0	0	1.9	8.66	3.26	2.52
Precipitation seasonality	10.15	0	13.26	20.97	10.04	0.53	10.5	3.09	0	0	0.22	0.92	0.06	9.43	5.66	6.56
Precipitation of warmest quarter	31.48	24.64	2.28	20.22	5.61	48.76	22.03	20	56.98	0	35.81	1.08	35.91	28.61	23.81	17.41
**RS**																
GPP_CV	7.21	0.92	0.51	24.85	6.95	17.92	7.66	15.77	21.27	0	0	0	18.07	29.53	10.76	10.24
GPP_Mean	7.38	7.16	0.67	24.76	10.02	3.07	13.1	21.04	18.15	0	0	0	3.31	9.02	8.41	8.2
Soil pH	1.37	1.87	0.08	0.31	20.72	1.99	0.19	0.88	2.25	0	0	0	3.57	1.92	2.51	5.34
Barren	1.42	0	1.58	4.48	2.56	2.1	1.88	1.57	0	5.61	7.01	5.27	1.9	1.49	2.63	2.09
Cultivated vegetation	15.82	11.01	24.76	22.65	5.68	15.46	13.9	10.1	1.22	0	12.77	0	13.97	9.72	11.22	7.51
Deciduous broadleaf trees	9.65	3.68	2.34	2.3	7.7	8.02	4.92	6.54	20.07	31.62	0.33	13.4	7.84	5.56	8.86	8.25
Evergreen broadleaf trees	7.61	28.33	34.14	1.56	14.07	11.27	17	14.82	5.46	0	18.55	0	8.64	11.72	12.37	9.93
Evergreen needleleaf trees	5.53	2.86	0.23	0.97	0.1	3.89	3.86	4.03	2.79	31.56	1.1	24.01	3.73	1.95	6.19	9.4
Herbaceous vegetation	15.08	20.56	13.94	9.93	22.17	11.47	16.24	4.9	20.93	24.2	16.42	38.87	13.78	5.48	16.71	8.62
Mixed trees	12.6	17.68	17.6	1	7.65	8.52	8.61	9.55	1.35	5.35	0	0	14.22	14.35	8.46	6.18
Open water	3.25	0.8	0.37	0.33	0.06	1.63	1.9	2.54	0.35	1.66	0.94	0.04	1.25	1.66	1.2	0.77
Regular flooded vegetation	1.89	0.14	0.08	0.74	0.03	1.82	1.42	1.56	0.08	0	6.54	18.4	1.93	0.79	2.53	4.86
Shrubs	6.72	4.89	2.66	0.77	0.36	11.22	7.91	5.11	5.8	0	36.34	0	6.62	3.44	6.56	9.19
Urban	4.49	0.09	1.04	5.34	1.94	1.62	1.41	1.61	0.27	0	0	0	1.18	3.36	1.6	1.49
**COMB**																
GPP_CV	0.6	0.17	0.64	5.51	2.23	2.78	2.15	2.73	9.1	0	0	0	1.85	1.65	2.1	2.49
GPP_Mean	4.23	8.68	1.34	2.24	1.71	0.52	5.07	9.81	4.66	0	0	0	0.11	1.29	2.83	3.21
Soil pH	3.96	2.26	0.02	1.79	1.18	2.76	0.7	1.99	0.29	0	0	0	1.77	1.69	1.32	0.95
Barren	0.46	0.02	2.66	1.05	0.03	1.1	1.03	0.97	0.08	2.95	2.56	3.6	0.32	0.12	1.21	1.2
Cultivated vegetation	4.58	2.84	21.8	1.45	0.01	1.08	6.78	3.01	0.07	0	6.48	0	2.85	2.71	3.83	5.64
Deciduous broadleaf trees	3.25	5.49	1.63	0.53	2.56	5.23	1.63	5.24	15.22	14.69	0.47	9.25	6.1	1.13	5.17	4.81
Evergreen broadleaf trees	6.55	18.25	33.2	0.82	0.99	3.07	11.18	8.9	6.33	0	0	0	4.31	5.8	7.1	9.11
Evergreen needleleaf trees	1.59	4.09	0.32	1.36	0.18	0.73	2.96	3.55	3.54	36.32	2.04	3.21	1.16	0.87	4.42	9.24
Herbaceous vegetation	2.65	4.7	9.84	5.41	0.14	1.56	5.16	2.57	10.4	26.26	7.91	19.18	0.88	2.02	7.05	7.38
Mixed trees	5.23	8.45	18.42	0.42	1.15	1.52	5.05	3.07	0	0	0	0	5.81	2.61	3.7	4.99
Open water	1.33	0.77	0.3	0.03	0	0.36	0.97	0.66	0.03	2.6	0.95	0	2.24	0.81	0.79	0.8
Regular flooded vegetation	0.58	0.11	0.11	0.05	0.01	1.05	0.42	0.37	0.75	0	4.26	5.08	0.64	0.29	0.98	1.6
Shrubs	2.01	3.59	3.58	0.65	0.02	1.75	3.43	3.15	3.38	0	0	0	1.49	1.75	1.77	1.46
Urban	2.03	0.11	0.98	1.1	0.45	1.51	0.93	3.02	0.11	0	0	0.56	0.11	4.04	1.07	1.19
Annual Mean Temperature	7.41	0.09	0.18	25.07	2.8	0.04	1.19	1.43	8.03	6.38	4.9	0.09	0	2.84	4.32	6.57
Mean Diurnal Range	8.3	9.92	1.2	1.87	52.26	23.02	9.1	17.15	14.15	9.53	0	41.37	40.36	18.82	17.65	16.04
Isothermality	9.69	1.86	1.1	20.06	17.65	11.19	5.17	14.14	1.39	1.27	0	6.51	2.91	15.08	7.72	6.84
Annual precipitation	4.54	7.03	0.33	0.14	2.46	4.01	14.6	3.38	0	0	54.28	11.16	1.39	2.12	7.53	14.12
Precipitation of wettest month	6.71	0.72	0.05	1.05	1.66	0.78	0.35	0.87	0.82	0	0	0	0.94	7.66	1.54	1.94
Precipitation seasonality	5.12	2.21	2.21	19.16	3.25	1.15	7.03	3.34	0.05	0	0.65	0	4.07	3.19	3.67	4.9
Precipitation of warmest quarter	19.17	18.64	0.11	10.22	9.24	34.79	15.11	10.63	21.6	0	15.49	0	20.68	23.52	14.23	9.93

Ac: Ageratum conyzoides, Ce: Cenchrus echinatus Ec: Eichhornia crassipes, Mc: Microstegium ciliatum, Ph: Parthenium hysterophorus, Co: Chromolaena odorata, Lc: Lantana camara, Ll: Leucaena leucocephala, Md: Mimosa diplotricha, Mp: Mimosa pigra, Bt: Bauhinia touranensis, Mb: Merremia boisiana, Mm: Mikania micrantha, Pm:Pueraria montana

Table 2: Summary of the permutation importance (PI) for each plant type and originin COMB models. The values (plus or minus standard errors) represent average PI. Mean values were calculated from the average of fourteen species. Values in bold indicate variables with above-average importance.

Variables	Herb	Shrub	Vine	Native	Non-native	Mean
GPP_CV	1.83 ± 2.2	3.35 ± 3.41	0.87 ± 1.01	1.79 ± 2.60	2.23 ± 2.63	2.10 ± 2.49
GPP_Mean	3.64 ± 3.03	4.01 ± 3.98	0.35 ± 0.63	0.88 ± 1.09	3.61 ± 3.52	2.83 ± 3.21
Soil pH	1.84 ± 1.45	1.15 ± 1.18	0.87 ± 1.00	0.87 ± 1.00	1.49 ± 1.30	1.32 ± 0.95
Barren	0.84 ± 1.1	1.22 ± 1.05	1.65 ± 1.71	1.83 ± 1.55	0.96 ± 1.06	1.21 ± 1.20
Cultivated vegetation	**6.14 ± 8.92**	2.19 ± 2.84	3.01 ± 2.66	2.66 ± 2.78	4.30 ± 6.53	3.83 ± 5.64
Deciduous broad leaf trees	2.69 ± 1.87	**8.4 ± 6.16**	4.23 ± 4.18	2.84 ± 4.28	**6.10 ± 4.94**	**5.17 ± 4.81**
Evergreen broad leaf trees	**11.96 ± 13.82**	**5.9 ± 4.47**	2.53 ± 2.98	1.65 ± 2.79	**9.28 ± 9.95**	**7.10 ± 9.11**
Evergreen needle leaf trees	1.51 ± 1.57	**9.42 ± 15.08**	1.82 ± 1.05	1.87 ± 1.01	**5.44 ± 10.94**	4.42 ± 9.24
**Herbaceous vegetation**	4.55 ± 3.6	**9.19 ± 10.14**	**7.5 ± 8.38**	**8.63 ± 7.44**	**6.42 ± 7.81**	**7.05 ± 7.38**
Mixed trees	**6.73 ± 7.29**	1.93 ± 2.16	2.11 ± 2.76	0.76 ± 1.25	**4.87 ± 5.51**	3.70 ± 4.99
Open water	0.49 ± 0.56	0.92 ± 1.00	1 ± 0.93	0.45 ± 0.51	0.93 ± 0.89	0.79 ± 0.80
Regular flooded vegetation	0.17 ± 0.23	0.52 ± 0.40	2.57 ± 2.46	2.42 ± 2.62	0.40 ± 0.35	0.98 ± 1.60
Shrubs	1.97 ± 1.64	2.34 ± 1.48	0.81 ± 0.94	0.60 ± 0.82	2.24 ± 1.41	1.77 ± 1.46
Urban	0.93 ± 0.73	1.12 ± 1.23	1.18 ± 1.92	1.42 ± 1.80	0.93 ± 1.00	1.07 ± 1.19
Annual mean temperature	**7.11 ± 10.47**	3.41 ± 3.55	1.96 ± 2.37	**8.23 ± 11.40**	2.76 ± 3.26	4.32 ± 6.57
**Mean diurnal temperature**	**14.71 ± 21.34**	**14.59 ± 5.78**	**25.14 ± 19.72**	**15.51 ± 19.20**	**18.50 ± 16.00**	**17.65 ± 16.04**
**Isothermality**	**10.07 ± 8.74**	**6.63 ± 5.82**	**6.12 ± 6.54**	**10.41 ± 8.92**	**6.64 ± 6.09**	**7.72 ± 6.84**
**Annual precipitation**	2.9 ± 2.92	4.4 ± 6	**17.24 ± 25.09**	**16.93 ± 25.36**	3.77 ± 4.43	**7.53 ± 14.12**
Precipitation of wettest month	2.04 ± 2.67	0.56 ± 0.38	2.15 ± 3.7	2.18 ± 3.69	1.29 ± 1.96	1.54 ± 1.94
Precipitation of seasonality	**6.39 ± 7.24**	2.31 ± 2.97	1.98 ± 1.96	5.75 ± 9.04	2.84 ± 2.22	3.67 ± 4.90
**Precipitation of warmest quarter**	**11.48 ± 7.85**	**16.42 ± 12.93**	**14.92 ± 10.49**	**12.31 ± 9.86**	**15.00 ± 10.53**	**14.23 ± 9.93**

Ageratum conyzoides

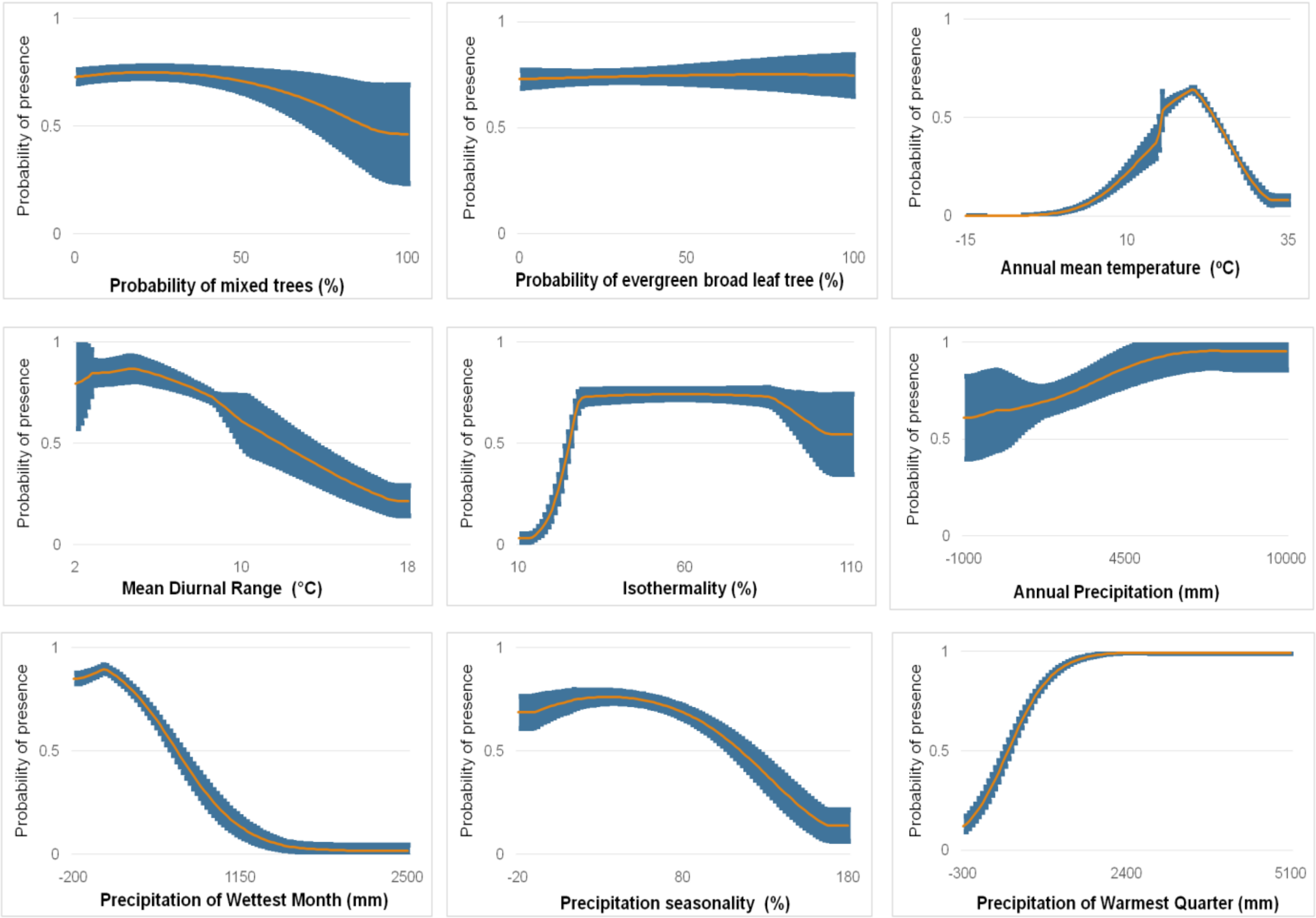

Cenchrus echinatus

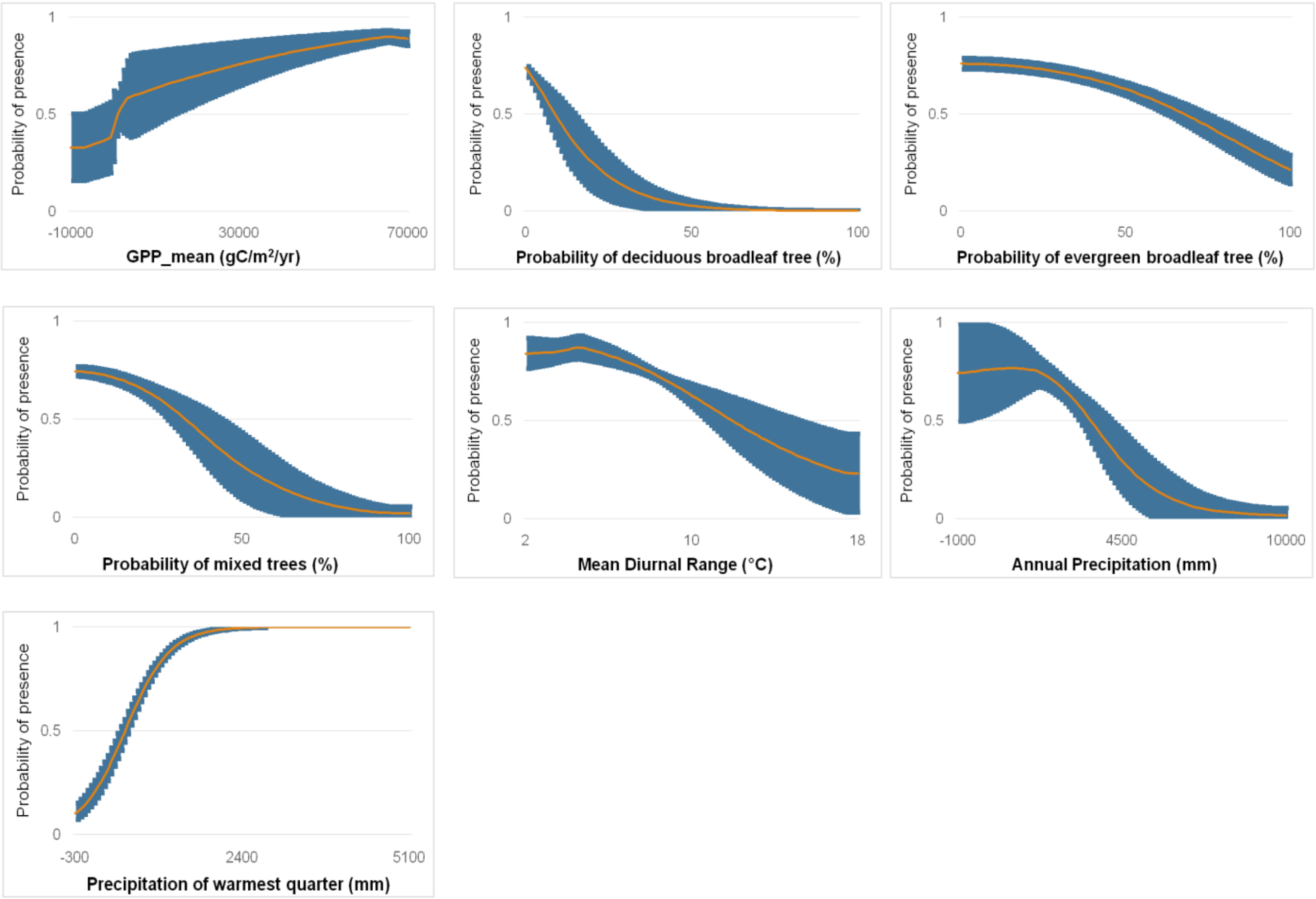

Eichhornia crassipes

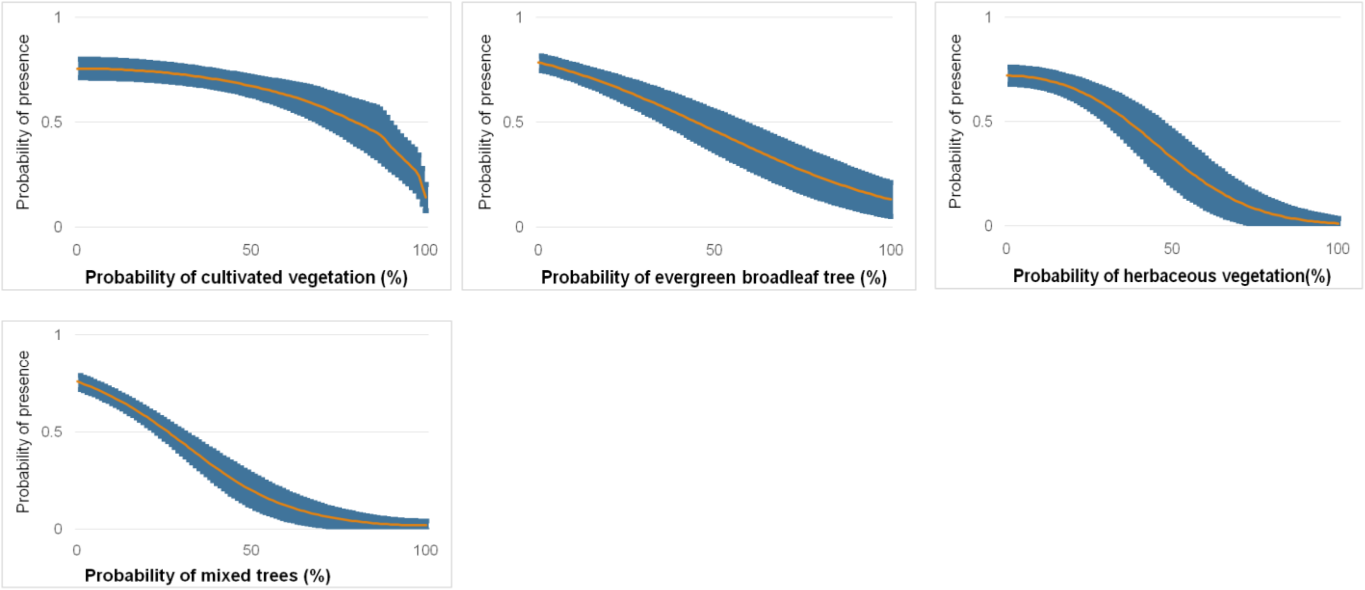

Microstegium ciliatum

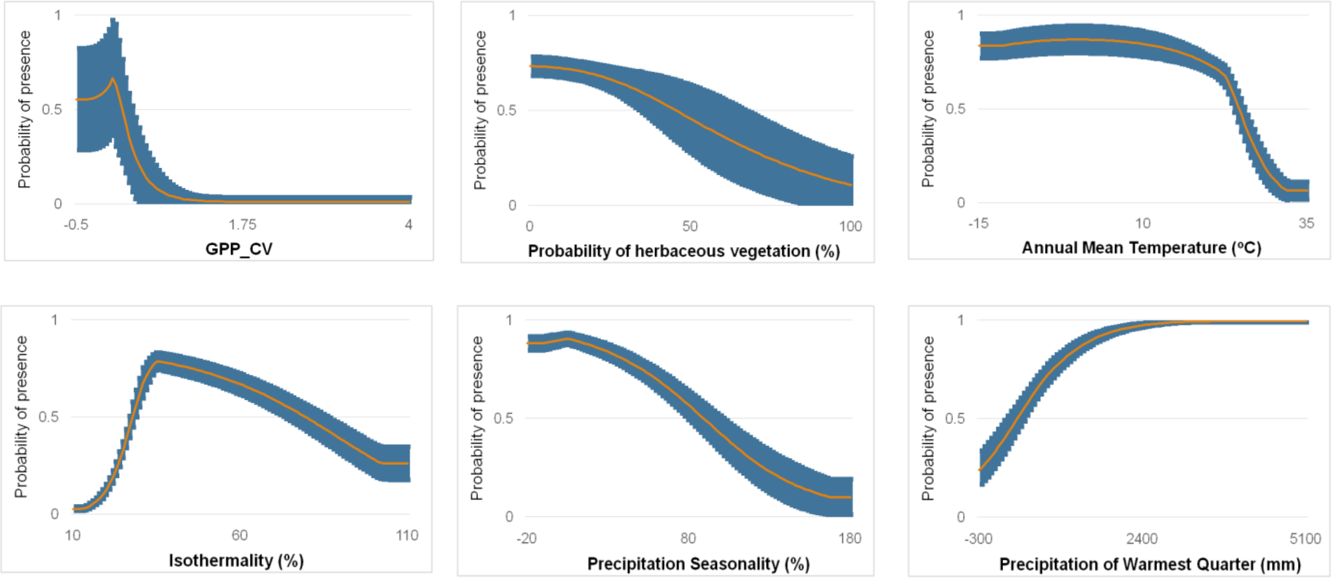

Parthenium hysterophorus

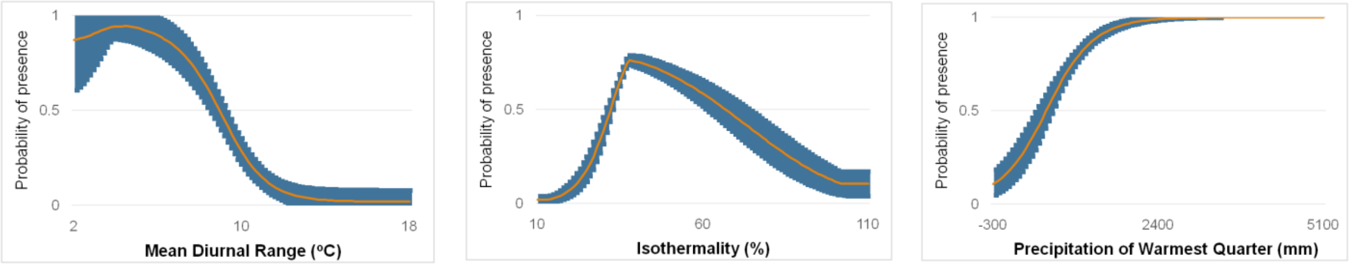

Chromolaena odorata

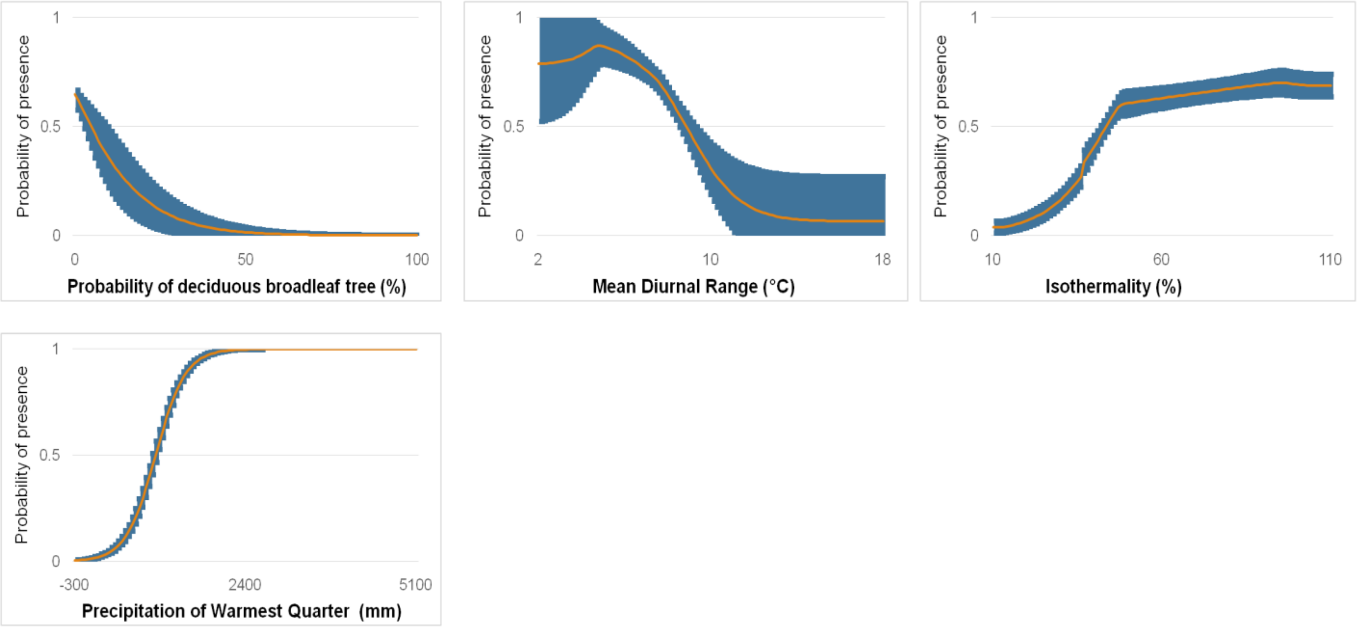

Lantana camara

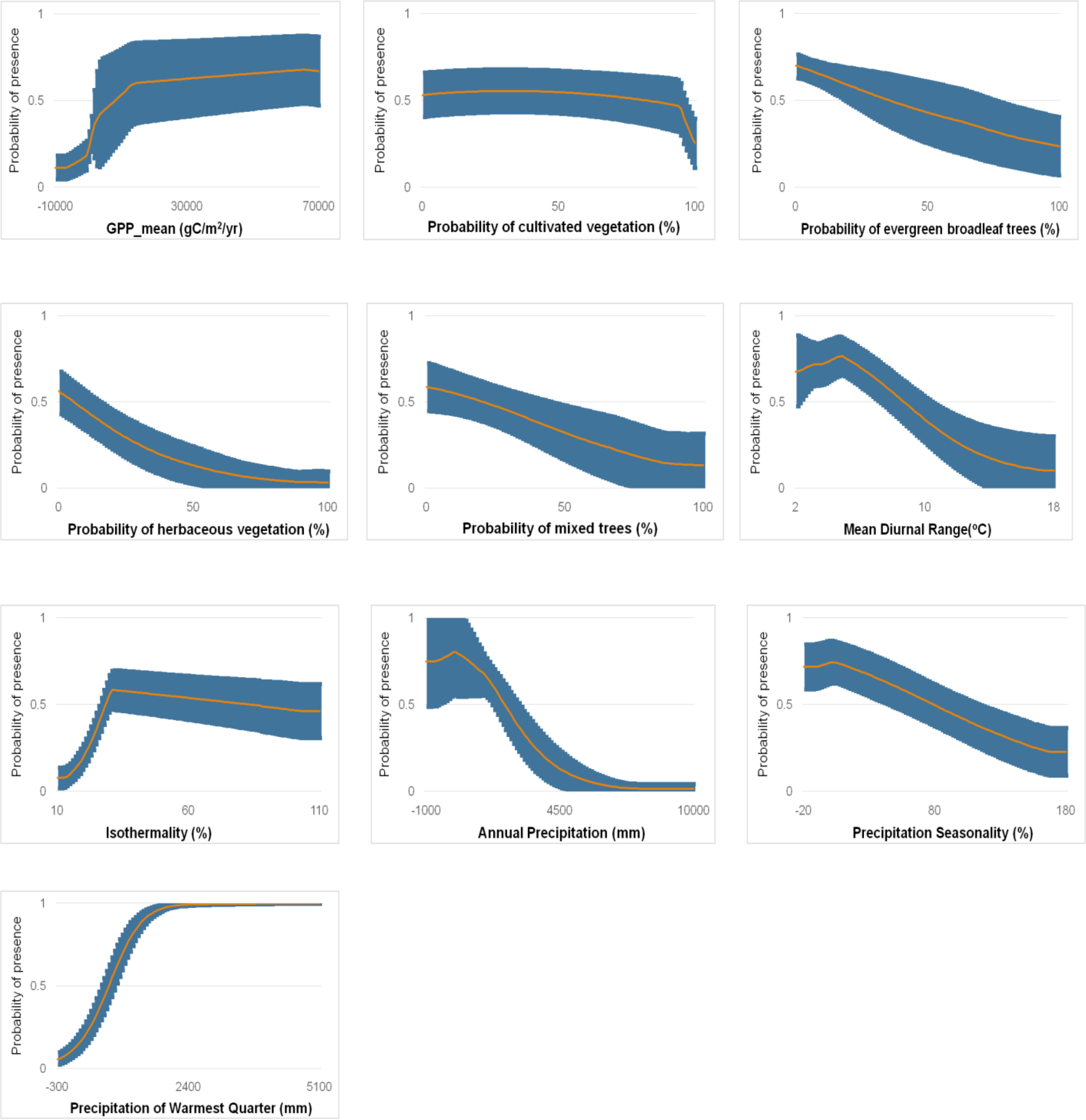

Leucaena leucocephala

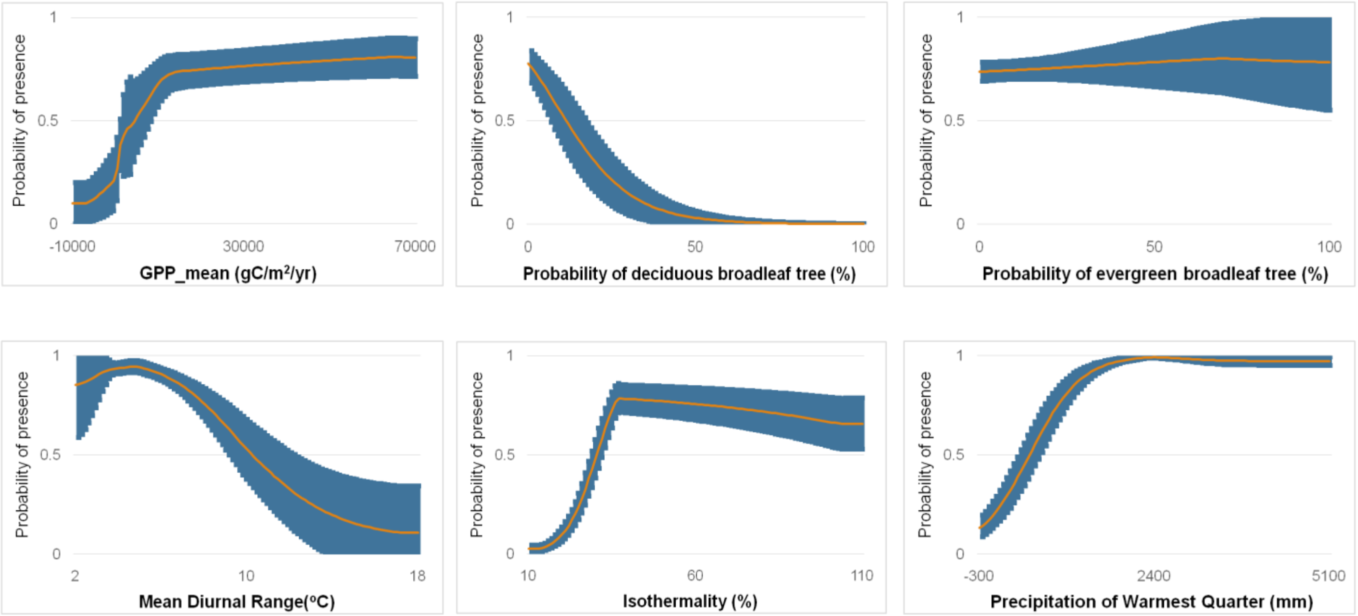

Mimosa diplotricha

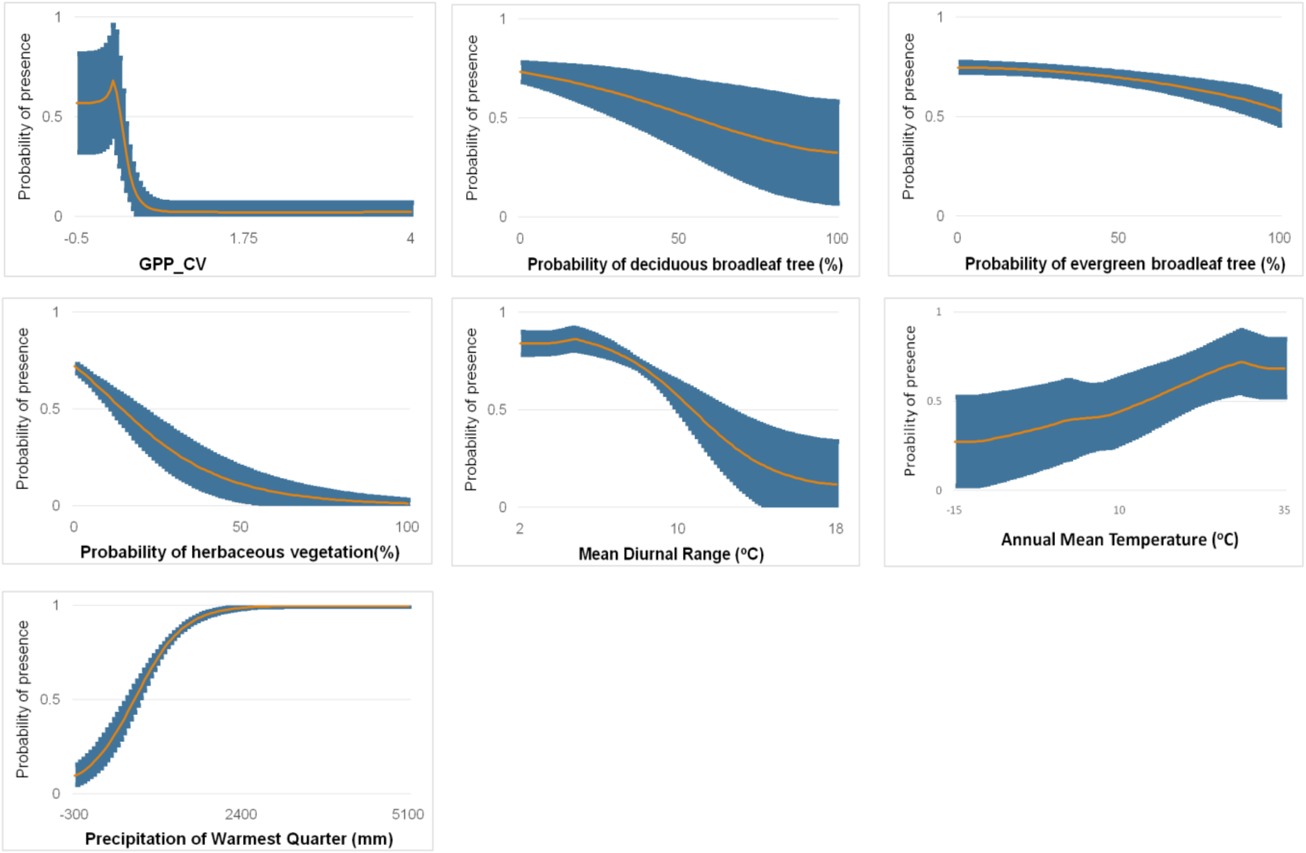

Mimosa pigra

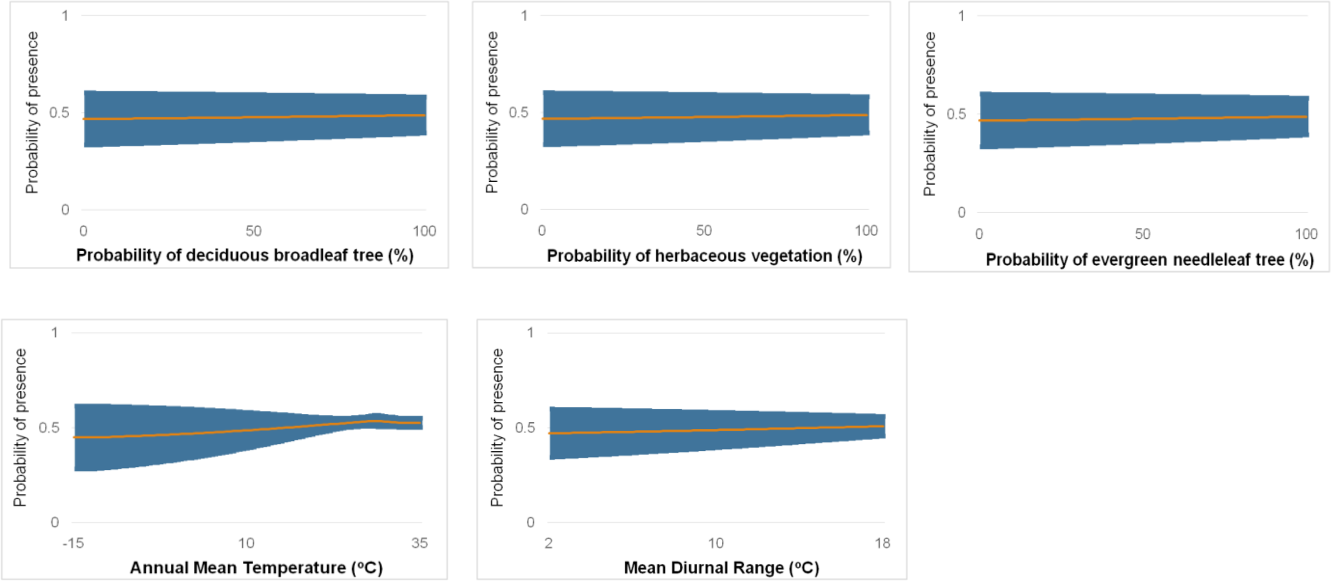

Bauhinia touranensis

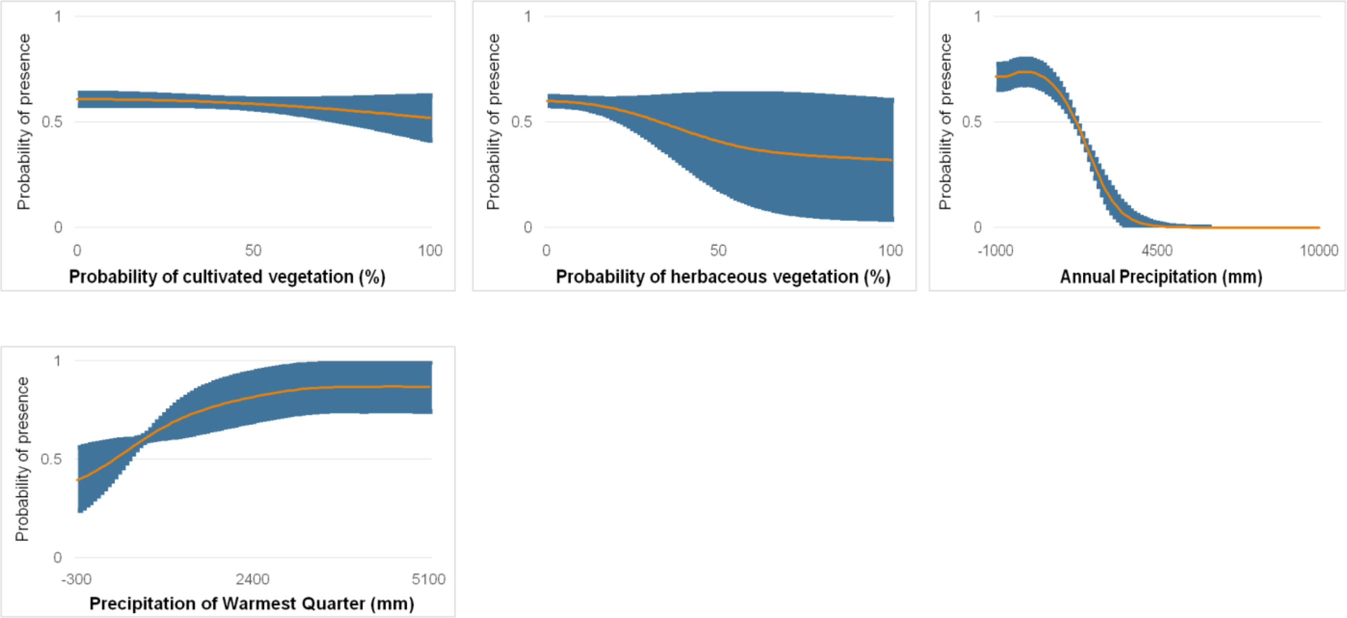

Merremia boisiana

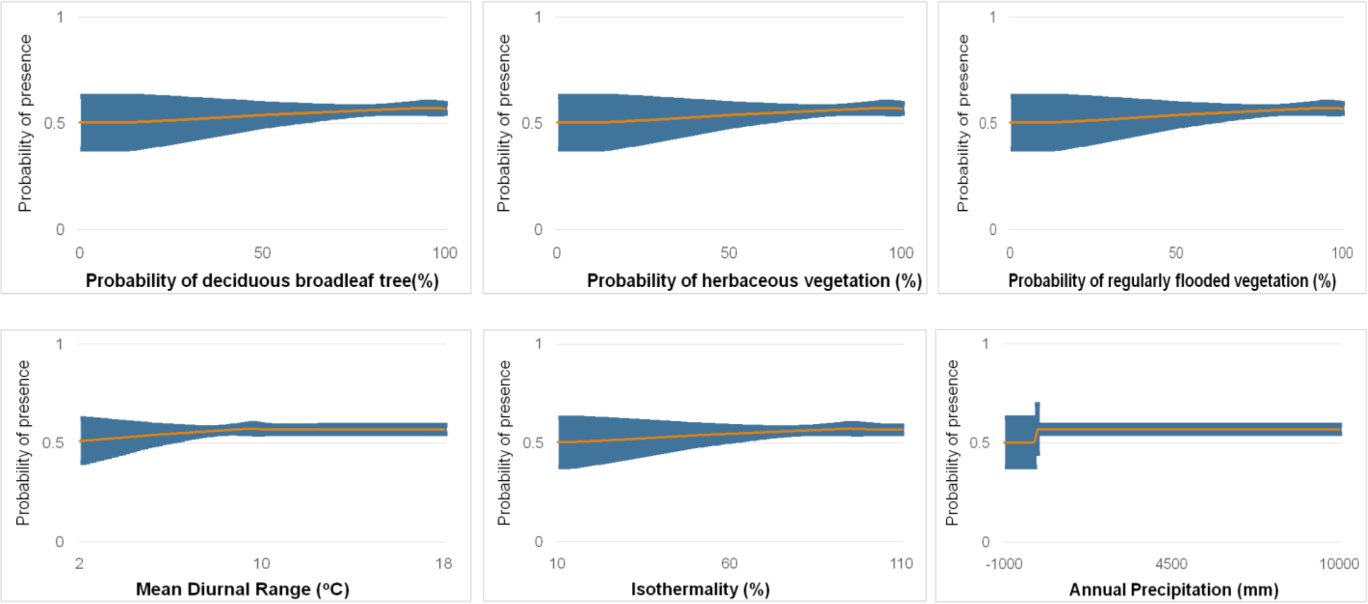

Mikania micrantha

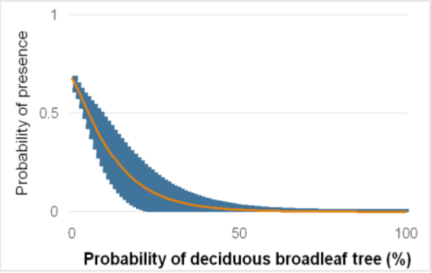

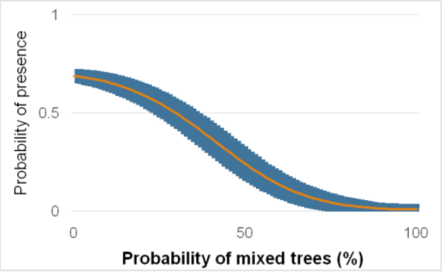

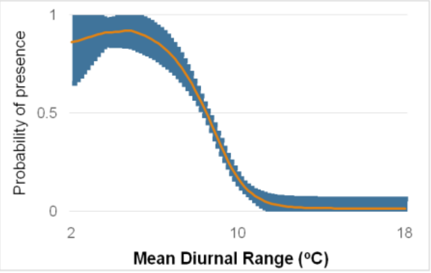

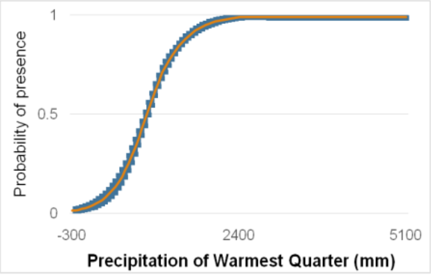

Pueraria montana

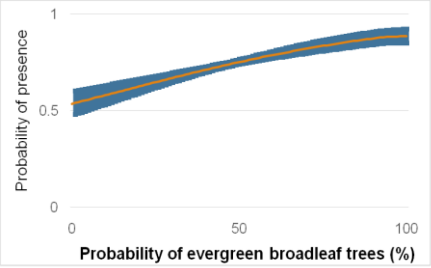

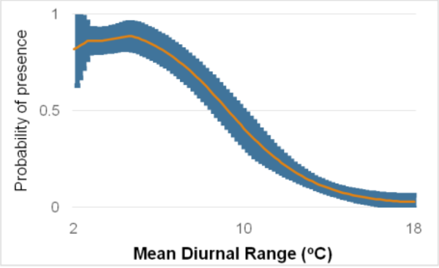

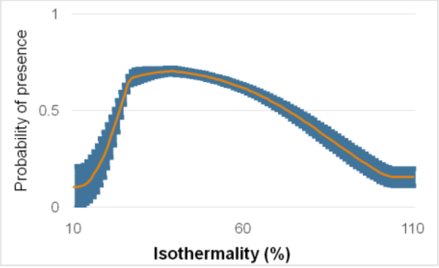

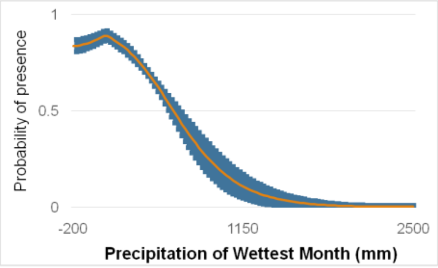

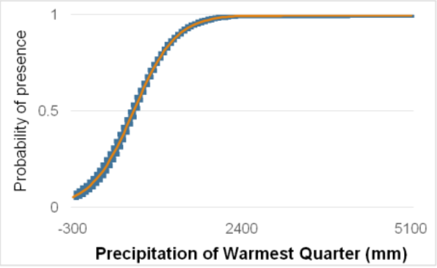

Supplement: Supplementary file 1 [file Data_Sheet_1.pdf]
