# Supplementary material for: Contemporary Remotely Sensed Data Products Refine Invasive Plants Risk Mapping in Data Poor Regions
Source: Front Plant Sci. 2017 May 15;8:770. doi: 10.3389/fpls.2017.00770 (PMC5430062; doi:10.3389/fpls.2017.00770)
Supplement: Supplementary file 2 [file Data_Sheet_2.pdf]

SUPPORTING INFORMATION

Tuyet .T.A Truong, Giles E. St. J. Hardy, Margaret E. Andrew: Contemporary remotely sensed data products refine invasive plants risk mapping in data poor regions

S2: Habitat suitability maps based on climate and remote sensing data (COMB), Climate (CLIM) and Remote sensing data (RS). The predicted area and percentage of agreements of each species among model sets were presented in bar graphs below maps

Ageratum conyzoides

Habitat suitability based on climate and remote sensing data

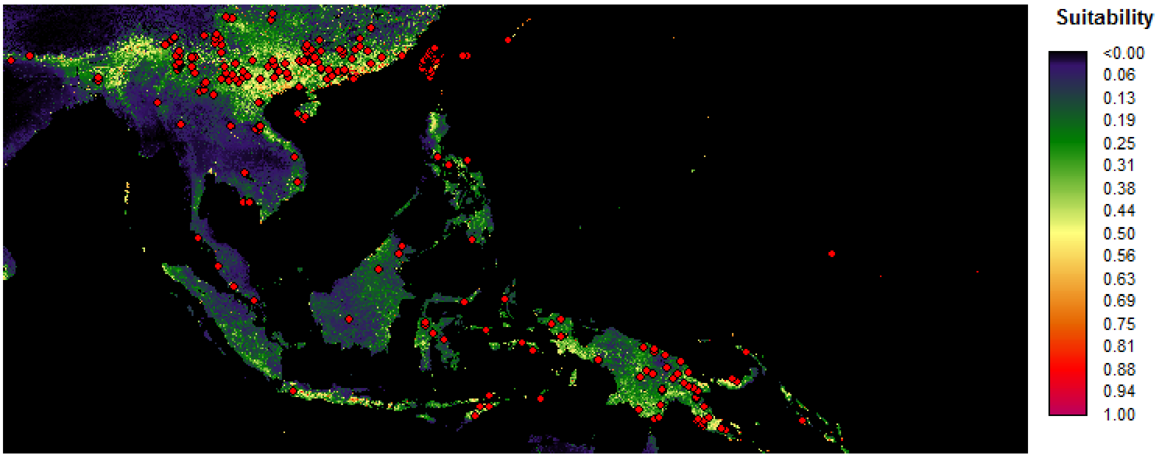

Ageratum conyzoides

Habitat suitability based on climate data

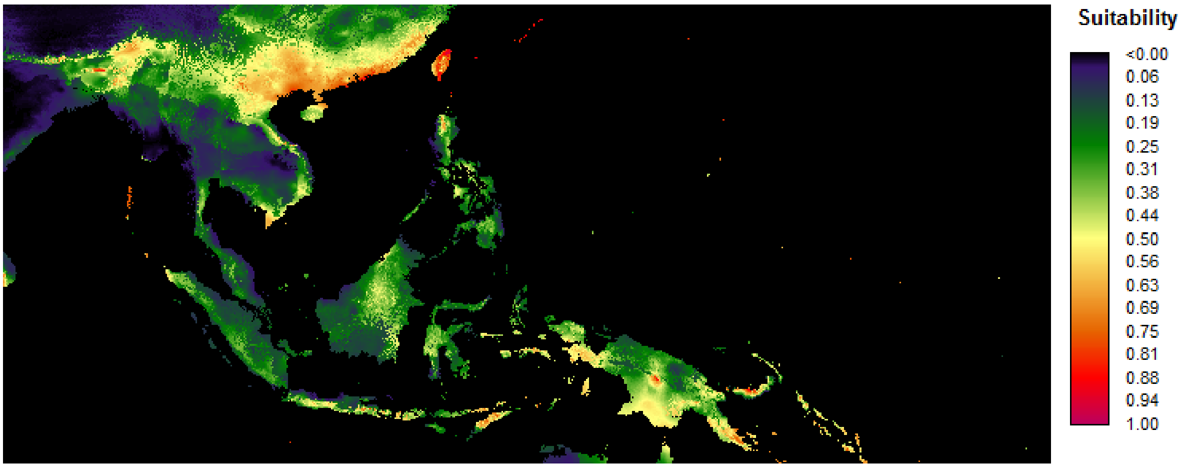

Ageratum conyzoides

Habitat suitability based on remote sensing data

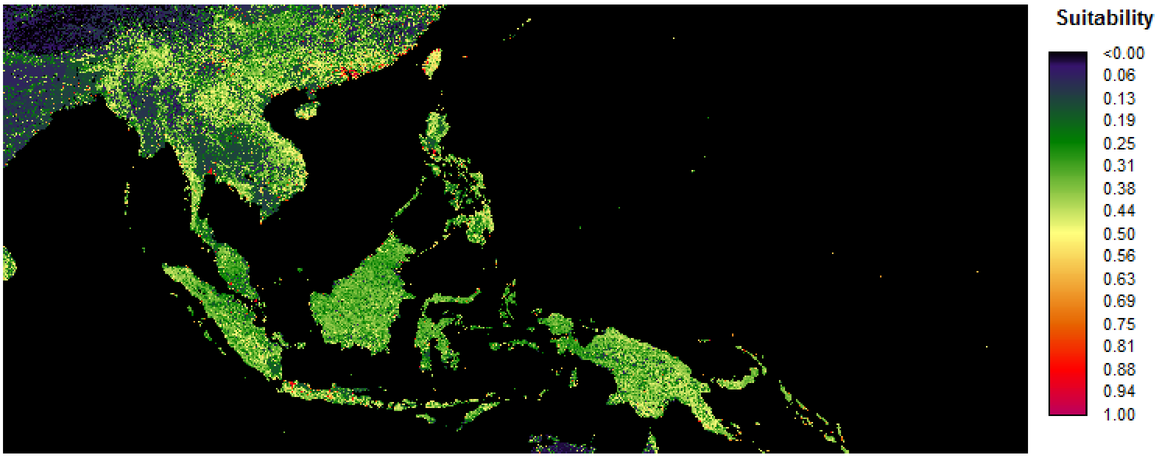

Bauhinia touranensis

Habitat suitability based on climate and remote sensing data

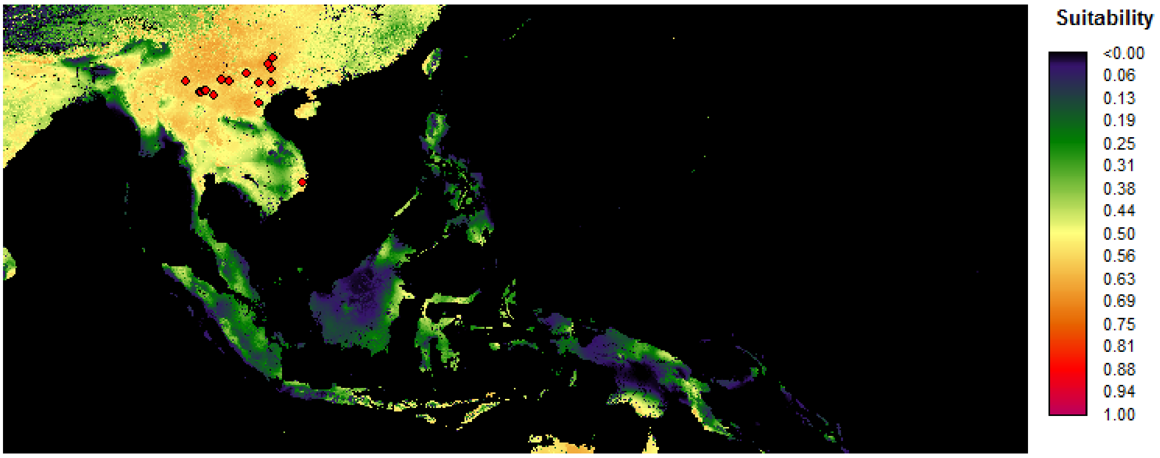

Bauhinia touranensis

Habitat suitability based on climate data

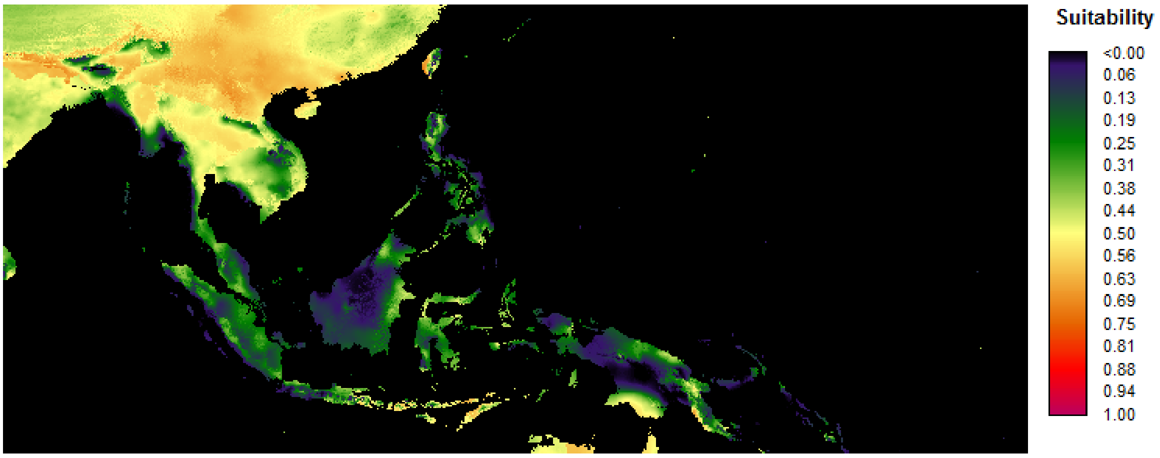

Bauhinia touranensis

Habitat suitability based on remote sensing data

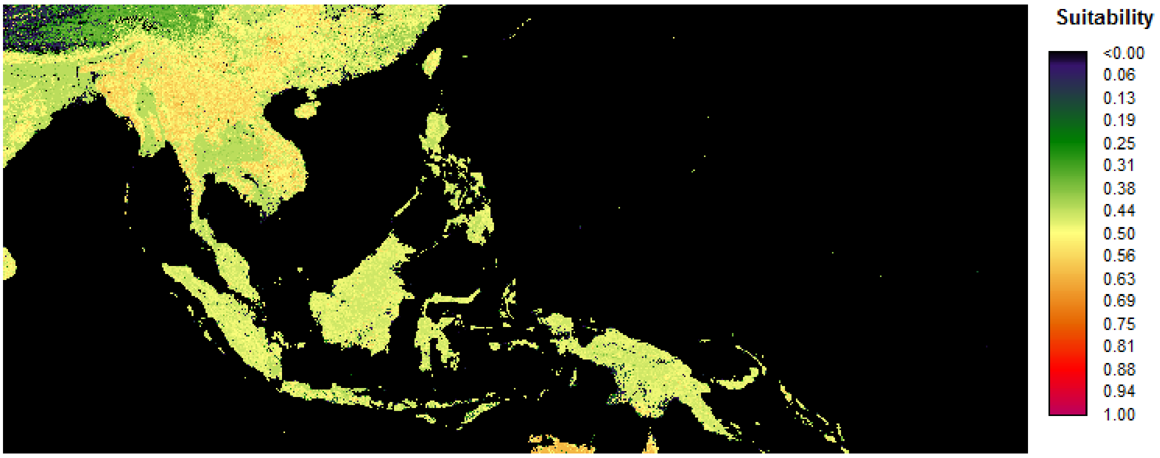

Cenchrus echinatus

Habitat suitability based on climate and remote sensing data

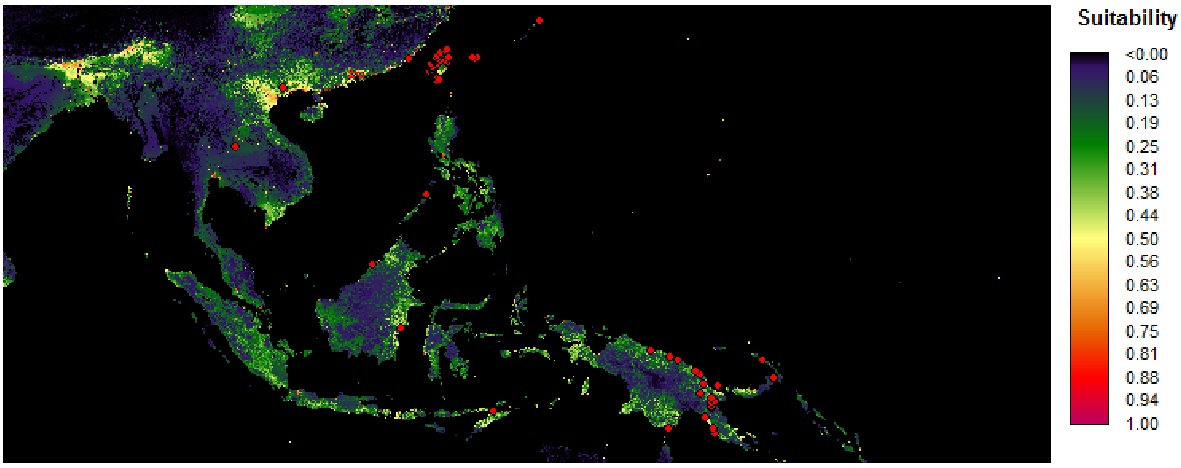

Cenchrus echinatus

Habitat suitability based on climate data

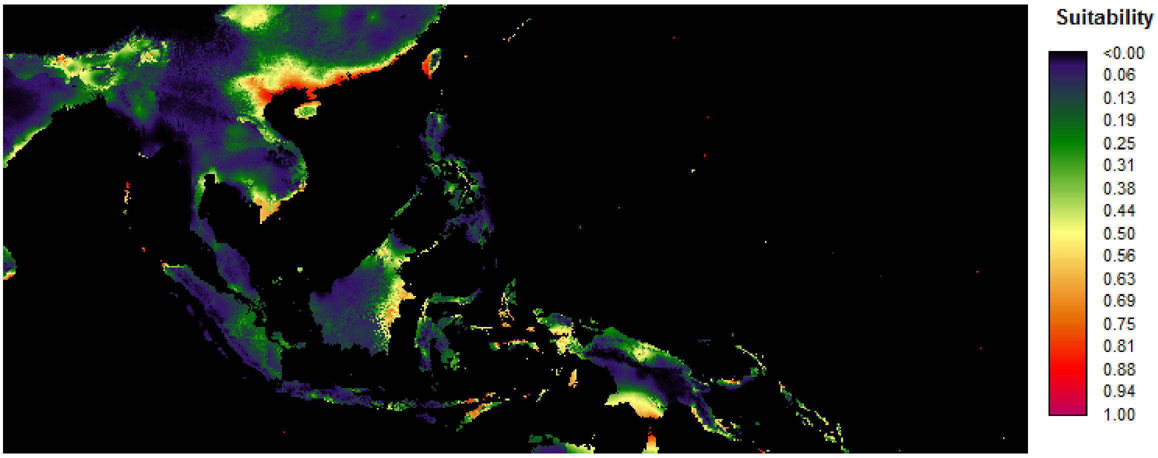

Cenchrus echinatus

Habitat suitability based on remote sensing data

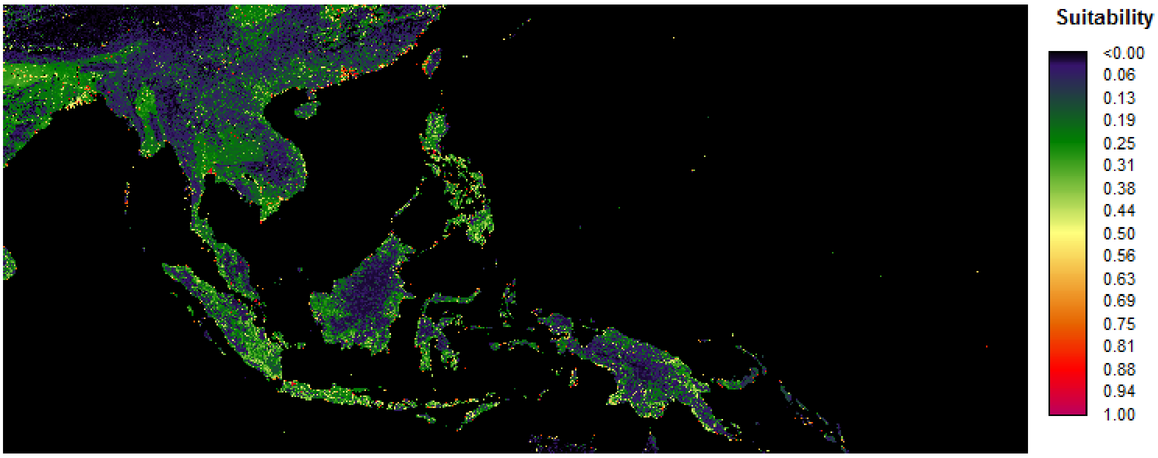

Chromolaena odorata

Habitat suitability based on climate and remote sensing data

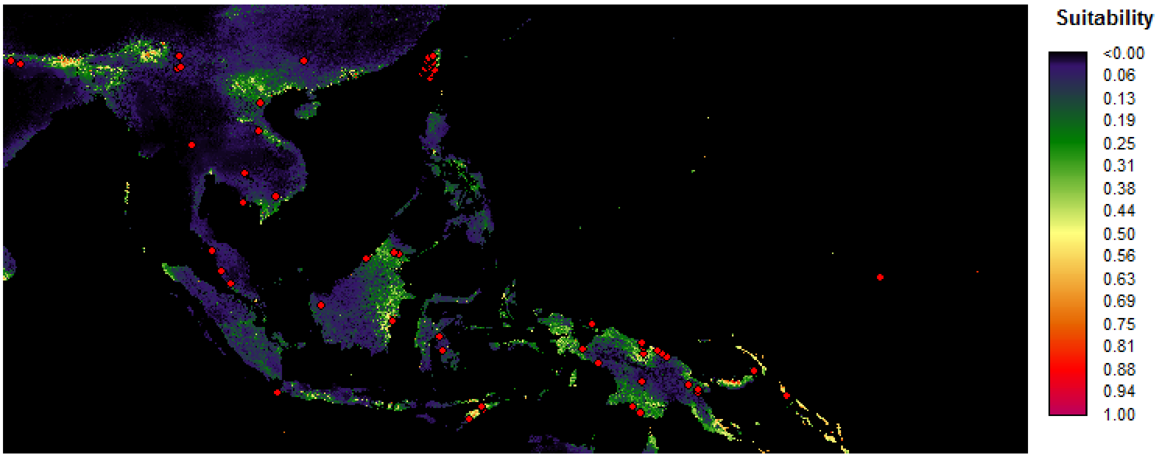

Chromolaena odorata

Habitat suitability based on climate data

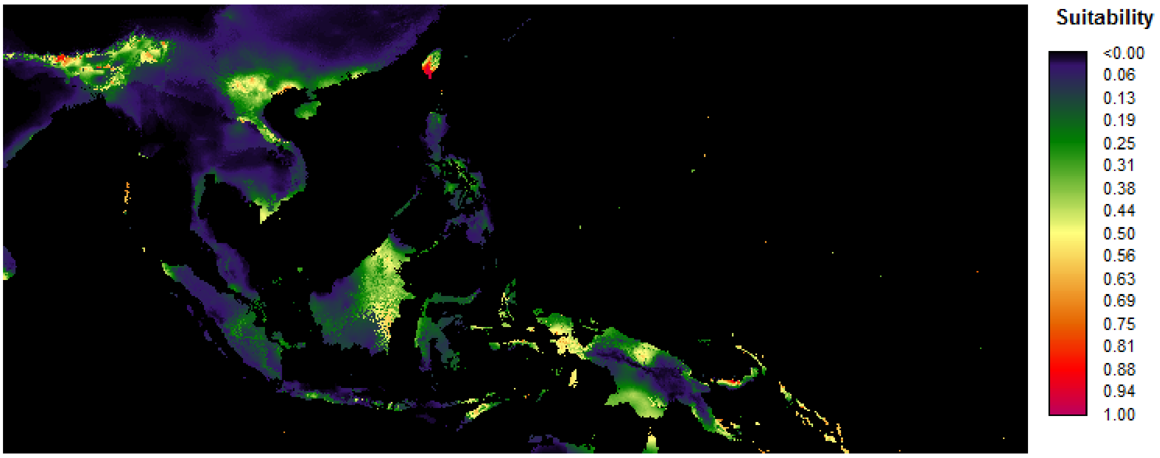

Chromolaena odorata

Habitat suitability based on remote sensing data

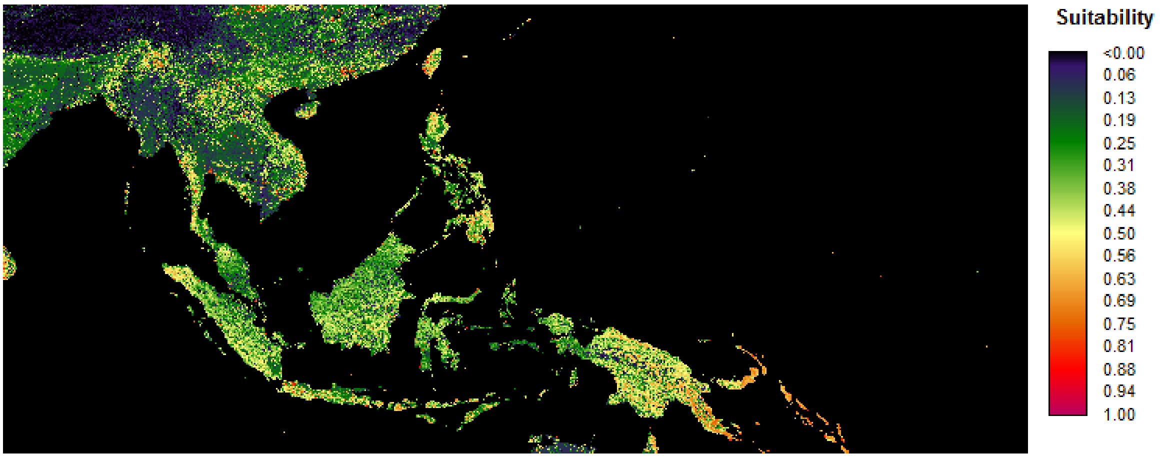

Eichhornia crassipes

Habitat suitability based on climate and remote sensing data

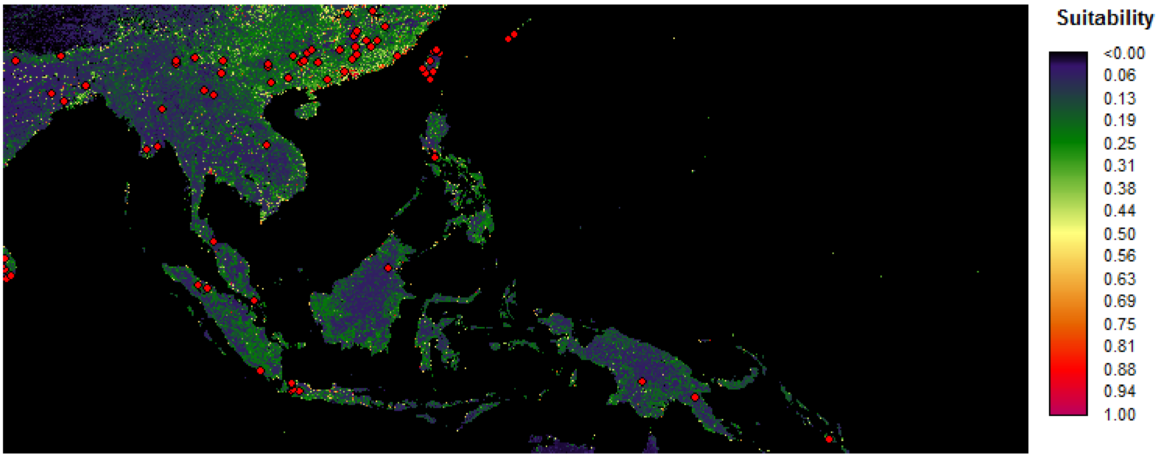

Eichhornia crassipes

Habitat suitability based on climate data

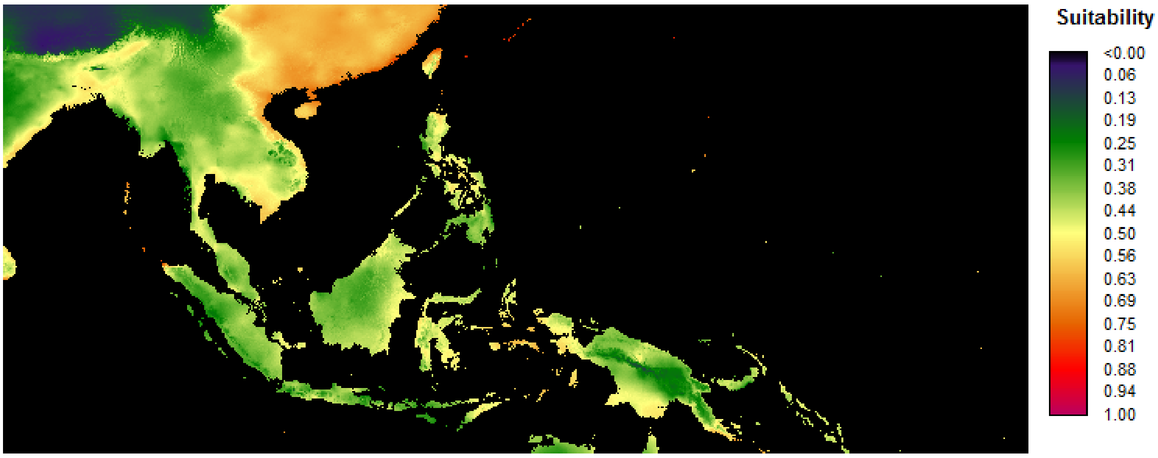

Eichhornia crassipes

Habitat suitability based on remote sensing data

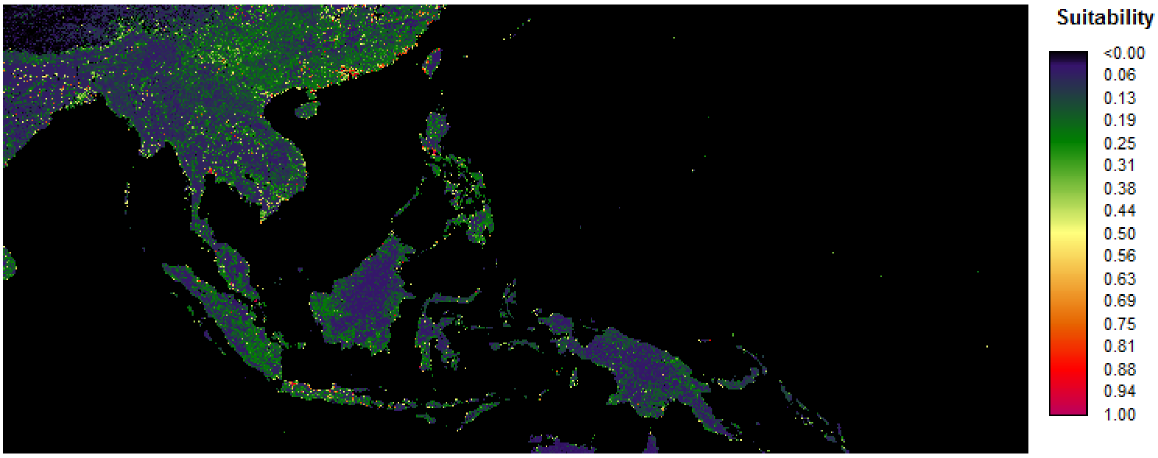

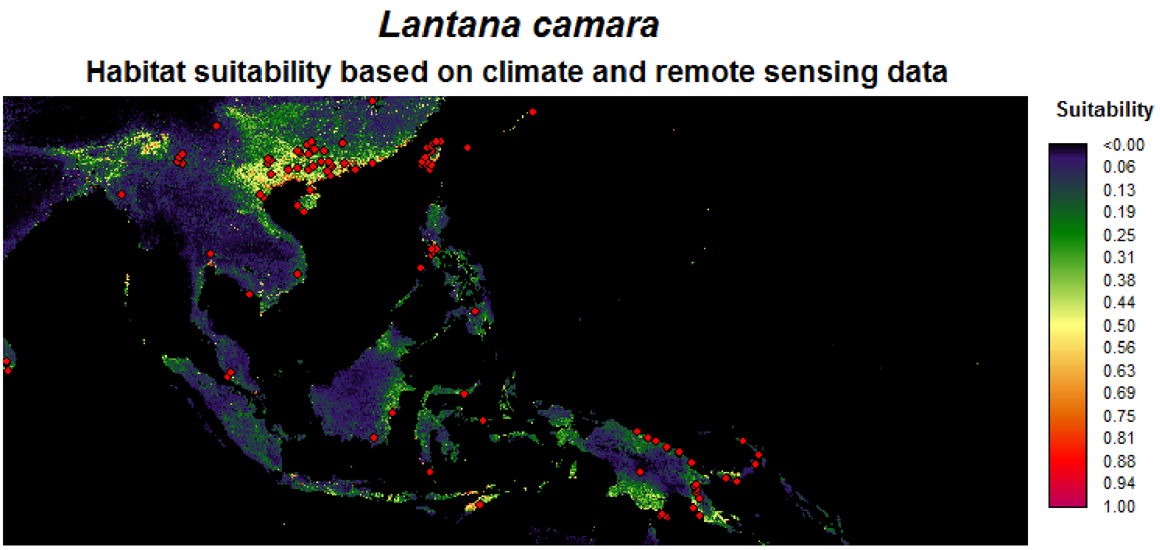
Lantana camara
Habitat suitability based on climate and remote sensing data
Suitability
<0.00
0.06
0.13
0.19
0.25
0.31
0.38
0.44
0.50
0.56
0.63
0.69
0.75
0.81
0.88
0.94
1.00

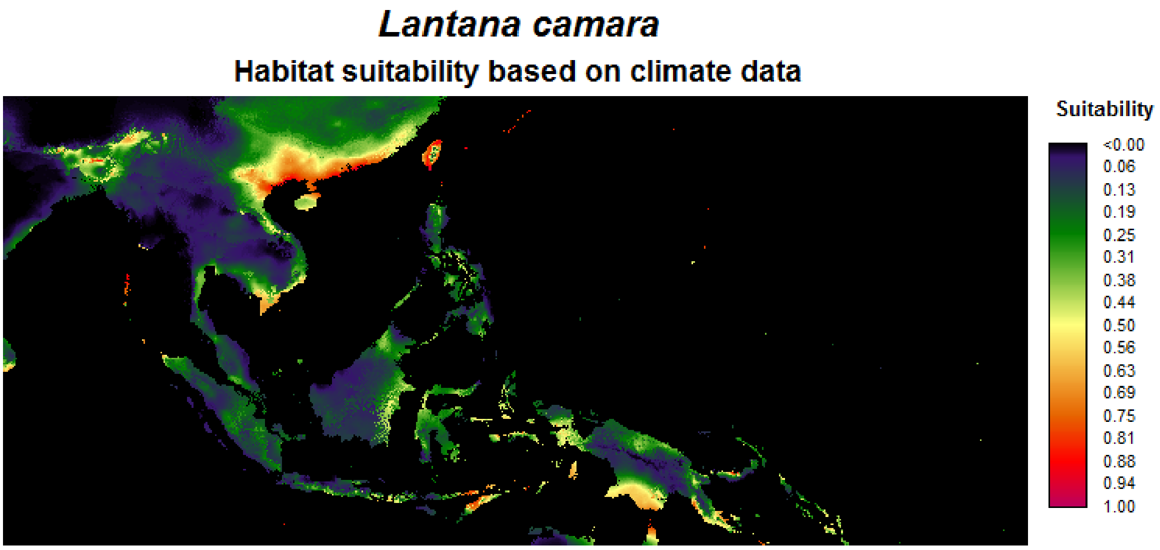
Lantana camara
Habitat suitability based on climate data
Suitability
<0.00
0.06
0.13
0.19
0.25
0.31
0.38
0.44
0.50
0.56
0.63
0.69
0.75
0.81
0.88
0.94
1.00

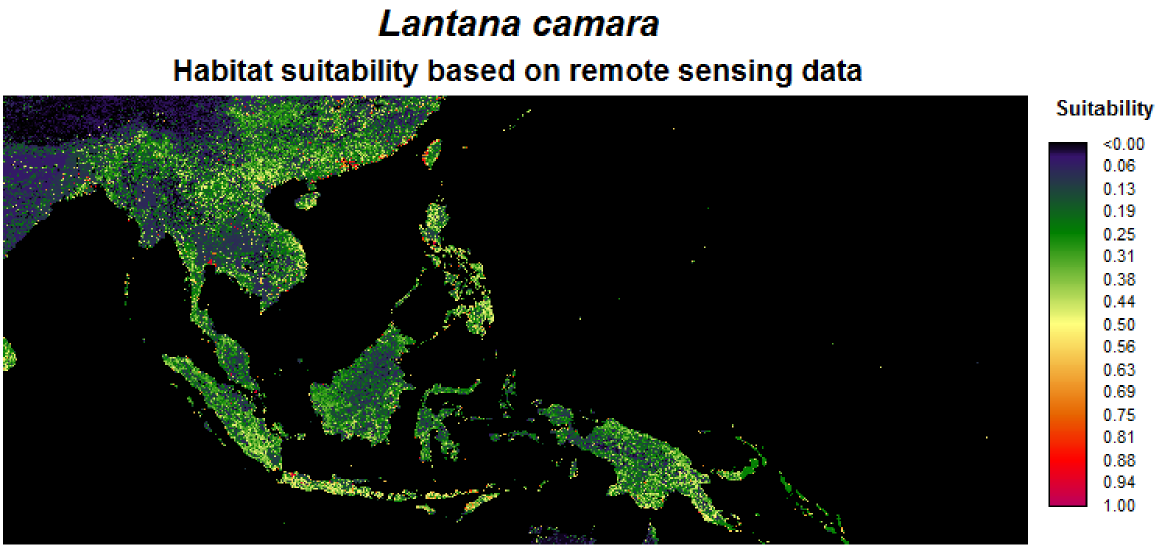
Lantana camara
Habitat suitability based on remote sensing data
Suitability
<0.00
0.06
0.13
0.19
0.25
0.31
0.38
0.44
0.50
0.56
0.63
0.69
0.75
0.81
0.88
0.94
1.00

Leucaena leucocephala

Habitat suitability based on climate and remote sensing data

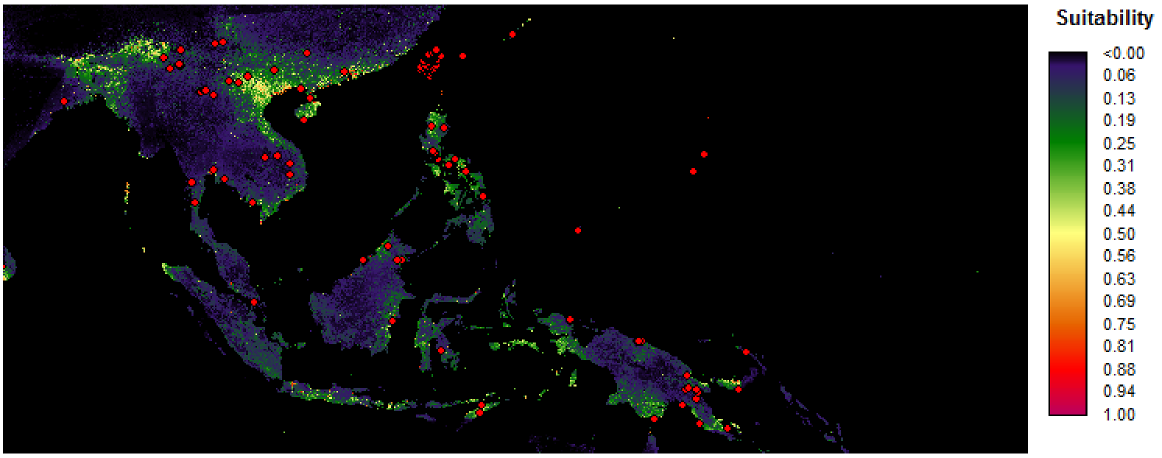

Leucaena leucocephala

Habitat suitability based on climate data

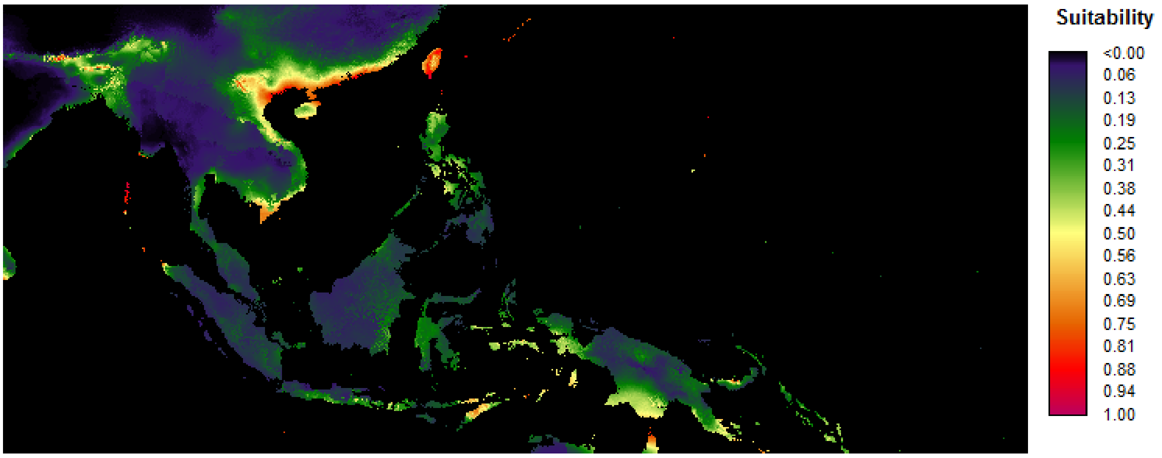

Leucaena leucocephala

Habitat suitability based on remote sensing data

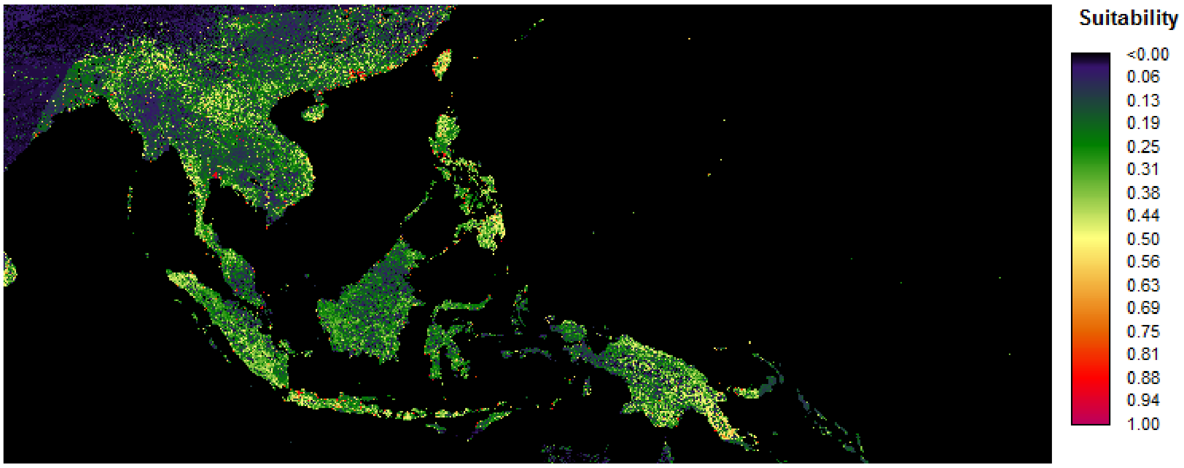

Merremia boisiana

Habitat suitability based on climate and remote sensing data

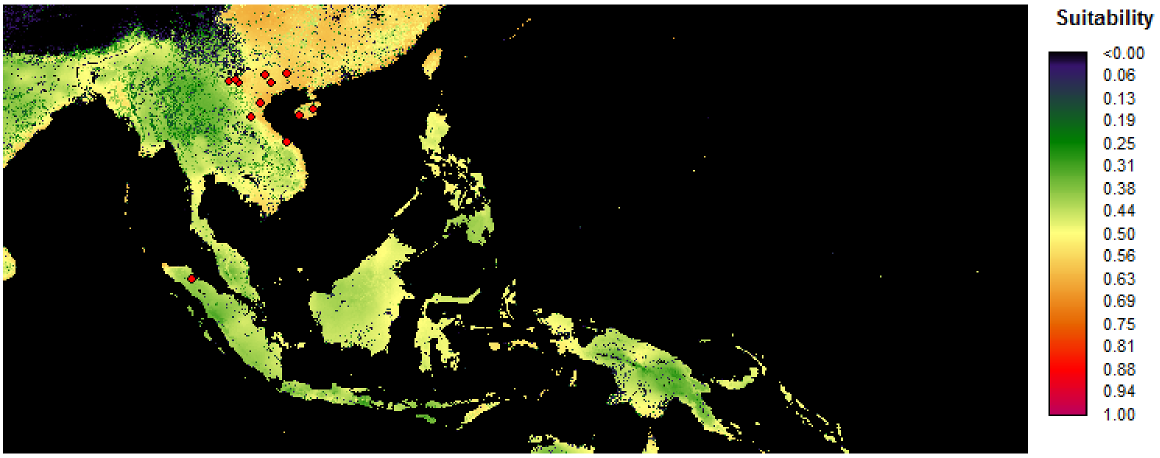

Merremia boisiana

Habitat suitability based on climate data

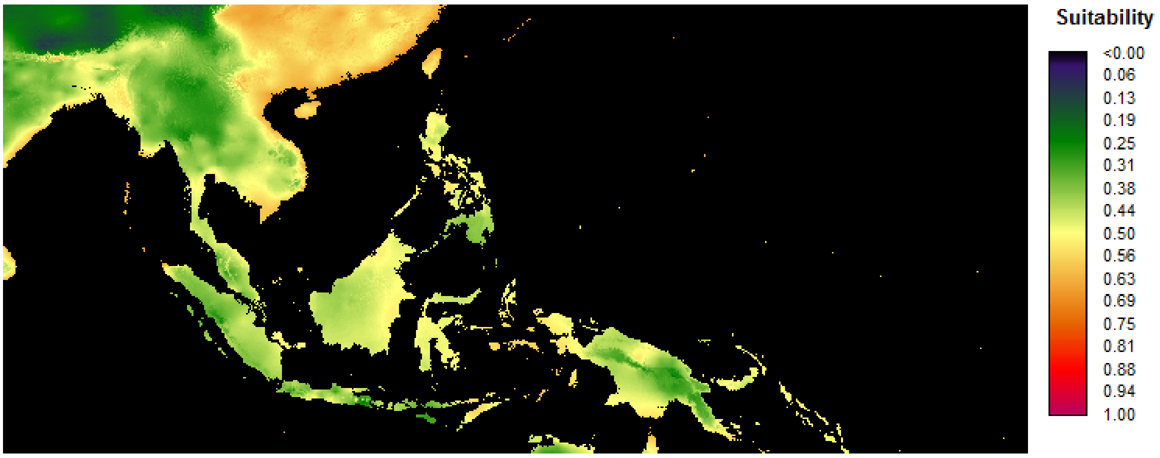

Merremia boisiana

Habitat suitability based on remote sensing data

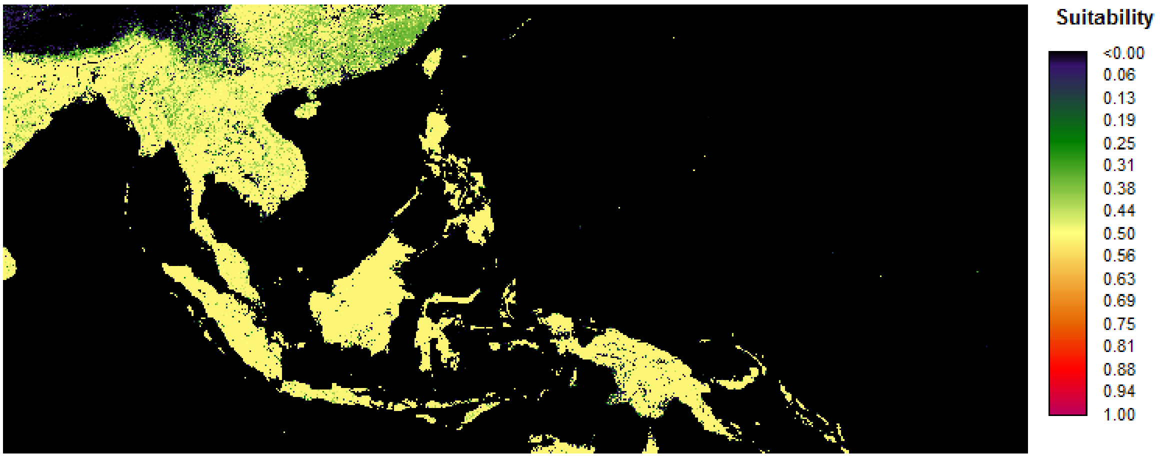

Microstegium ciliatum

Habitat suitability based on climate and remote sensing data

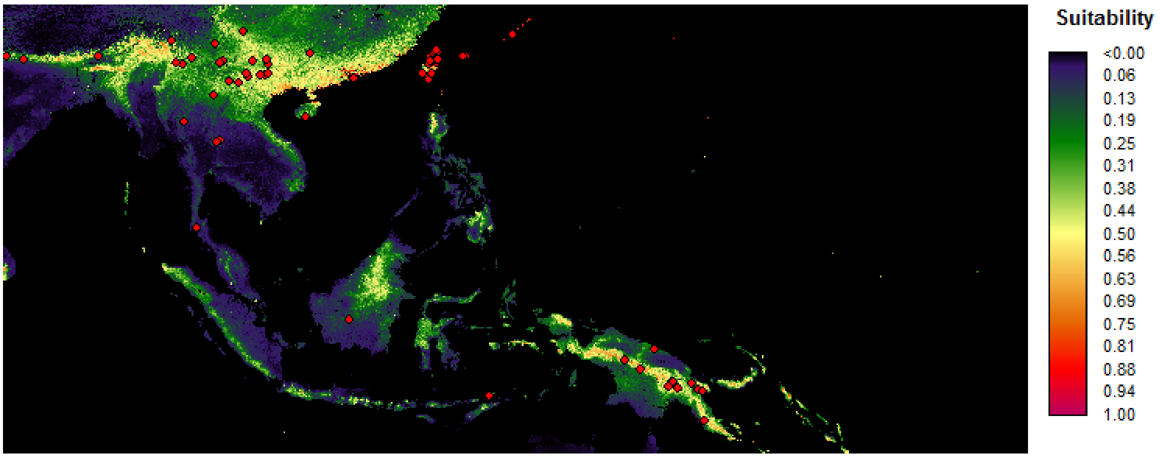

Microstegium ciliatum

Habitat suitability based on climate data

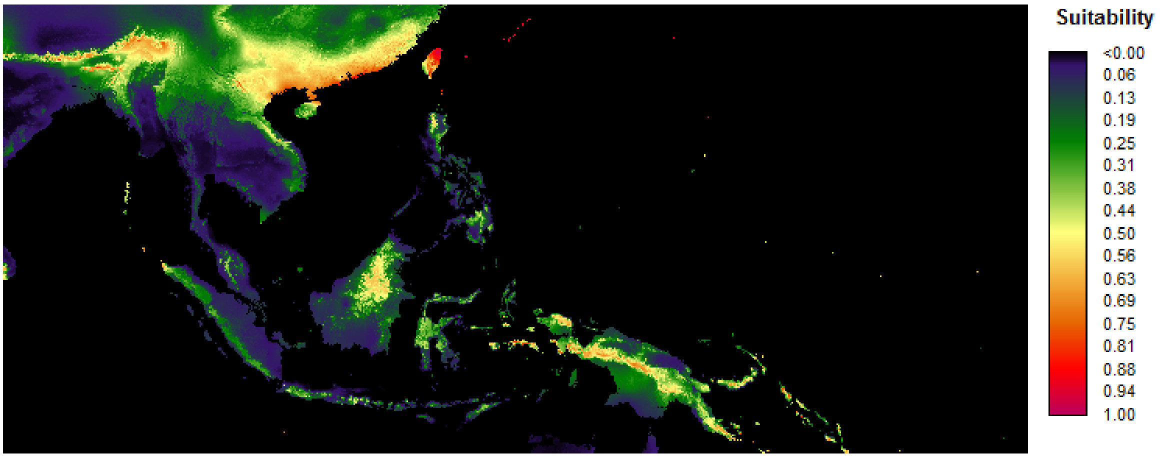

Microstegium ciliatum

Habitat suitability based on remote sensing data

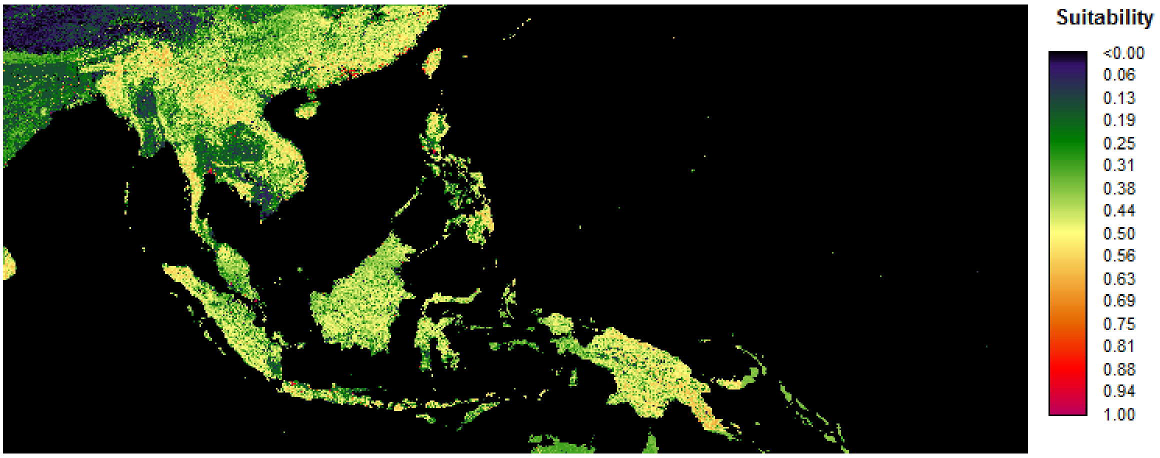

Mikania micrantha
Habitat suitability based on climate and remote sensing data

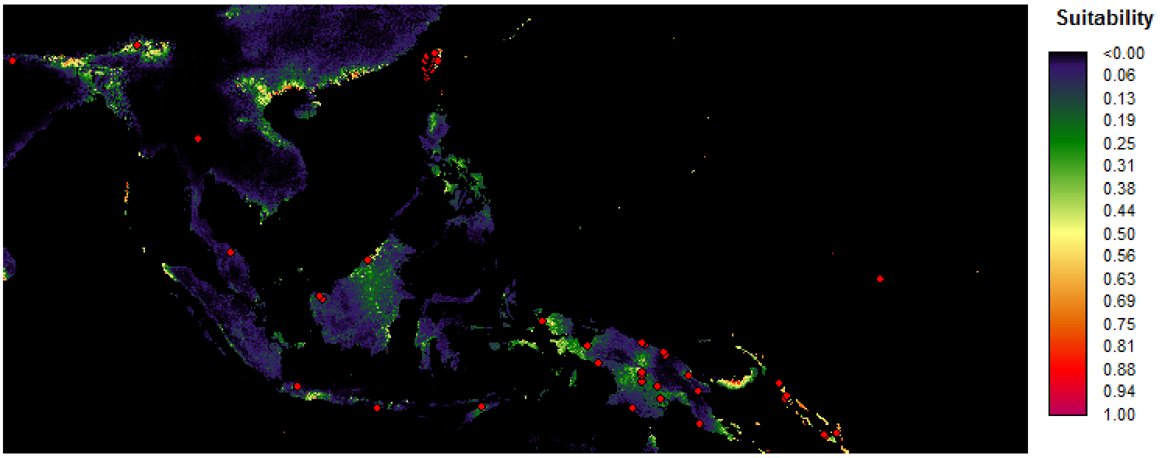

Mikania micrantha
Habitat suitability based on climate data

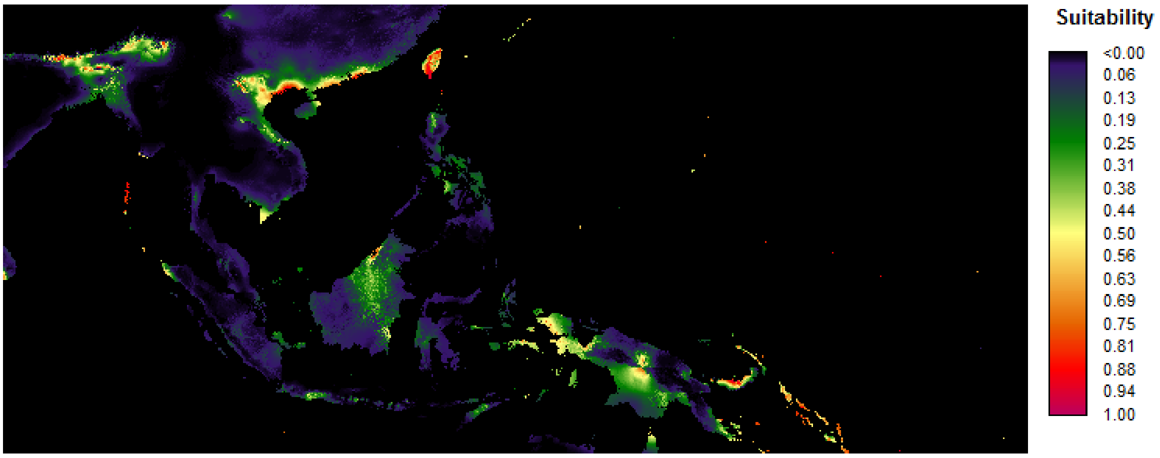

Mikania micrantha
Habitat suitability based on remote sensing data

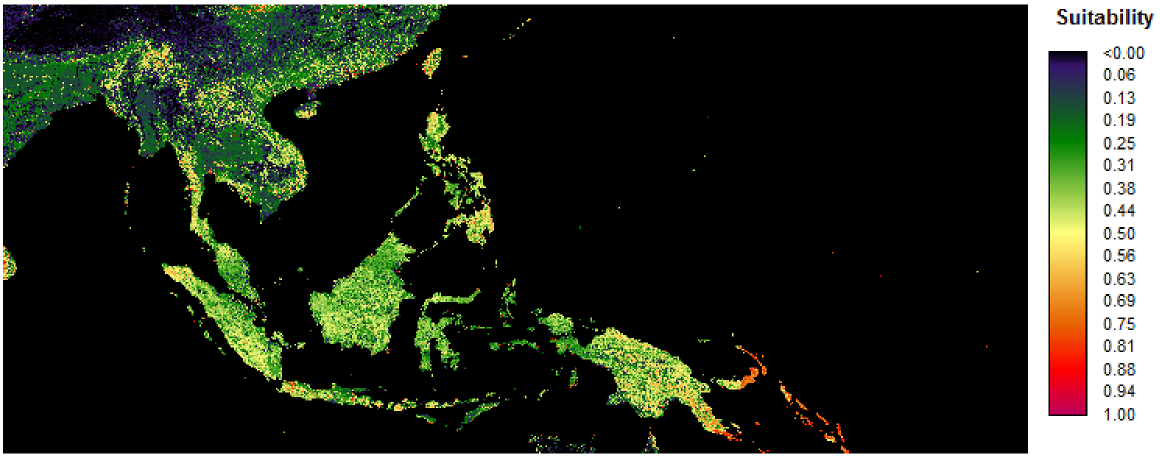

Mimosa diplotricha

Habitat suitability based on climate and remote sensing data

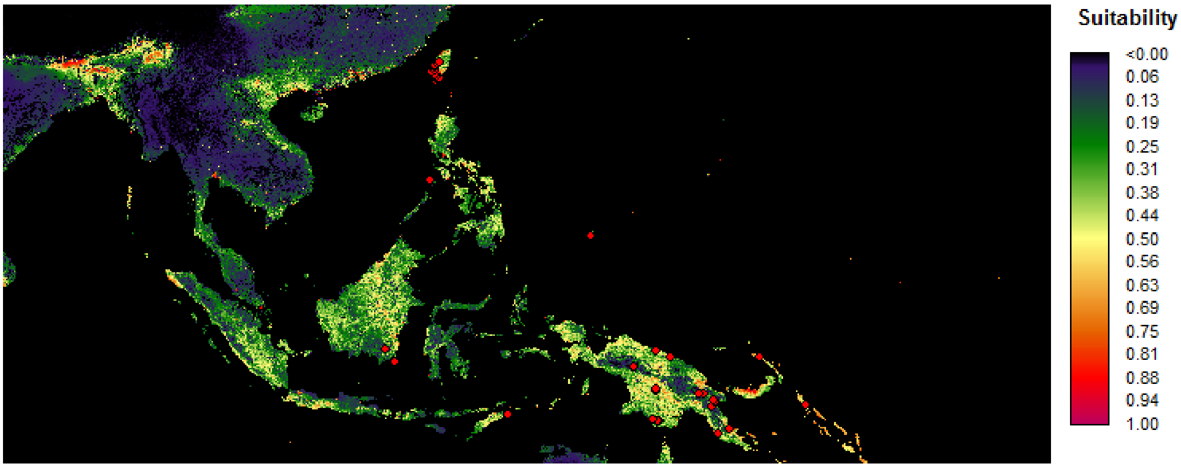

Mimosa diplotricha

Habitat suitability based on climate data

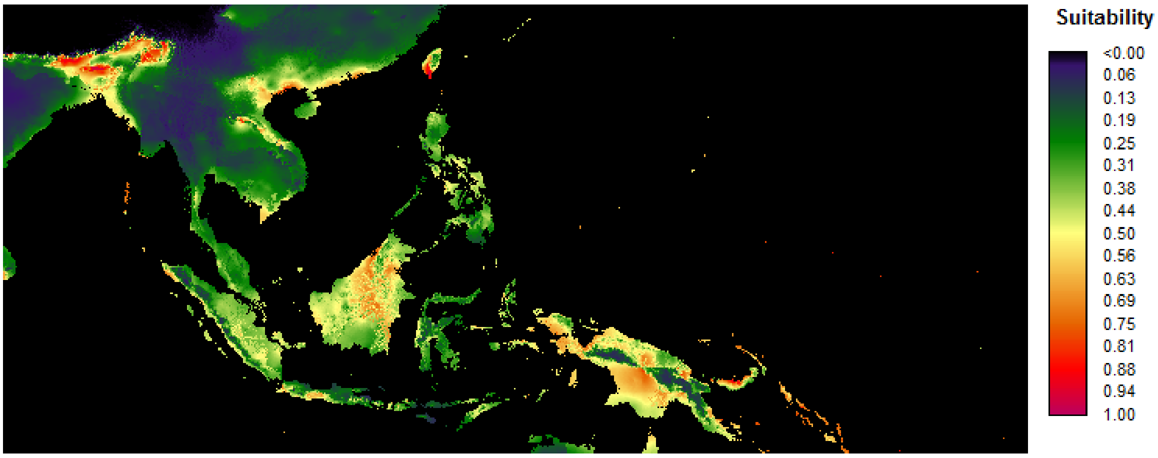

Mimosa diplotricha

Habitat suitability based on remote sensing data

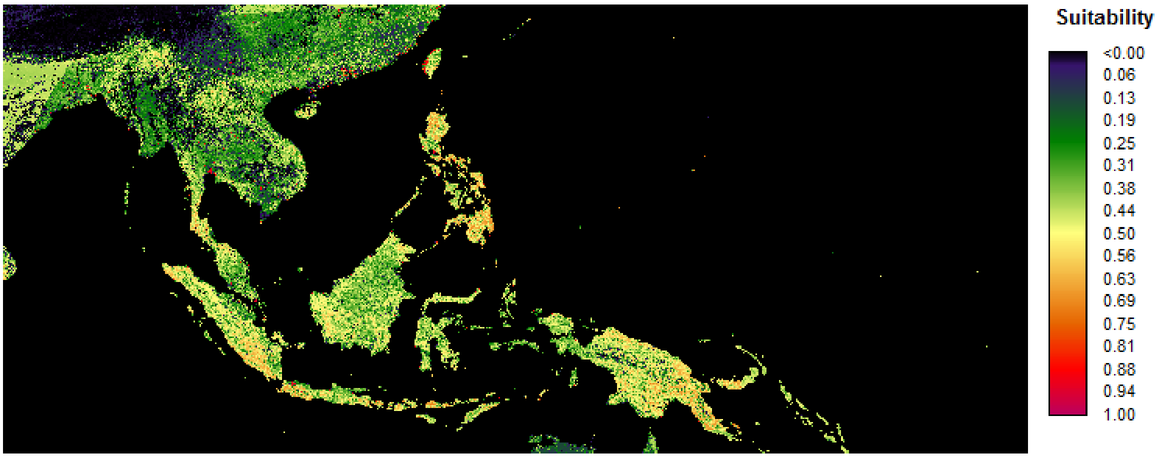

Mimosa pigra

Habitat suitability based on climate and remote sensing data

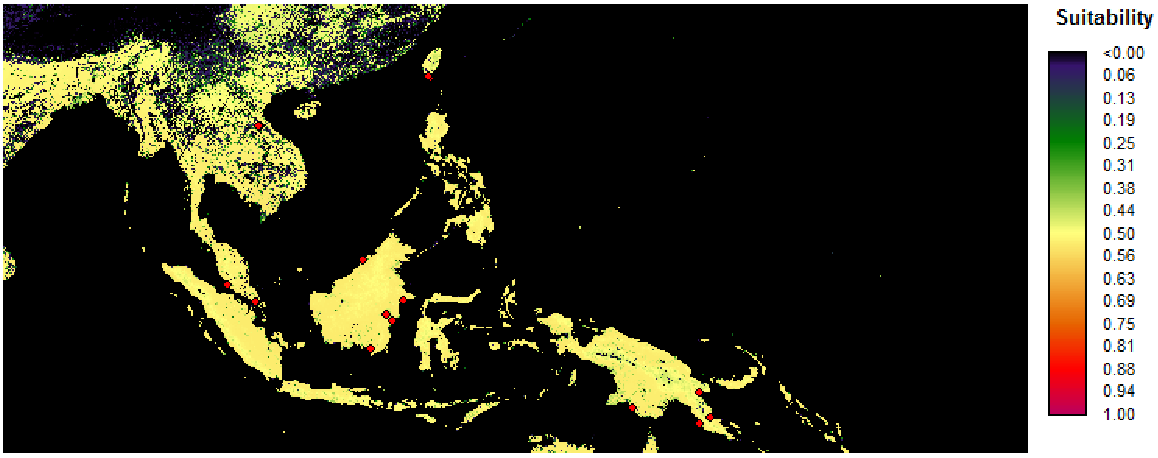

Mimosa pigra

Habitat suitability based on climate data

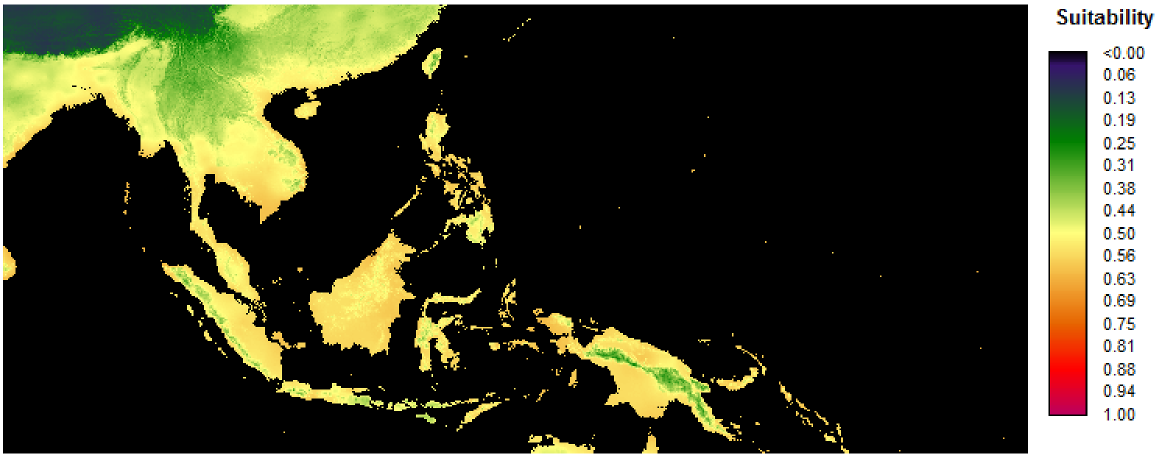

Mimosa pigra

Habitat suitability based on remote sensing data

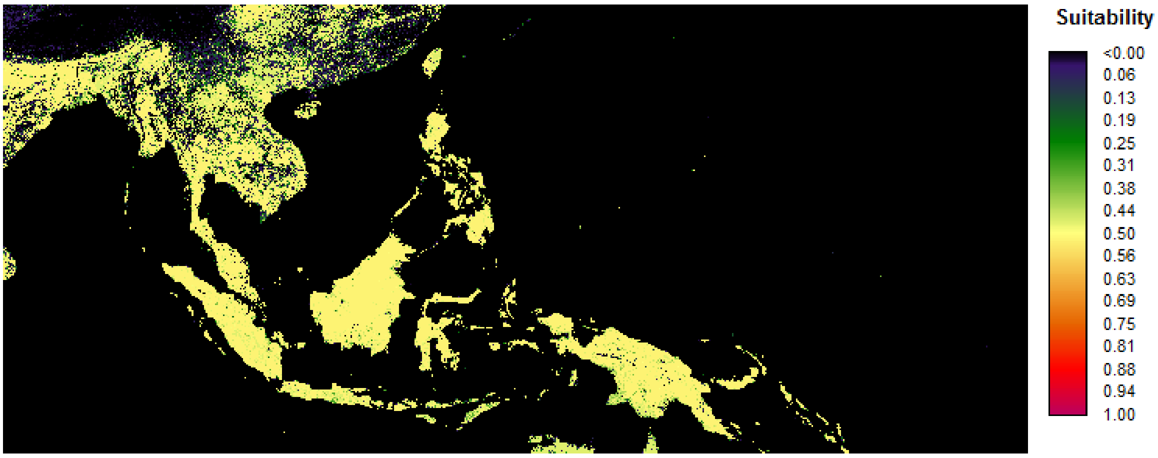

Parthenium hysterophorus

Habitat suitability based on climate and remote sensing data

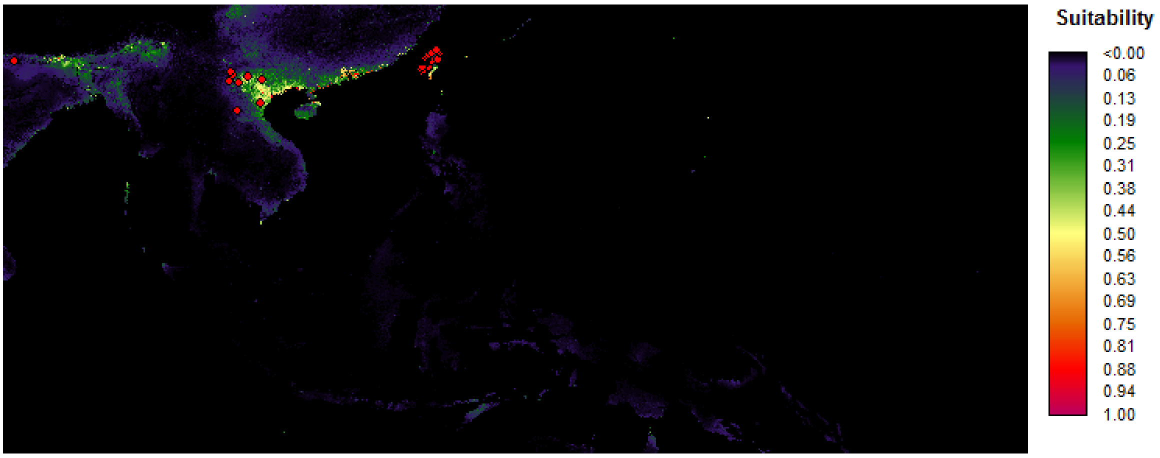

Parthenium hysterophorus

Habitat suitability based on climate data

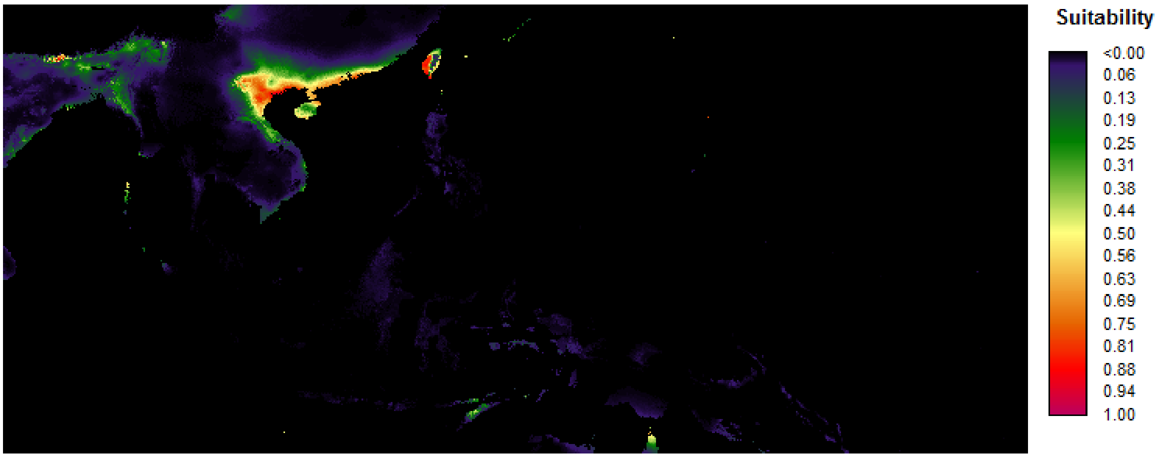

Parthenium hysterophorus

Habitat suitability based on remote sensing data

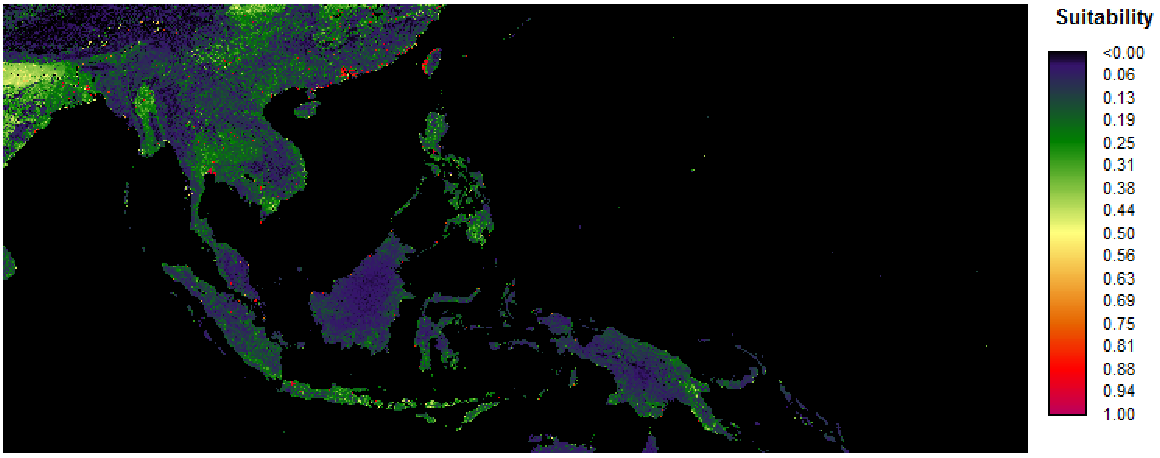

Pueraria montana

Habitat suitability based on climate and remote sensing data

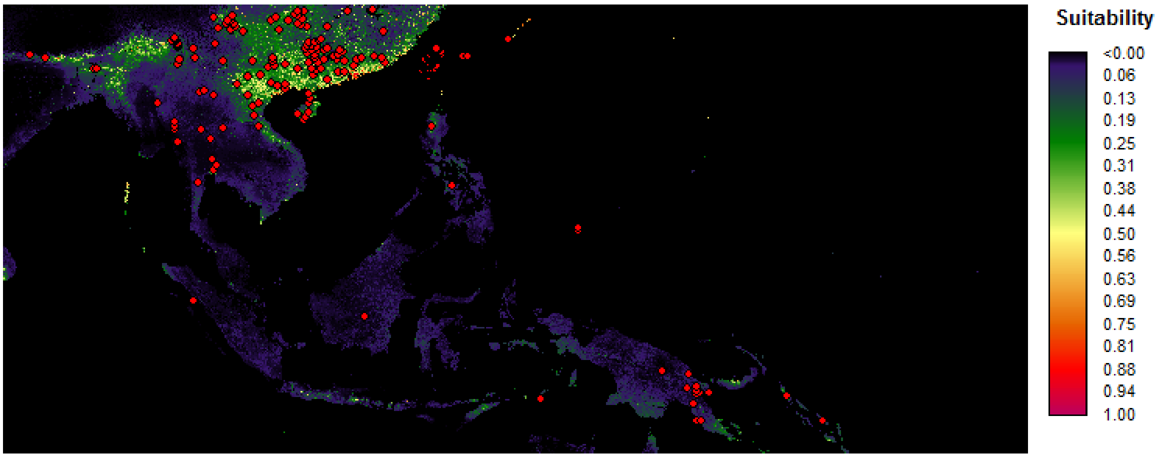

Pueraria montana

Habitat suitability based on climate

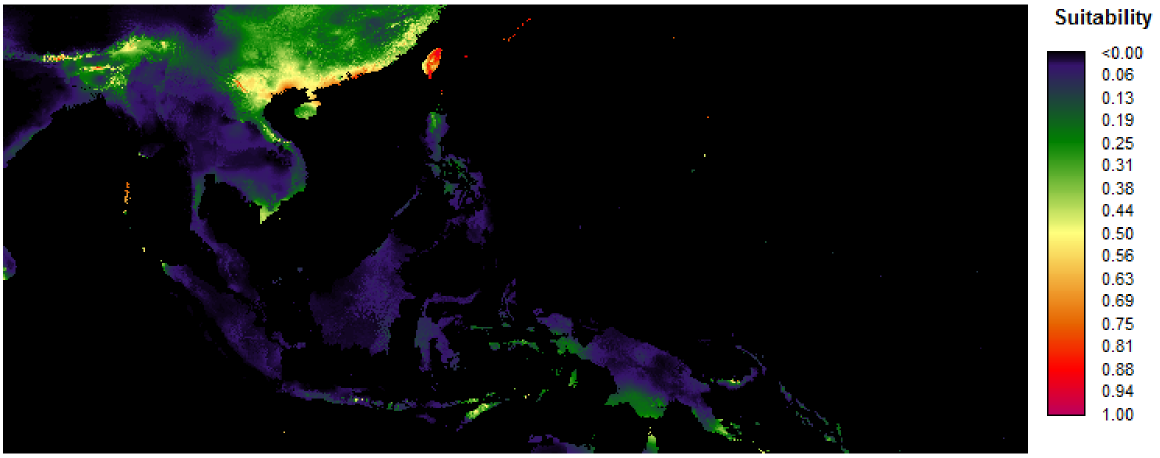

Pueraria montana

Habitat suitability based on remote sensing data

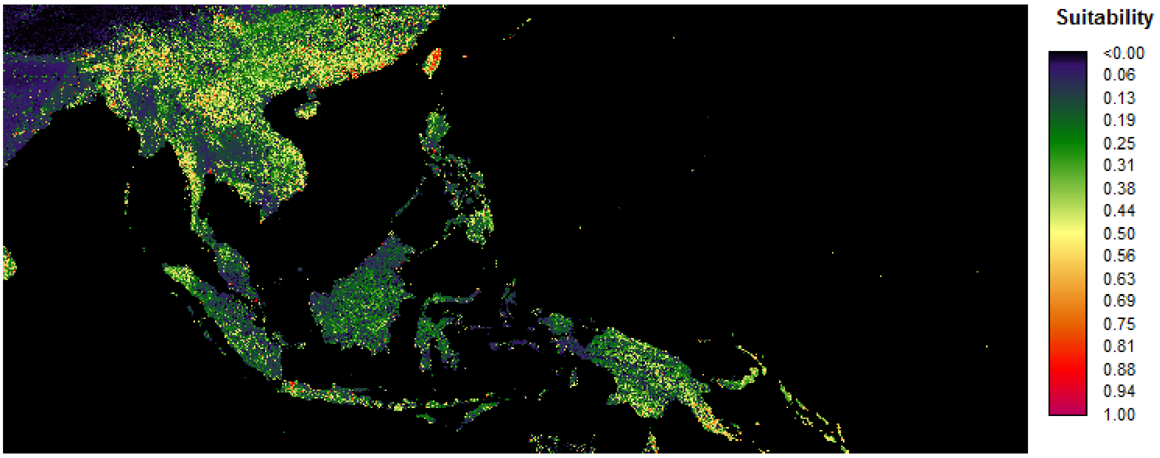

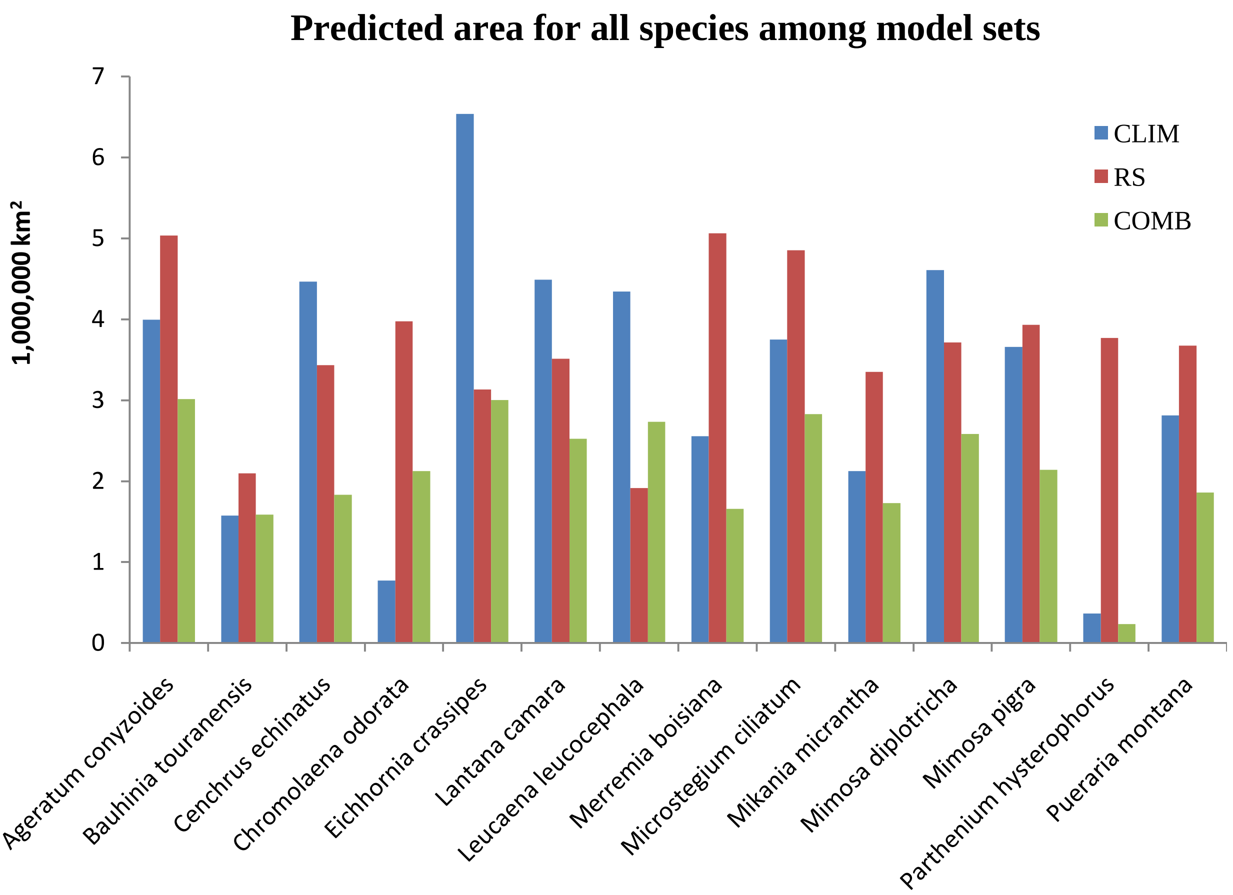
Predicted area for all species among model sets
1,000,000 km²
0
1
2
3
4
5
6
7
CLIM
RS
COMB
Ageratum conyzoides
Bauhinia touranensis
Cenchrus echinatus
Chromolaena odorata
Eichhornia crassipes
Lantana camara
Leucaena leucocephala
Merremia boisiana
Microstegium ciliatum
Mikania micrantha
Mimosa diplotricha
Mimosa pigra
Parthenium hysterophorus
Pueraria montana

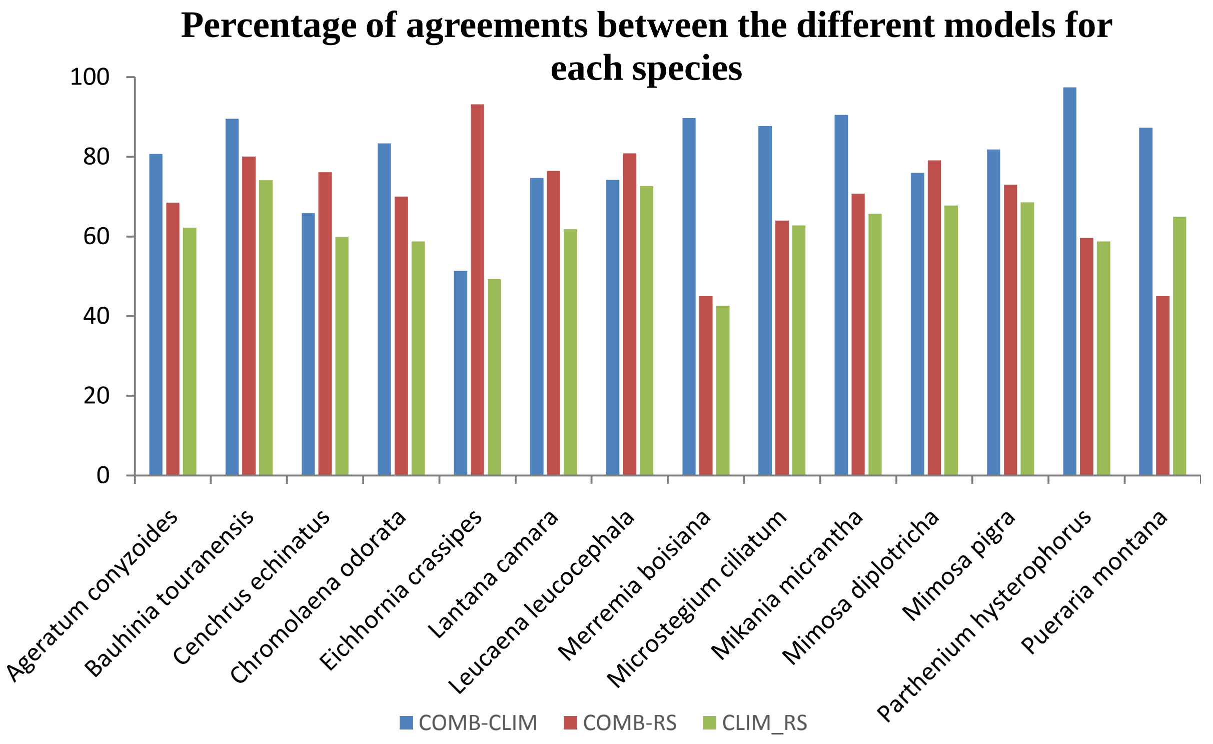
Percentage of agreements between the different models for each species
0
20
40
60
80
100
Ageratum conyzoides
Bauhinia touranensis
Cenchrus echinatus
Chromolaena odorata
Eichhornia crassipes
Lantana camara
Leucaena leucocephala
Merremia boisiana
Microstegium ciliatum
Mikania micrantha
Mimosa diplotricha
Mimosa pigra
Parthenium hysterophorus
Pueraria montana
COMB-CLIM
COMB-RS
CLIM_RS
