# Supplementary material for: Contemporary Remotely Sensed Data Products Refine Invasive Plants Risk Mapping in Data Poor Regions
Source: Front Plant Sci. 2017 May 15;8:770. doi: 10.3389/fpls.2017.00770 (PMC5430062; doi:10.3389/fpls.2017.00770)
Supplement: Supplementary file 3 [file Data_Sheet_3.pdf]

SUPPORTING INFORMATION

Tuyet .T.A Truong, Giles E. St. J. Hardy, Margaret E. Andrew: Contemporary remotely sensed data products refine invasive plants risk mapping in data poor regions

S3: Test AUC and average permutation importance of variables in MaxEnt models developed only with recent species occurrences (post-1992), evaluating the performance of models based on Climate (CLIM), Remote sensing data (RS) and Climate and remote sensing data (COMB).

Table 1: Variability (mean and standard deviation) of species-specific AUC (area under the curve) scores as, evaluated against the withheld test set of 30% of presence records, for invasive weeds in 10 partition runs. Occurrence records were filtered to include only those observed after 1992.

Species*	Training samples	CLIM	RS	COMB
Ageratum conyzoides	82	**0.80 ± 0.02**	0.69 ± 0.04	0.77 ±0.04
Cenchrus echinatus	14	**0.89 ± 0.06**	0.78 ± 0.08	0.72 ± 0.07
Chromolaena odorata	75	0.88 ± 0.02	0.72 ± 0.04	**0.91 ± 0.03**
Eichhornia crassipes	15	0.54 ±0.06	0.71 ± 0.14	**0.74 ± 0.10**
Lantana camara	24	0.75 ± 0.08	0.76 ± 0.07	**0.83 ±0.08**
Leucaena leucocephala	35	**0.84 ± 0.03**	0.72 ± 0.05	0.83 ± 0.07
Microstegium ciliatum	14	**0.91 ± 0.05**	0.71 ± 0.12	0.74 ± 0.10
Mikania micrantha	76	0.91 ± 0.02	0.79 ± 0.05	**0.92 ± 0.03**
Mimosa diplotricha	29	0.84 ± 0.04	0.79 ± 0.06	**0.85 ± 0.03**
Parthenium hysterophorus	38	**0.98 ± 0.01**	0.85 ± 0.05	0.98 ± 0.01
Pueraria montana	52	0.83 ± 0.03	0.76 ± 0.03	**0.85 ± 0.05**
Mean		**0.83 ± 0.01**	0.75 ± 0.05	**0.83 ± 0.08**

**Bauhinia touranensis*, *Merremia boisiana*, and *Mimosa pigra* were not included due to less than 10 occurrence records after filtering. AUC values for the best-performing model for each species are indicated in bold.

Table 2. Summary of the mean permutation importance of the 11 species modelled using only recent occurrence data. Occurrence records were filtered to include only those observed after 1992. SD is standard deviation. Mean values were calculated from the average of 11 species. Values in bold indicate variables with the highest average importance in COMB, CLIM, and RS models

	COMB			CLIM			RS		
	Mean		***SD***	***Mean***		***SD***	***Mean***		***SD***
GPP_CV	2.86	±	2.44				**19.58**	**±**	**12.64**
GPP_Mean	4.22	±	6.64				**10.10**	**±**	**10.17**
Soil pH	1.10	±	1.24				2.80	±	4.83
Barren	1.63	±	1.41				3.24	±	4.30
Cultivated vegetation	**5.12**	**±**	**1.09**				**9.73**	**±**	**7.16**
Deciduous broad leaf trees	4.20	±	2.37				**8.45**	**±**	**6.80**
Evergreen broad leaf trees	**5.66**	**±**	**15.01**				**7.82**	**±**	**7.80**
Evergreen needle leaf trees	3.07	±	2.97				5.33	±	5.74
Herbaceous vegetation	**8.03**	**±**	**21.57**				**16.29**	**±**	**4.36**
Mixed trees	**5.55**	**±**	**12.17**				**9.26**	**±**	**10.50**
Open water	0.37	±	3.01				1.27	±	1.48
Regular flooded vegetation	0.93	±	7.22				0.96	±	1.68
Shrubs	1.17	±	3.61				3.11	±	3.57
Urban	0.70	±	8.23				2.06	±	2.86
Annual Mean Temperature	2.02	±	5.04	8.18	±	4.55			
Mean Diurnal Range	**25.51**	**±**	**5.80**	**46.27**	**±**	**1.37**			
Isothermality	**11.35**	**±**	**11.93**	**14.05**	**±**	**4.96**			
Annual precipitation	1.04	±	0.39	2.82	±	18.64			
Precipitation of wettest month	1.02	±	1.55	1.76	±	8.60			
Precipitation seasonality	2.01	±	1.69	3.35	±	16.76			
Precipitation of warmest quarter	**12.46**	**±**	**1.03**	**18.11**	**±**	**12.62**			
